# Is exposure to formaldehyde in air causally associated with leukemia?—A hypothesis-based weight-of-evidence analysis

**DOI:** 10.3109/10408444.2011.560140

**Published:** 2011-06-02

**Authors:** Lorenz R Rhomberg, Lisa A Bailey, Julie E Goodman, Ali K Hamade, David Mayfield

**Affiliations:** 1 Gradient, Cambridge, Massachusetts, USA; 2 Gradient, Seattle, Washington, USA

**Keywords:** Epidemiology, formaldehyde, genotoxicity, hazard identification, leukemia, risk assessment

## Abstract

Recent scientific debate has focused on the potential for inhaled formaldehyde to cause lymphohematopoietic cancers, particularly leukemias, in humans. The concern stems from certain epidemiology studies reporting an association, although particulars of endpoints and dosimetry are inconsistent across studies and several other studies show no such effects. Animal studies generally report neither hematotoxicity nor leukemia associated with formaldehyde inhalation, and hematotoxicity studies in humans are inconsistent. Formaldehyde's reactivity has been thought to preclude systemic exposure following inhalation, and its apparent inability to reach and affect the target tissues attacked by known leukemogens has, heretofore, led to skepticism regarding its potential to cause human lymphohematopoietic cancers. Recently, however, potential modes of action for formaldehyde leukemogenesis have been hypothesized, and it has been suggested that formaldehyde be identified as a known human leukemogen. In this article, we apply our hypothesis-based weight-of-evidence (HBWoE) approach to evaluate the large body of evidence regarding formaldehyde and leukemogenesis, attending to how human, animal, and mode-of-action results inform one another. We trace the logic of inference within and across all studies, and articulate how one could account for the suite of available observations under the various proposed hypotheses. Upon comparison of alternative proposals regarding what causal processes may have led to the array of observations as we see them, we conclude that the case fora causal association is weak and strains biological plausibility. Instead, apparent association between formaldehyde inhalation and leukemia in some human studies is better interpreted as due to chance or confounding.

## Contents

Abstract…………555
1. Introduction and background…………557
2. Hypothesis-based weight-of-evidence (HBWoE) evaluation…………558
2.1. Overview of approach…………558
2.1.1. Hill Criteria and the concept of “accounts”…………559
2.2. HBWoE methodology…………561
3. Overview of HBWoE as applied to formaldehyde and leukemogenesis…………562
4. Weight of epidemiology evidence regarding the association between formaldehyde exposure and leukemia…………563
4.1. Overview of epidemiology investigations…………563
4.2. Endpoint-by-endpoint analysis…………571
4.2.1. All lymphohematopoietic cancers…………571
4.2.2. Cancer of lymphoid origin…………574
4.2.3. Leukemia…………574
4.2.4. Lymphatic leukemia…………578
4.2.5. Hematopoietic cancer of non-lymphoid origin…………578
4.2.6. Myeloid leukemia…………581
4.2.7. Other unspecified leukemia…………582
4.2.8. Hodgkin's lymphoma, non-Hodgkin's lymphoma, and multiple myeloma…………582
4.3. HBWoE evaluation of epidemiology studies…………582
4.3.1. Cancer outcome assessments likely lead to disease misclassification…………585
4.3.2. Exposure assessments likely affected by exposure measurement error or misclassification…………585
4.3.3. Exposures to other chemicals in the work place may have confounded results…………586
4.3.4. Exposure-response associations within and among studies are not consistent…………586
4.3.5. Statistical limitations may have led to spurious associations…………590
4.3.6. The latency argument proposed by Beane Freeman et al. (2009) appears to be a post hoc explanation for the observed effects…………590
4.3.7. Recent formaldehyde meta-analyses do not support an association between formaldehyde exposure and leukemia…………591
4.4. Summary…………592
5. Weight of evidence regarding hematotoxicity from formaldehyde exposure…………593
5.1. Formaldehyde hematotoxicity in animals…………593
5.1.1. Hematology…………593
5.1.2. Leukemia…………594
5.2. Formaldehyde hematotoxicity in humans…………595
5.3. Hypothesis-based weight-of-evidence evaluation of formaldehyde hematotoxicity studies…………596
5.3.1. Key animal studies do not provide strong evidence of an association between formaldehyde exposure and hematotoxicity and leukemia…………599
5.3.1.1. Hematology…………599
5.3.1.2. Leukemia…………599
5.3.2. Key human studies do not provide strong evidence of an association between formaldehyde exposure and hema totoxicity…………601
5.3.3. If formaldehyde causes leukemia in humans, it is likely due to a mechanism that is different from that observed with known leukemogens…………602
5.3.4. There are alternative explanations for the pancytopenia reported by Zhang et al. (2010b) and the leukopenia reported by other studies…………603
5.3.4.1. Subjects exposed to formaldehyde share common immunology markers with subjects having dermatitis or other inflammatory conditions…………603
5.3.4.2. A recent respiratory infection can result in hematological changes—Subjects with exposure to formalde hyde in the study by Zhang et al. (2010b) were more likely than control subjects to have had recent respiratory tract infections…………604
5.3.4.3. Other unmeasured potential confounders…………604
5.4. Summary…………605
6. Weight of evidence regarding a plausible mode of action for formaldehyde leukemogenesis…………606
6.1. Formaldehyde toxicokinetics…………606
6.2. Formaldehyde genotoxicity…………607
6.2.1. DNAadducts and protein cross-links…………607
6.2.2. Clastogenic and cytogenetic effects…………608
6.3. HBWoE evaluation of the proposed modes of action for formaldehyde as a leukemogen…………608
6.3.1. There is no consistent evidence that inhaled formaldehyde induces genotoxicity in bone marrow, NALT, or peripheral HSCs that might lead to leukemia…………609
6.3.1.1. Bone marrow…………609
6.3.1.2. Stem cells in the NALT…………609
6.3.1.3. Circulating peripheral HSCs…………610
6.3.2. Formaldehyde exposure would have to be very high to induce DNA damage above endogenous levels in the bone marrow, NALT, or circulating HSCs, and would likely be associated with a high degree of irritation…………612
6.3.3. Circulating HSCs may not readily home back to healthy bone marrow to cause leukemia…………612
6.4. Summary…………613
7. Discussion…………614
Acknowledgments…………616
Declaration of interest…………616
References…………617

## 1. Introduction and background

Formaldehyde is produced naturally by the human body. It is also a chemical intermediate used in the production of some plywood adhesives, fertilizer, paper, and urea-formaldehyde resins (Agency for Toxic Substances and Disease Registry [ATSDR], 1999). It is found (as a preservative or impurity) in many products around the home, such as antiseptics, medicines, and cosmetics/personal hygiene products (ATSDR, 1999). Formaldehyde is also used for embalming and preserving biological specimens (United States Environmental Protection Agency [US EPA], 2010). Sources of exposure to formaldehyde include occupational exposure during use or production of materials containing formaldehyde; cigarette smoke; off-gassing from manufactured wood products in new mobile homes; and other new products found in homes (e.g., fiberglass, carpets, and paper products) (ATSDR, 1999).

Studies have shown that exposure to high concentrations of formaldehyde in air results in nasal cancer in rats. Some studies in humans exposed to lower concentrations of formaldehyde in air in the workplace found increased incidence of nasopharyngeal cancer, but other studies have not found an increased risk of these cancers in formaldehyde-exposed workers (ATSDR, 1999; Marsh and Youk, 2005; Marsh, 2007a, 2007b; Bachand et al., 2010; US EPA, 2010). More recently, there has been increased concern and scientific debate regarding the potential for exposure to formaldehyde in air to cause lymphohematopoietic cancers in humans, particularly leukemias (US EPA, 2010; Bachand et al., 2010; Beane Freeman et al., 2009; Hauptmann et al., 2009; Zhang et al., 2009, 2010a, 2010b; Pyatt et al., 2008; Golden et al., 2006; Heck and Casanova, 2004).

The concern for formaldehyde-induced leukemogen-esis stems from a few epidemiology studies reporting an association between formaldehyde exposure and increased mortality from leukemia (e.g., Beane Freeman et al., 2009; Hauptmann et al., 2009), although other studies show no such effects (e.g., Bachand et al., 2010; Pinkerton et al., 2004). The studies reporting associations have shortcomings, including poor disease classification and unverified estimates of exposure. Studies have been conducted to examine the potential for formaldehyde in air to induce hematotoxicity in animals and humans and leukemia in animals. The animal studies generally reported neither hematotoxicity (Monticello et al., 1989; Appelman et al., 1988; Holmstrom et al., 1989; Kerns et al., 1983; Kamata et al., 1997; Woutersen et al., 1987; Til et al., 1988, 1989; Johannsen et al., 1986) nor leukemia (Albert et al., 1982; Kerns et al., 1983; Sellakumar et al., 1985; Kamata et al., 1997; Feron et al., 1988; Til et al., 1989; Tobe et al., 1989; Takahashi et al., 1986) associated with formaldehyde exposure. Although a few animal studies reported changes in one or more hematology parameters (Dean et al., 1984; Tobe et al., 1989; Vargova et al., 1993), two animal studies reported leukemias (Soffritti et al., 1989, 2002), and a few human study findings were consistent with hematotoxicity from exposure to formaldehyde (Tang et al., 2009; Zhang et al., 2010b), these studies were inconsistent with other study findings and/or plagued by possible confounding.

Despite the lack of substantial and consistent epide-miological and toxicological evidence for formaldehyde leukemogenesis, US EPA has concluded that formaldehyde should be deemed a known human leukemogen (US EPA, 2010), citing possible modes of action put forth by Zhang et al. (2009, 2010a). The three proposed modes of action involve formaldehyde: (1) migrating to and directly targeting bone marrow hematopoietic stem cells; (2) targeting nasal stem cells (nasal-associated lym-phoid tissue, or NALT) which then are released from the nasal passage, circulate in the blood, and are eventually incorporated into bone marrow, leading to leukemia; or (3) targeting circulating hematopoietic stem cells, which then migrate back to bone marrow, eventually leading to leukemia. The proposed modes of action, however, find little support in the current literature; there is a large body of evidence indicating that inhaled formaldehyde (at reasonably high exposure levels in humans, 2 ppm) does not move beyond the nasal respiratory mucosa to increase levels in the blood and does not cause DNA damage or cellular transformation (in the bone marrow, circulating hematopoietic stem cells, or the NALT) beyond the portal of entry (Lu et al., 2010, 2011; Moeller et al., 2011; Andersen et al., 2010). These results suggest strongly that if formaldehyde is not getting beyond the nasal respiratory mucosa (as indicated by its lack of genotoxicity and cellular transformation beyond the nasal epithelial cells), it is not likely to induce leukemogenesis (either via genotoxicity or another carcinogenic mode of action).

Acceptance of formaldehyde as a human leukemogen on the strength of observed associations of exposure and effect seen in the epidemiology studies requires accepting the existence of underlying biological processes that embody the causal forces, whether or not these underlying causal processes are identified. This is true of any epidemiological association that is deemed causal, but what is notable about formaldehyde and leukemia is that current understanding both of leukemogenesis by other agents (entailing toxicity to the marrow and genotoxic attack on hematopoietic precursor cells found there) and of formaldehyde kinetics (which appear to preclude such effects distal to the respiratory tract) raises the issue of whether the phenomena observed in the human studies can be interpreted as causal and consistent with known biology. It is not simply that the underlying biological causal processes are unproven—or even hypothetical— but rather, at least at first view, there seems to be no scientifically plausible means for sufficient causal processes to operate based on what is believed to be true about formaldehyde and hematopoiesis.

In the present paper, we evaluate the scientific data relevant to the potential causal association between exposure to formaldehyde in air and leukemia in humans using the structured hypothesis-based weight-of-evidence (HBWoE) approach we have developed and applied elsewhere (Rhomberg et al., 2010). The HBWoE methodology is described below.

## 2. Hypothesis-based weight-of-evidence (HBWoE) evaluation

### 2.1. Overview of approach

Before discussing the evidence regarding formaldehyde's potential leukemogenicity, it is useful to address our overall approach to the weight-of-evidence question by outlining our method, explaining how it differs from other approaches, and setting out why we feel our chosen approach has value. Weed (2005) points out that the term “weight of evidence” is often used loosely; he calls on practitioners to articulate what they mean by the phrase and to specify their approach. Analyses of various technical approaches to weight of evidence have been offered by Krimsky (2005) and Linkov et al. (2009). Clearly, professional judgment is involved, but it is not enough simply to name the evidence at hand and then announce one's conclusion. Our method aims to make the reasoning process and bases for judgments explicit and transparent so that, even if other observers differ with our conclusions, debate can focus on the soundness of the inferences and their connections to study results, rather than devolve into ad hominem arguments about the identity and perspectives of the judges. That is, we seek to make expert judgment a public process by focusing on the *logic* of the process—not just the *outcome.* Ideally, rational evaluation of objective evidence and scientific scrutiny of such evaluation should be the criterion for knowledge, not simple authority of the interpreter.

For some, weight of evidence may connote a process for coming to a yes/no decision in the face of incomplete or contradictory evidence—to agree on a conclusion despite lack of definitive proof—but we seek a method, rather, that arrives at a useful and reasoned characterization of the relative scientific credence that should be placed in alternative interpretations of the data at hand in view of the arguments for and against each alternative. That is, we aim to communicate uncertainty about conclusions so as to enable productive discussion about subsequent decisions.

A good weight-of-evidence analysis should attend to all the relevant data, and not simply cite studies (or particular outcomes within studies) that tend to support or refute a conclusion. The frequent practice of reviewing literature by naming the positive or otherwise notable outcomes of the included studies, emphasizing findings by the studies' authors, and leaving the negative results for other endpoints or measures of effect implicit can bias evaluations when studies are positive and negative for different endpoints. The analysis should entail an endpoint-by-endpoint comparative approach, on the grounds that true causal effects should be specific (particular endpoints, not one or another of a set of arguably related endpoints) and repeatable (within the limits of study uncertainty and power). Although study quality and design strengths and shortcomings should be noted, we favor an approach that does not reject outright less-than-ideal studies (the outcomes of which maybe informative nonetheless) but, rather, tempers the conclusions drawn. What makes poorer studies less informative is a decreased ability to distinguish between the causative, face-value interpretation of outcomes and the alternative interpretation that the results are spurious because of intrusion of factors not adequately eliminated as possible influences. Thus, the rational and transparent way to down-weight poorer studies is to consider the impact of this ambiguity as one evaluates alternative interpretations of the data, using the patterns of concordance or lack thereof with other studies as part of the evaluation of the likelihood that the study in question has misled us or informed us.

We also seek an approach that integrates inferences across different and diverse kinds of data that can tie together inference based on epidemiology, animal testing, and mode-of-action and pharmacokinetic data. Too often, in our view, these different realms of inquiry are approached separately—each subset of data evaluated within its own realm and according to its own standards—and only then the conclusions are brought together for synthesis. This approach fails to take advantage of the ways in which information from one realm can and should affect interpretation of data within another. For instance, judgments about whether patterns of association seen in human studies represent a causal connection of chemical exposure and disease ought to be based not only on the concordance and repeatability of such patterns among human studies, they also should consider whether animal studies show signs of the operation of the underlying biological processes. Human data have the advantage of greater relevance to the immediate question at hand, but they suffer characteristically from imprecise measures of exposure and effect, and, being uncontrolled and observational, from the difficulty of eliminating possible extraneous influential factors. Animal studies can be controlled more precisely and the underlying biology can be probed more thoroughly, but the relevance of these studies is indirect and only useful to the degree that the animals share underlying causative processes with humans. Since species-specific effects are known in both humans and particular species or strains of experimental animals, lack of concordance of effect across human and animal studies is not a definitive refutation of the proposed causative process, but the reasons for and plausibility of such species differences or other non-concordant outcomes becomes part of the evaluation of correspondence of hypotheses.

An often-overlooked aspect of weight-of-evidence evaluation is the importance of noting when causative explanations have been accommodated to account for results already in hand and when post hoc additions or modifications to hypotheses have been constructed to explain what might otherwise be contradictory findings. Such modifications of explanatory models as a result of new data are valid parts of scientific discovery as we seek explanations and insights into possible underlying causes through the examination of the patterns of phenomena, but one needs to distinguish such a creative, hypothesis-generating process from the subsequent testing of those hypotheses with results that were not used in formulating the proposed model of causes. To the extent that hypotheses are supportable only with such added assumptions and interpretations, even if these additions are plausible and even if the data are then fully in accord with the hypothesized explanations, this constitutes weaker support than if the tentative explanations preceded, and were only later confirmed by, the data.

We have developed an approach to the above questions that we term “hypothesis-based weight of evidence” (or HBWoE). It is hypothesis based in the sense that its critical aspect is to specify the hypothesized basis for using information at hand to infer the existence of the ability of an agent to cause human health impact. The “hypothesis” referred to in the name “hypothesis-based weight of evidence” consists of the proposed basis for using the cited study results as evidence of human risk. That is, one names the study observations that are being proposed as giving insights into human risk and also names the proposed basis for how those observations could be interpreted as informative about human risk potential. This hypothesized basis can be specific in its biological mode-of-action underpinnings, but it can also be more general. For instance, one might base the proposal that an agent is a human carcinogen on observations of its carcinogenicity in animal studies on the grounds that rodents and humans share a good deal of common mammalian biology and the body of observations about how frequently positive animal tests are found for agents with direct human evidence for carcinogenicity. The strength of such an inference would be judged in view of our experience from other agents regarding how often common biology indeed seems to be operating in human and animal disease, the frequency of concordant and discordant results, and the consistency of animal tests observed for the particular chemical at hand.

The hypothesized basis for inference about human risk from particular data should be seen not just as an extrapolation, but as a generalization—it is a proposal about something in common regarding the causal processes in the study situation and the human population of interest. As a generalization, it ought to apply to other situations as well—or at least have reasons why it does not—and one can evaluate the success of the hypothesis at being in accord with the whole suite of relevant observations at hand. If there are limits to the generalization—it applies to one species but not another, to males but not females, at this dose but not that dose—then the plausibility of such exceptions in view of available evidence and broader knowledge becomes part of the evaluation of the hypothesis against available data. (Such inferences and evaluations are particularly susceptible to the kind of post hoc modification of hypotheses mentioned above, and care must be taken to account for after-the-fact adjustments of the hypothesis in evaluating its strength.)

#### 2.1.1. Hill Criteria and the concept of “accounts”

Whenever a causal hypothesis is proposed, there is always (at least implicitly) a counter-hypothesis that the common link does not exist, and the array of outcomes we see among the studies at hand have other explanations that do not bear the same implications about potential risk in human target populations. When evaluating hypotheses, we suggest that it is important to make these counter-hypotheses explicit as well, including as much specificity about the nature of these “other explanations” as can usefully be provided, so that the alternatives can also be evaluated against all the data. In the end, compelling hypotheses are ones that not only are in accord with and serve to explain patterns and concordances among the data, but also have few ad hoc adjustments to account for observations that do not fit; moreover, they provide markedly more plausible explanations of the array of results on hand than can be provided by the counter-hypotheses. Evaluating explicit hypotheses and their alternatives against all the data provides transparency about the basis for expert professional judgment and communicates how scientifically compelling alternative explanations, with different consequences for human risk potential, ought to be deemed.

The question of evaluating causality in epidemiologi-cal data is often approached by applying the so-called “Hill Criteria” developed by Sir Austen Bradford Hill (Hill, 1965). A similar or “extended Hill-Criteria” approach has often been applied beyond the realm of epidemiology. In view of this established practice, the question may arise: What does HBWoE provide that is not already provided by the Hill Criteria? First, one should note that the Hill Criteria were developed for application to epidemiology data, which by nature are more observational than experimental. The criteria relate to the patterns among observational studies that one ought to expect if a common causal effect were operating but, independently, do not demonstrate causation. At most, adherence of data to the criteria constrains the scope for alternative, non-causal explanations. Epidemiology rarely has the ability to put causal explanations to the test (other than by evaluating consistency with further studies), and the kind of critical tests that can be constructed in experimental studies, with alternative influential factors controlled, is rarely available. Our goal of furthering the integration of epidemiological and toxicological inference is aided by an approach that gives experimentation, and the kind of critical tests that it can provide, a central role.

Second, as often applied, the Criteria become something of a checklist or a set of headings for citation of outcomes favorable or opposed to a causal hypothesis, but each evaluation is often not done very rigorously or transparently and suffers from the criticism we mentioned above—simply citing the studies that fit and announcing a professional judgment conclusion. Hypothesis-based weight of evidence can be seen as a process for encouraging rigorous and transparent evaluation of the criteria, particularly those referring to consistency, specificity, repeatability, and biological plausibility. In keeping with the theme of not simply making judgments, but rather showing the proposed basis for those judgments, HBWoE emphasizes not just the conclusions ab out each criterion, but also a transparent and articulated examination of its logical and evidentiary basis. To rigorously address the question of biological plausibility, one needs to follow a method similar to what we propose.

Finally, as Bradford Hill originally intended, his criteria (which he called “postulates”) were designed to articulate the basis for judgments and facilitate the integration of evaluations across criteria, not simply as a checklist for which, if enough features of the array of data seemed to fit, causality could be concluded. Hill saw the postulates as guides to thinking rather than as measures of evidence. In our reading of Hill's original paper, his intent for the application is along precisely the lines we propose—the evaluation of a specific causal hypothesis against alternative non-causal explanations. Bradford Hill makes explicit the importance of considering alternative “accounts” of the observations at hand in stating:

None of my nine viewpoints can bring indisputable evidence for or against the cause-and-effect hypothesis and none can be required as a sine qua non. What they can do, with greater or less strength, is to help us to make up our minds on *the fundamental question*—*is there any other way of explaining the set of facts before us, is there any other answer equally, or more, likely than cause and effect?* (Hill, 1965) [emphasis added]

The essence of the “accounts” (which we put forth in this context as a technical term) is that they constitute being explicit about Bradford Hill's “ways of explaining the set of facts before us.” They are not conclusions or findings but, rather, provisional proposals for the reasons behind the set of observations at hand.

Hypothesis-based weight of evidence comes down to evaluation of alternative accounts. An account is a set of proposed explanations and hypotheses that could be put forth to explain all of the observed data at hand. The array of all observations among all relevant studies comprises the fixed set of available facts; the challenge of scientific investigation is to discern what causes and processes account for those facts having come out as they did. Among the explanations that could be tentatively proposed are causal underlying processes that, if true, would lead to observed patterns and apparent connections within and among studies, but one could also entertain explanations that attribute particular outcomes to chance fluctuations, biases in measurement or reporting, confounding factors, operation of case-specific influences of unknown nature, or other such reasons. In the end, all the facts have to be accounted for by some combination of these, since the study outcomes came out as they did for some reason, even if we do not have clear ideas of what those reasons are. Any one proposed set of such reasons constitutes an account—a tentative “story” as to why the facts are as they are.

Clearly, there could be an infinite set of different accounts, but, in practice, there will be a few major contenders. Since the purpose of the weight-of-evidence evaluation is to identify underlying causal factors of relevance to our larger question, the key account will be one that proposes such an underlying causal factor. Such an account is centered on the proposed ability of a chemical to cause and increase the frequency of appearance of a particular toxic effect, put forward as a reason behind the existence of much of the apparent patterns and connections within and among studies. But there may be some facts on hand that are not readily attributed to such a factor, either ones that appear to contradict the general operation of the hypothesized cause or ones that, although not overtly contradicting, nonetheless are not explained by the key causal hypothesis. These facts need tentative explanations as well, from which subsidiary explanations also become part of the account.

There is always an important second account—one that denies the existence of the key causal factor and instead attributes the facts that appear to be explained by such a factor to other causes, either an alternative causal principle or simply a set of case-specific reasons under which any appearance of patterns within and across studies is mere happenstance. When one doubts the outcomes of a poor-quality study, one is in effect entertaining the possibility that some array of other factors or reasons (beside the one the study aimed at characterizing) has accounted for the outcomes, and the study's design does not allow one to attribute the outcomes confidently to the nominally tested influence.

When the “causal” account's plausibility overwhelms the alternative's, which by comparison seems to lack non-arbitrary reasons to deny the apparent patterns of causation, then we can feel confident that we have characterized a truly causal factor. But we undertake weight-of-evidence evaluations precisely when the case is not so clear—when the causal account itself has many facts that require modification or assumed special conditions of the causal hypothesis, or when there are apparently refuting facts that must be explained away as potential counterexamples. In short, weight of evidence is applied when the data at hand have contradictions and limitations such that even the optimal account requires ad hoc elements and assumptions to account for at least some of the problematic facts. The weight of evidence for the existence of the key causal factor consists of the comparative plausibility of the alternative accounts—the one that invokes it and the one that denies it. The credence we should give to an account and its implications for human health risk assessment depends on the degree to which it provides a more satisfactory and plausible accounting of the array of observations at hand than do any competing accounts. That is, we see the metaphor of “weight” of evidence as being evaluated with a two-pan balance—the relative plausibility of competing accounts—rather than as a single scale showing how much evidence in accord with a conclusion can be accumulated. Our approach to revealing and characterizing the plausibility of each account is to “unpack” the set of explanations they invoke, noting how much each strains credulity in view of the data at hand and wider knowledge of the relevant science. The explanations in each account need not be proven—what is important is that one set out the following questions:

- What is being proposed as causal and generalizable phenomena (i.e., what constitutes the basis for applying observations of biological perturbations or realized risks in other contexts to project potential risks to humans as they are exposed)?
- What is being proposed as the basis for deviations that lead to observations that do not fit the hypoth esized causal model (i.e., that would otherwise be counterexamples or refutations)?
- What assumptions are made that are ad hoc (i.e., to explain particulars, but for which the evidence consists of their plausibility and the observations they are adduced to explain)?
- What further auxiliary assumptions have to be made, and how reasonable are they in view of our wider knowledge and understanding?
- What is relegated to error, happenstance, or other causes not relevant to the question at hand?
- For those events or processes proposed as critical for a given account, what other observable manifestations should they have? Are these other manifestations indeed found?
- If either the operation or necessity of the proposed critical events for a given account were disproven, how else would one explain the array of outcomes?

### 2.2. HBWoE methodology

Although HBWoE is intended to be flexible in its application, the approach generally consists of the following steps, which are not intended to be a checklist and may involve an approach that is not necessarily in this order.

- Systematically review all studies that are potentially relevant to the causal question at hand (i.e., epidemiology, mode of action, pharmacokinetic, toxicology) and summarize the results without regard to whether they tend to support or undermine particular inter pretations. All potentially relevant data and modes of analysis, not only those featured or noted as significant by the studies' authors, should be included. The aim is to specify the set of relevant observations that can be brought to bear. Ask further questions about the data within these studies—specifically, think about the quality of the individual studies (strengths and weaknesses of study design, potential for ambiguity of interpretation of outcomes). Note the interpretation of data by the authors and how well those conclusions are supported by the reported observations. Note instances where evidence of associations depends on choosing the most significant among a set of parallel analyses of the same data (e.g., with different category cut-offs or different dose measures) and note whether there is any a priori reason to favor one mode of analysis over others. Note instances where the interpretation of proposed causes may have been accommodated to account for patterns in the data after the fact (e.g., preferring one dose measure over another because it provides a more interpretable pattern to dose-response data). The aim is to provide the basis for a critical review of the available studies, rather than simply collecting the findings noted and conclusions drawn by study authors.
- Within a realm of investigation (e.g., epidemiology, animal toxicology studies), examine the data for particular endpoints across studies. The aim is to evaluate consistency, specificity of apparent effects, and repeatability of outcomes. Note instances of similar patterns across studies, species, sexes, strains, etc., and also instances of apparent discordance among these. The aim is to provide the basis for judging the apparent limitations or exceptions to proposals about generally operating causal effects.
- Identify and articulate lines of argument by which results from available studies could be used to infer the existence, nature, or magnitude of human risk. These could be newly proposed or they could be proposals already put forth within the scientific community that one seeks to evaluate. Each line of argument should specify the data on which the inference would be based and also the reasoning for why those data are informative about the human risk question. Typically, the reasoning would entail a generalization about causal forces such that some commonality is proposed between the causal forces seen in the study data and those that would be presumed to operate in the human target population. It is important to specify how widely the invoked commonality is proposed to apply (e.g., just to humans but not experimental animals, or just to one sex, or just to humans and a particular strain of animals). The proposed reasons for why the limits to generalization exist should also be specified, to the degree possible (so one can evaluate whether they have an evidentiary basis or are simply ad hoc). These lines of argument are the “hypotheses” of HBWoE, and they are articulated so that one can evaluate how well they are in agreement with all of the data, how well they would explain patterns in the data if they were true, what other observable consequences the invoked causal principles should have, and whether in fact these consequences are observed.
- Trace through the logic within each line of evidence. That is, think about how all of the relevant studies within each line of evidence support each other, considering consistencies and inconsistencies across studies. For example, one would do this for all of the epidemiology studies together (i.e., apply Bradford Hill Criteria), all of the mode-of-action and pharma-cokinetic data together, and all of the toxicology data together. The aim is to establish how well the hypotheses being examined comport with and help explain common patterns in the data, what data seem to constitute exceptions or contrary outcomes to the hypothesized causal principles, and what reasons for such exceptions might be proposed.
- Trace through the logic regarding all lines of evidence as a whole and how they inform interpretation of each other. Specifically, how the epidemiology studies as a whole, mode-of-action studies as a whole, and toxicology data as a whole (that we have articulated as part of Step 4) inform interpretation of one another. The question is whether explanations or hypothesized causal factors proposed in one realm (e.g., epidemiology) have aspects that should be observable in others (e.g., mode-of-action studies), enabling evaluation of whether signs of those causal processes do or do not appear where expected.
- Next, one needs to formulate alternative accounts. Each account comprises a set of proposals, hypotheses, assertions, and assumptions that together should provide a tentative story for why all of the relevant observations came out as they did. Each of the causal hypotheses identified in Step 5 would constitute the core of an account, but the same account should also include the proposed reasons why facts that do not fit or are deemed to be outside the span of generalization should not be taken as disproofs because their non-concordance is explicable. An account that denies a central causal hypothesis as an explanation for an apparent association needs to provide an alternative proposed explanation for the observed patterns.
- Finally, evaluate alternative, and competing, accounts. Now that one has worked carefully through not only each study and each individual line of evidence but, importantly, considered how each line of evidence informs the other, it is at this point that one asks how well each hypothesis is supported by the data and how many ad hoc assumptions are required to support each hypothesis. The rationale and reasoning for how the data support (or do not support) each account's hypotheses, together with the plausibility of subsidiary explanations or assumptions in view of wider biological knowledge, constitute the basis for evaluating the scientific support each account gets from available data. The comparative support constitutes the basis for judging the relative credence that alternative accounts should be given.
- The goal in the end is to present the lines of reasoning for (not to prove or disprove) each account, based on the science and integration of the lines of evidence, so that the data will speak for themselves in support ing (or not supporting) the overarching hypotheses that have been put forth.
- By comparison of the various accounts, one may be left with a variety of outcomes or proposed next steps. The results may suggest sharpening a proposed hypothesis, or there maybe obvious data gaps that can now be pursued more clearly so that each account can be defined more clearly, or one account maybe more clearly supported by the data than other accounts. An advantage of the HBWoE approach is that it can help identify research that would be most able to inform outstanding questions and resolve ambiguous interpretations.

In this article, we first describe an overview of the HBWoE evaluation of formaldehyde and leukemogenesis by describing the various accounts that must be considered before concluding whether a possible causal association exists between formaldehyde exposure and leukemogenesis. We then describe the details of our analysis for each of the lines of evidence (epidemiology, toxicology, pharmacokinetic, and mode of action) that form the bases of these accounts, individually and in terms of how each inform each other.

## 3. Overview of HBWoE as applied to formaldehyde and leukemogenesis

The HBWoE evaluation for human leukemogenesis from inhaled formaldehyde comes down to evaluating the comparative degree to which each of the alternative accounts is supported by reference to scientific evidence. In short, one is faced with a contradiction between the apparent (though not certainly causal) association of leukemia with formaldehyde exposure in at least some human studies and the apparent implausibility of such a causal effect in view of current biological understanding. The apparent contradiction can be reconciled in one of two ways: (1) by accepting that human risks are actually increased and positing that the biological impossibility of such increases is somehow mistaken—that is, since the effect appears, it must have a possible causal explanation; or (2) by concluding that doubts about possible mechanisms have merit, and the apparent association of formaldehyde and leukemia seen in some human studies does not in fact indicate a causal connection (and that those studies showing lack of effect are indeed the ones to be taken at face value)—that is, the appearance of some apparent associations is in fact accounted for by chance or by shortcomings in the ostensibly positive human studies, which, according to this view, should be deemed false-positive results.

In pursuit of the first account that suggests a causal mechanism must exist between formaldehyde exposure and leukemia because their effects are seen, several candidate causal mechanisms have been hypothesized (Zhang et al., 2009, 2010a). As these mechanisms are evaluated, it is important to consider their ad hoc nature; rather than being suggested a priori because of plausibly relevant observed properties, they are constructed after the fact specifically to propose a remedy to the fatal shortcoming of impossibility. Furthermore, they are constrained by the need to offer a possible causal connection between leukemia and formaldehyde inhalation without producing observable effects that contradict currently accepted knowledge and observations. This ad hoc nature does not make the hypothesized mechanisms false, but it does put a premium on finding some independent, positive evidence of their operation and role rather than simply relying on their ability, if true, to furnish the needed mechanisms or apparent consistencies with observations, since they were chosen in part as support of these observations and proposed mechanisms.

An alternative, and contrasting, account is that it is not possible for formaldehyde to move beyond the nasal respiratory mucosa to cause systemic DNA damage and cellular transformation (in the bone marrow, circulating hematopoietic stem cells, or the NALT), and therefore there is no biologically plausible mechanism for formaldehyde leukemogenesis. This account is supported by a large body of hematotoxicity studies (in animals and humans); toxicokinetic, genotoxocity, and mechanistic data in animals, humans, and in vitro; and a large body of null epidemiology findings. Under this account, the significant number of null epidemiology findings are considered true results, and the few positive findings in the epidemiology studies (which have shortcomings, including poor disease classification and poor estimates of exposure), are likely attributable to confounding by other exposures or to chance. If this account is true, an association between inhalation of formaldehyde and leukemia would be understood as not plausible for humans.

Our HBWoE evaluation compares these two accounts by first describing what is known and what has been interpreted from the formaldehyde epidemiology, toxicology, and mode-of-action data, pointing out questions that arise from within and across these studies and their interpretation, the answers to (or at least discussions of) which provide the bases for tracing the logic for each alternative hypothesis.

## 4. Weight of epidemiology evidence regarding the association between formaldehyde exposure and leukemia

To conduct the HBWoE analysis of the epidemiology data regarding the association between formaldehyde exposure and leukemia, we first conducted a literature search, using PubMed and TOXLINE, for all human studies measuring or estimating formaldehyde exposure and the incidence of or mortality from any lymphohe-matopoietic cancer. Search terms included “leukemia,” “lymphoma,” “Hodgkin,” “non-Hodgkin,” “hematologic neoplasm,” “myeloma,” “hematopoietic,” “lymphatic,” “formaldehyde,” “epidemiol*,” “occupation*,” “cohort*,” and “worker*.” We also relied on the reference lists of several review articles and meta-analyses (e.g., Bachand et al., 2010; Zhang et al., 2010a; Bosetti et al., 2008; Collins and Lineker, 2004). We critically reviewed each relevant study and focused particularly on two cohorts that have received much recent attention: the National Cancer Institute (NCI) industrial worker and embalmer cohorts. The former was analyzed in several studies using traditional cohort study designs, whereas individuals were drawn from the latter to conduct case-control analyses.

After providing a brief overview of the epidemiology literature below, we describe an endpoint-by-endpoint analysis of each lymphohematopoietic cancer and groups of cancers that have been investigated. This is followed by an HBWoE evaluation of the epidemiology evidence with respect to the hypothesis that formaldehyde causes leukemia.

### 4.1. Overview of epidemiology investigations

Several cohort and case-control studies have been conducted on formaldehyde exposure and lymphohematopoietic cancers (Tables 1 and 2). The first study published was of pathologists and medical laboratory technicians in the United Kingdom (UK) who were followed through 1973 (Harrington and Shannon, 1975). Since that time, studies of embalmers, undertakers, funeral directors, radiologists, pathologists, anatomists, leather tannery workers, iron foundry workers, plastics manufacturing workers, wood industry workers, garment workers, pest-control workers, and workers at formaldehyde production or usage plants have been conducted in the United States, the UK, France, Sweden, Italy, Denmark, Finland, and Canada. Cohort studies ranged in size from 154 to 126,347 subjects with follow-up beginning as early as 1925 and up through 2004. Among the eight case-control studies we identified, the largest included 1511 cases, and follow-up periods among the studies ranged from 1940 to 2000 (Table 2). Formaldehyde exposure was rarely measured in any study and, when it was, concentration information was not available for the entire period of employment. Owing to the limited concentration data, exposure was typically estimated based on job descriptions. Formaldehyde risks were then calculated based on the date of hire/first exposure, minimum employment duration, duration of employment/exposure, time since first exposure, cumulative exposure, average exposure, average intensity of exposure, peak exposure, and number of peak exposures. Health outcomes were coded according to the International Classification of Diseases (ICD) 7th, 8th, or 9th revision (Table 3). Because the majority were coded using the 8th revision (ICD-8) and there are few differences between the 8th and 9th revisions, classifications in the following sections and the tables refer to the 8th revision unless otherwise noted. The health outcomes assessed included mortality from lymphohematopoietic cancer (ICD 200-209), cancer of lymphoid origin (ICD 200-204), leukemia (ICD 204-207), hematopoietic cancer of non-lymphoid origin (ICD 205, 206, 208, 209), lymphatic leukemia (ICD 204), myeloid leukemia (ICD 205), other unspecified leukemia (ICD 207), Hodgkin's lymphoma (ICD 201), non-Hodgkin's lymphoma (ICD 200, 202), and multiple myeloma (ICD 203). The majority of studies were subject to confounding by several co-exposures, many of which were not accounted for in statistical analyses.

**Table 1:** Formaldehyde cohort studies

Reference	Study population	Subjects (n)	Job/Exposure Category	Period of Employment	Period of Follow-up	Total Follow-Up (person-years)	Minimum Employment (years)	Mean Time-Weighted Average Exposure (ppm)	Peak Exposure (ppm)	Cumulative Number of Peaks ≥4.0 ppm
Harrington and Shannon, 1975	UK Pathologists and medical laboratory technicians	156 154	Pathologists Medical laboratory technicians	1955–1973 1963–1973	1955–1973 1963–1973	24,119.7 73,025.6				
Walrath and Fraumeni, 1983	New York State embalmers	1,132	Embalmers (length of time from first license to death was used to approximate exposure)	1902–1980	1925–1980					
Wong et al., 1983	United States Formaldehyde plant workers	2,026	White male chemical workers	1940–1977	1940–1977	32,514.3				
Levine et al., 1984	Ontario, Canada undertakers	1,477	Undertakers exposed to formaldehyde	1928–1957	1950–1977	34,774				
Walrath and Fraumeni, 1984	California embalmers	1,007	Embalmers (length of time from first license to death was used to approximate exposure)	1916–1978	1925–1980					
Bertazzi et al., 1986, 1989	Italian male resin producers	1,332	Workers exposed to formaldehyde, exposed to other compounds or exposure unknown	1959–1980	1959–1986	5,731	≥1 month			
Logue et al., 1986	US radiologists and pathologists	785 455	Radiologists Pathologists (based on entrance into professional society)	1962–1977	1962–1977					
Stroup et al., 1986	US anatomists	2,317	Anatomists	1889–1969	1925–1979					
Edling et al., 1987	Swedish abrasive manufacturing workers	521	Abrasives industry workers	1958.1981	1958.1981		≥5			
Robinson et al., 1987	US plywood mill workers	2,283	Plywood mill workers	1945–1955	1945–1977	57,588	≥1			
Stern et al., 1987	Minnesota and Wisconsin leather tannery workers	9,365	Tannery A Tannery B Department (finishing 0.5–7 ppm formaldehyde)	1940–1979	1940–1982					
Matanoski et al., 1991	US pathologists	6,411	Pathologists	1912–1950	1925–1978					
Hayes et al., 1990	US embalmers and funeral directors	4,046	Embalmers and funeral directors exposed to formaldehyde (measured average 0.98–3.99 ppm and peak 20 ppm)	NR	1975–1985					
Hall et al., 1991	UK pathologists	3,872	Pathologists	1974–1987	1974–1987					
Andjelkovich et al., 1995	US iron foundry workers	3,929	Iron foundry workers (formaldehyde exposed or unexposed)	1960–1987	1960–1989	83,064	≥6 months	Low 0.05 Medium 0.55 High 1.5		
Dell and Teta, 1995	New Jersey workers at plastics manufacturing and R&D facility	5,932	Hourly and salaried employees	1946–1967	1946–1988		≥7 months			
Hansen and Olsen, 1995	Denmark formaldehyde male workers	126,347	Working for company making or importing formaldehyde at least 10 years before diagnosis	1970–1984	1970–1984					
Chiazze et al., 1997	South Carolina fiberglass workers	4,631	Cumulative exposure to formaldehyde	1951–1991	1951–1991	73,259				
Stellman et al., 1998	US wood industry workers	45,399	Woord workers Wood dust exposed workers (asbestos and formaldehyde exposure)	1982–1988	1982–1988	2,101,145				
Marsh et al., 2001	US fiberglass workers	32,110	Workers exposed to formaldehyde in ten fiberglass plants	1945–1978	1946–1992	209,726	≥1			
Coggon et al., 2003	UK factory workers where formaldehyde was used or produced	14,014	Formaldehyde production workers	1941–1989	1941–2000			<0.1			
								0.1–0.5		
								0.6–2.0		
								≥2.0		
Pinkerton et al., 2004	Georgia and Pennsylvania garment workers	11,039	Garment workers	1955–1982	1955–1998		≥3 months			
Ambroise et al., 2005	French pest-control workers	181	Pest-control workers (ever employed)	1979–2000	1979–2000	3107				
Beane Freeman et al., 2009 (update of Hauptmann et al., 2003)	US workers at formaldehyde production or usage plants	25,619	Formaldehyde production workers (exposed or unexposed)	1934–1966	1934–2004	998,106			0	Data not shown
									0.1–1.9	
									2.0.39≥4.0	
Harrington and Shannon, 1975							ICD-8, 200.209	8/3	4.0/5.5	
							201	1/01/1	0.7/1.6	
							204.207		1.6/2.2	
Walrath and Fraumeni, 1983					<35		ICD-8, 200–209	25	20.6	Embalming fluids that contain other chemicals (e.g., tissue moisturizers, antiseptic solutions, dyes, and deodorizers)
					≥35		200	5	4.7	
							201	2	2.3	
							202, 203	6	4.9	
							204.207	12	8.5	
Wong et al., 1983			<5		10	Before 1961	ICD-8, 200–209	6	4.42	Formaldehyde, oxygenated hydrocarbons, benzene, asbestos, pigments
			5–9		20	After 1961	201	2	0.83	
			10–14				204–207	2	1.70	
			15–19							
			20+							
Levine et al., 1984							ICD-8, 200–209	8	6.5	Methanol, phenol, and dyes
							204–207	4	2.5	
Walrath and Fraumeni, 1984			<20				ICD-8, 200–209	19	15.6	Embalming fluids containing coloring and modifying agents, anticoagulants, surfactants, deodorants, and vehicles
			≥20				200	3	3.1	
							201	0	2.5	
							202, 203, 208, 209	4	3.0	
							204–207	12	6.9	
Bertazzi et al., 1986, 1989							ICD-8, 200–209	3	1.11	Styrene, polystyrene
Logue et al., 1986						Before 1962	ICD-7, 200–203,	Not reported	Not reported	Radiation
						After 1962	205			
Stroup et al., 1986							ICD-8, 200–209	18	14.6	Solvents, methyl alcohol, phenol, and biological agents
							200	2	2.9	
							201	0	1.9	
							204–207	10	6.8	
							202–203, 208–209	6	3.0	
Edling et al., 1987						ICD-8, 200–202	2	1.0	Aluminum oxide, silicon carbide, clay, phenol, silica, total dust	
							203	2	0.5	
Robinson et al., 1987			<20		<20		ICD-7, 200	4	3.9	Wood dust, pentachlorophenol, carbon disulfide, and volatiles
			≥20		≥20		201	2	1.8	
							204	3	0.9	
							202, 205	3	1.1	
Stern et al., 1987			<1		≥15		ICD-7, 200–205	8/14	12.3/19.4	Cu, Cr, Mn, Co, n-butyl acetate, MEK, MIK, toluene, xylene, acetone, dust, and butyl cellosolve
			1–9				204	4/6	5.2/8.0	
			≥10			200–203, 205	4/8	7.0/11.4		
Matanoski et al., 1991							ICD-8, 200–209	115	82.7	Phenol, methyl alcohol, glutaraldehyde, and biologic materials, and in the past were exposed to mercury, arsenic, and zinc
							201204–207	3	4.2	
								34	27.1	
								12	10.7	
								20	14.6	
								22	16.3	
								7	9.4	
								24	15.3	
								20	8.8	
								3	0.8	
								4	2.6	
Hayes et al., 1990							ICD-8, 200–209	1104	0.83	
							201		6.93	
							200, 202	2.63		
							200			
							203			
							202			
							204			
							205			
							206, 207			
							208			
							209			
Hall et al., 1991							ICD-8, 201	57	45.6	Other chemicals and infectious agents
							200–209	2	5.6	
							204–207	31	23.0	
Andjelkovich et al., 1995							ICD-8, 200–209	7	12.0	Silica, PAHs, nickel, and chromium
							200	1	1.8	
							201	1	1.4	
							204–207	2	4.6	
Dell and Teta, 1995			<5		10		ICD-7, 200–205	23	13.63	Asbestos, carbon black, epichlorohydrin, formaldehyde, polyvinyl chloride (PVC), acrylonitrile, styrene, and numerous chemical additives, such, as plasticizers, emulsifiers, and antioxidants
			5–9		10		200	3	2.39	
			10–19		15		204	11	5.56	
			≥20				204.4	8	4.30	
Hansen and Olsen, 1995							ICD-7, 200, 202	32	27.2	Wood dust, other chemicals
							201	12	12.2	
							204	39	47.0	
Chiazze et al., 1997							ICD-7, 200–205	51	10.8	Respirable glass fibers, total particulate, asbestos, refractory ceramic fibers, respirable silica, total chrome, and arsenic
							204		54.11	
Stellman et al., 1998			<10				ICD-9, 200–208	28	NR	Wood dust and asbestos
			10–19				200, 202	11		
			≥20				203	4		
							204–208	12		
Marsh et al., 2001							ICD-8, 200–209	199	NR	Fiberglass fibers, arsenic, asbestos, asphalt, epoxy, phenolics, silica, styrene, and urea
Coggon et al., 2003			<1				ICD-9, 201 200,	6	8.5	Asbestos, styrene, ethylene oxide, epichlorhydrin, solvents, chromium, and cadmium
			1–14				202,	31	31.7	
			≥15				202.1, 202.8	15	17.5	
							203 204–208	31	34.1	
Pinkerton et al., 2004			<3	<1010–19≥20		<19631963–1970≥1971	ICD-9, 200–208	59	60.8	
			3–9				200	5	5.9	
							201	2	3.6	
							204–208	24	22.0	
							202–203	28	28.9	
Ambroise et al., 2005		Four quartiles of exposure					ICD-9, 204–208	1	0.23	Ethylene oxide, insecticides, and rodenticides (over 60 chemicals)
Beane Freeman et al., 2009 (update of Hauptmann et al., 2003)	0	0	Data not shown	0	0		ICD-8, 200–209	286	304.3	Antioxidants, asbestos, benzene, carbon black, dyesand pigments, hexamethylenetetramine, melamine, phenol,plasticizers, urea, and wood dust
	>0–<0.5	>0–<1.5		>0–15	>0–25		200, 202 201 203	94	110.6	
	0.5–<1.0	1.5–<5.5		>15–25 >25–35	>25–42		204–207	25	17.6	
	≥1.0	≥5.5		>35	>42		204	48	51.1	
							205	116	113.7	
								36	31.3	
								44	48.9	

Note: NR = not reported. See Table 3 for ICD codes.

**Table 2:** Formaldehyde case control studies

Reference	Study Population	Job/Exposure Category	Period of Employment	Period of Follow-up	Total Follow-up (person-years)	Minimum Employment (years)	Mean Time-Weighted Average Exposure (ppm)	Peak Exposure (ppm)	Average Intensity (ppm)
Gerin et al., 1989	Canadian population in Montreal	Lifetime job histories obtained by interview and translated into level of exposureto formaldehyde	1979–1985	1979–1985					Low
									Medium
									High
Ott et al., 1989	US Union Carbide chemical manufacturing facilities	111 work areas, 21 specific chemicals and 52 chemical-activity groups	1940–1978	1940–1978		≥1 day			
Linos et al., 1990	Iowa and Minnesota Funeral home workers	Funeral service and crematoria workers	NR	NR					
Partanen et al., 1993	Finland Wood production workers	Wood workers (formaldehyde, solvents, wood dust)	1957–1982	1957–1982		≥1			
Tatham et al., 1997	Atlanta, Connecticut, Iowa, Kansas, Miami, San Francisco, Detroit, and Seattle workers	Exposed to formaldehyde or other chemicals	1984–1988	1984–1988	≥1				
Blair et al., 2001	Iowa and Minnesota Industrial workers	15 different industrial and occupational job categories (non-farming)	1980–1983	1980–1983	≥1				Wang et al., 200b
Hauptmann et al., 2009	US Embalmers	Never Embalming Ever Embalming	1932–1986	1960–1986	19,104	0	0	0	
							>0–0.10	>0–7.0	>0–1.4
							>0.10–0.18	>7.0–9.3	>1.4–1.9
							>0.18	>9.3	>1.9

Reference	Cumulative Exposure (ppm)	Duration of Exposure or Length of Employment (years)	Number of Embalmings	ICD Code	Cases (total)	Controls (total)	Possible Co-Exposures Discussed
Gerin et al., 1989		<10		ICD-8, 200, 202	206	533			
		≥10		201	53				
Ott et al., 1989		0		Non-Hodgkin's	2	NR	52 chemical groups (e.g., epoxides, halogenated compounds, fused cyclics, nitriles, vinylics)		
		<5		Multiple	1				
		≥5		Myelpoma	3				
				Leukema					
Linos et al., 1990				Leukemia	578	1245	NR		
				Non-Hodgkin's	622				
Partanen et al., 1993				ICD-7, 200–202	5	152	Wood dust, pesticides, chlorophenols, phenol, terpenes, solvents (stains, lacquers, toluene, xylenes, benzene, styrene, butyl acetate, ethyl acetate, butanol, isopropanol, ethanol), aliphatic hydrocarbons (solvent naphtha, white spirits), ketones, glycol ethers, and engine exhaust		
Tatham et al., 1997		<10		ICD-8, 200, 202	1511	1659	Pesticides, herbicides, wood/saw dust, solvents, shoe/leather dust, meat packaging or processing, metal plating, cutting oils, chlorophenols, heterocyclic nitrogens, carbamates, organophosphates, phenoxy herbicides, chlorinated hydrocarbons, and pyrethroids		
Blair et al., 2001	Low	<10		ICD-8, 204–207	64	137	Solvents, paints, metals, solder		
	High	≥10		205.0	14				
				205.1	8				
				204.0	0				
				204.1	30				
Wang et al., 200b	Never			ICD-9, 200–202	601	717	Organic solvents (benzene, chloroform, carbon tetrachloride, dichloromethane, methyl chloride, trichloroethylene)		
	Low								
	Medium–High								
Hauptmann et al., 2009	0	0	0	ICD-8, 200–209	168	265	Isopropanol, ethylene glycol, methanol, phenol, glutaraldehyde, ionizing radiation, benzene, and cigarette smoking		
	>0–4058	>0–20	>0–1422	200–204	99				
	>4058–9253	>20–34	>1422–3068	205, 206, 208, 209	48				
	>9253(ppm-h)	>34	>3068	205	34				

Note: NR = not reported; AML = acute myeloid leukemia; CML = chronic myeloid leukemia; ALL = acute lymphoid leukemia; CLL = chronic lymphoid leukemia. See Table 3 for ICD codes.

**Table 3:** International disease classification (ICD) codes

ICD Code	Revision 7	Revision 8	Revision 9
	(200–207) Neoplasms of lymphatic and hematopoietic tissues	(200–209) Neoplasms of lymphatic and hematopoietic tissue	(200–208) Malignant neoplasms of lymphatic and hematopoietic tissue
200	Lymphosarcoma and reticulosarcoma	Lymphosarcoma and reticulum-cell sarcoma	Lymphosarcoma and reticulosarcoma and other specified malignant tumors of lymphatic tissue
201	Hodgkin's disease	Hodgkin's disease	Hodgkin's disease
202	Other forms of lymphoma (reticulosis)	Other neoplasms of lymphoid tissue	Other malignant neoplasms of lymphoid and histiocytic tissue
203	Multiple myeloma	Multiple myeloma	Multiple myeloma and immunoproliferative neoplasms
204	Leukemia & aleukemia	Lymphatic leukemia	Lymphoid leukemia
204.0	Lymphatic leukemia	Acute lymphocytic leukemia	Acute lymphoid leukemia
204.1	Myeloid leukemia	Chronic lymphocytic leukemia	Chronic lymphoid leukemia
204.3	Acute leukemia	—	—
204.4	Other & unspecified leukemia	—	—
205	Mycosis fungoides	Myeloid leukemia	Myeloid leukemia
205.0	—	Acute myeloid leukemia	Acute myeloid leukemia
205.1	—	Chronic myeloid leukemia	Chronic myeloid leukemia
206	Lymphatic system	Monocytic leukemia	Monocytic leukemia
207	Hematopoietic system	Other and unspecified leukemia	Other specified leukemia
208	—	Polycythemia vera	Leukemia of unspecified cell type
209	—	Myelofibrosis	—
238.4	—	— Polycythemia vera	
289.83	—	— Myelofibrosis	
294	Polycythemia	—	—

Several individuals and/or cohorts were analyzed in more than one study. Beane Freeman et al. (2009) conducted the most recent study of the NCI industrial worker cohort, with follow-up through 2004. This cohort was first studied by Blair et al. (1986), who followed workers employed in 10 formaldehyde-producing or -using facilities through 1979. Hauptmann et al. (2003) conducted a follow-up through 1994, although it was noted by Beane Freeman et al. (2009) that 1006 deaths were omitted unintentionally from this analyses (all results presented here are from a reanalysis by Beane Freeman et al. [2009], which included these deaths). To avoid counting information on this cohort more than once, only data from the most recent publication by Beane Freeman et al. (2009) are shown in the tables, but results from the previous studies of this cohort are discussed in the text if they are not consistent with the latest analysis.

Coggon et al. (2003) evaluated a cohort of 14,014 UK workers at factories where formaldehyde was used or produced that had been evaluated previously by Acheson et al. (1984) and Gardener et al. (1993). Acheson et al. (1984) evaluated mortality in 7680 men first employed before 1965 in one of six factories, with follow-up through 1981. Gardener et al. (1993) extended the follow-up of 7660 of these workers through 1989, and began following 6357 additional workers who began work after 1964. Coggon et al. (2003) then followed the majority of these workers through 2000. Because results are consistent among the three analyses, only results from Coggon et al. (2003) are discussed here.

Hauptmann et al. (2009) conducted a case-control study based on over 6000 embalmers (NCI embalm-ers cohort) who died between 1960 and 1985 and were included in proportionate mortality ratio (PMR) studies by Hayes et al. (1990) and Walrath and Fraumeni (1983, 1984). Walrath and Fraumeni (1983) studied embalmers licensed in California, Walrath and Fraumeni (1984) studied those licensed in New York, and Hayes et al. (1990) assembled data on US embalmers and funeral directors who died between 1975 and 1985. In the tables, we present data from both Hauptmann et al. (2009) and Hayes et al. (1990) because they use different methodologies. Data from Walrath and Fraumeni (1983, 1984) are discussed in the text but not the tables, because study subjects are included in the Hayes et al. (1990) analysis and were analyzed in a similar fashion.

### 4.2. Endpoint-by-endpoint analysis

In this section, we discuss each of the individual lympho-hematopoietic cancer endpoints analyzed in the epidemiology studies described above. Lymphohematopoietic cancers include a group of hematopoietic and lymphoid cell disorders that have distinct classifications based on morphologic, cytogenic, immunophenotypic, and molecular characteristics (see Vardiman, 2010, for a review of the classifications). We consider various groupings of cancer types as analyzed by study authors, although results from these analyses must be considered carefully because each specific lymphohematopoietic cancer is a different disease. Although some cancer types may have some common mechanisms (e.g., pharmacokinetics), in general, lymphohematopoietic cancers each have a distinct etiology, so an association with one type is not necessarily indicative of risk of another (Schottenfeld and Fraumeni, 2006). That is, if one study reports a statistically significant finding for one cancer type (A) but not another (B), and another study reports a statistically significant finding for cancer type B but not A, this is not consistent evidence of an association. In the same vein, an association between formaldehyde and a group of cancers does not necessarily provide evidence for all cancers in that group, as it maybe driven by one cancer type with a distinct mode of action. Thus, it is crucial in a weight-of-evidence analysis to consider each individual cancer type and the implications of analyses of cancer groups.

For each cancer or group of cancers, we evaluated the weight of each study based on several factors, including the study objectives and hypothesis; the study subjects; the exposure and health outcome assessments; the follow-up period; the consideration of bias, confounders, and effect modifiers; the statistical methods; the documentation and interpretation of results; and the external validity (i.e., the bearing on the larger question at hand, formaldehyde as a potential cause of human lymphohematopoietic neoplasms). For each cancer or group of cancers, we also assessed the consistency of findings (which included consideration of the type of exposure metric, e.g., peak vs. cumulative) and whether any exposure-response relationships were evident.

#### 4.2.1. All lymphohematopoietic cancers

The association between formaldehyde exposure and all lymphohematopoietic cancers combined has been investigated in 12 studies (Table 4). Eleven cohort and one case-control study assessed whether study subjects had an increased risk over the general population. Of these, only one reported associations (Hayes et al., 1990). Hayes et al. (1990) found an increased proportion of deaths attributable to lymphohematopoietic cancers among embalmers in the NCI embalmers cohort (PMR=1.39, 95% confidence interval [CI]: 1.15-1.67).

**Table 4a:** Association between formaldehyde and all lymphohematopoietic cancers (ICD 200–209)

	Hauptmann et al., 2009					Beane Freeman et al., 2009					Andjelkovich et al., 1995				
			
	Embalmers case-control					NCI cohort (1934–2004)					Iron foundry workers				
			
Measures	Category	Obs	Estimate		95% CI	Category	Obs	Estimate		95% CI	Category	Obs	Estimate		95% CI
Unexposed/Exposed	Never embalming	24	OR	1.00	—	Unexposed	33	SMR	0.86	0.61–1.21	Unexposed	8	SMR	0.89	0.38–1.76
	Ever embalming	144	OR	1.40	0.80–2.60	Exposed	286	SMR	0.94	0.84.1.06	Exposed	7	SMR	0.59	0.23–1.21
Peak Exposure	0 ppm	24	OR	1.00	—	0 ppm	33	RR	1.07	0.70–1.62					
	>0–<2.0 ppm	48	OR	1.60	0.80–3.20	>0–<2.0 ppm	103	RR	1.00	—					
	2.0–<4.0 ppm	55	OR	1.60	0.90–3.10	2.0–<4.0 ppm	75	RR	1.17	0.86–1.59					
	≥4.0 ppm	41	OR	1.20	0.60–2.30	≥4.0 ppm	108	RR	1.37	1.03–1.81					
	*p*_trend_ = .302 (exposed)					*p*_trend_ = .02 (exposed)									
	*p*_trend_ = .555 (exposed and unexposed)					*p*_trend_ = .04 (exposed and unexposed)									
Average Intensity	0 ppm	24	OR	1.00	—	0 ppm	33	RR	0.99	0.66–1.48					
	0.1–0.4 ppm	53	OR	1.60	0.90–3.20	>0–<0.5 ppm	164	RR	1.00	–					
	0.5–0.9 ppm	47	OR	1.40	0.70–2.70	0.5–<1.0 ppm	67	RR	1.29	0.97–1.73					
	≥1.0 ppm	44	OR	1.30	0.70–2.50	≥1.0 ppm	55	RR	1.07	0.78–1.47					
	*p*_trend_ = .443 (exposed)					*p*_trend_ > .5 (exposed)									
	*p*_trend_ = .591 (exposed and unexposed)					*p*_trend_ > .5 (exposed and unexposed)									
Cumulative Exposure	0 ppm-yr	24	OR	1.00	—	0 ppm-yr	33	RR	0.89	0.59.1.34					
	>0–<1.5 ppm-yr	40	OR	1.30	0.60–2.50	>0–<1.5 ppm-yr	168	RR	1.00	—					
	>1.5–<5.5 ppm-yr	49	OR	1.40	0.80–2.80	>1.5–<5.5 ppm-yr	49	RR	0.77	0.56–1.07					
	≥5.5 ppm-yr	55	OR	1.60	0.80–3.00	≥5.5 ppm-yr	69	RR	1.07	0.8–1.42					
	*p*_trend_ = .753 (exposed)					*p*_trend_ = .25 (exposed)									
	*p*_trend_ = .422 (exposed and unexposed)					*p*_trend_ = .25 (exposed and unexposed)									
Cumulative number of peaks ≥4.0 ppm				No association. Results not shown.											
Exposure/Employment Duration	0 yrs	24	OR	1.00	—	No association. Results not shown.									
	>0–20 yrs	28	OR	0.80	0.40–1.80										
	>20–34 yrs	50	OR	1.50	0.80–2.80										
	>34 yrs	66	OR	1.80	1.00–3.40										
	*p*_trend_ = .131 (exposed)														
	*p*_trend_ = .058 (exposed and unexposed)														
Number of Embalmings	0	24	OR	1.00	—										
	>0–1422	29	OR	0.90	0.60–1.80										
	>1422–3068	62	OR	1.90	1.00–3.60										
	>3068	53	OR	1.50	0.80–2.90										
	*p*_trend_ = .477 (exposed)														
	*p*_trend_ = .844 (exposed and unexposed)														
8-Hour Time-Weighted Average Intensity	0	24	OR	1.00	—										
	>0–0.10	47	OR	1.30	0.70–2.60										
	>0.10–0.18	52	OR	1.60	0.80–3.10										
	>0.18	45	OR	1.40	0.70–2.80										
	*p*_trend_ = .635 (exposed)														
	*p*_trend_ = .855 (exposed and unexposed)														
Time Since First Exposure						0 yrs	30	RR	0.67	0.31–1.46					
						>0–15 yrs	21	RR	1.00	–						
						>15–25 yrs	46	RR	1.30	0.68–2.49						
						>25–35 yrs	59	RR	0.82	0.40–1.70						
						>35 yrs	163	RR	0.67	0.32–1.41						
Time Since First Exposure ≥4 ppm						0 yrs	211	RR	0.57	0.36–0.88					
						>0–25 yrs	28	RR	1.00	–					
						>25–42 yrs	45	RR	0.69	0.41–1.17					
						>42 yrs	35	RR	0.61	0.34–1.09					

**Table 4b:** Other cohorts

Reference	Obs	Estimate	95% CI	
Wong et al., 1983	6	SMR	1.36	0.50–2.95
Levine et al., 1984	8	SMR	1.24	—
Hayes et al., 1990	115	PMR	1.39	1.15–1.67
Hall et al., 1991	9 (M)	SMR	1.42	0.65–2.69
Hall et al., 1991	1 (F)	SMR	1.75	0.04–9.77
Matanoski et al., 1991	57	SMR	1.25	0.95–1.62
Bertazzi et al., 1986, 1989	3	SMR	1.73	0.36–5.06
Stellman et al., 1998*	28	RR	1.22	0.84–1.77
Marsh et al., 2001	199	SMR	0.90	0.78–1.04
Pinkerton et al., 2004*	59	SMR	0.97	0.74–1.26

* ICD-8 200–208.

Lymphohematopoietic cancer risks were also evaluated based on one or more exposure metrics in iron foundry workers, embalmers, and industrial workers. Risks were not increased in formaldehyde-exposed and unexposed US iron foundry workers (Andjelkovich et al., 1995), and risks reported in embalmers and industrial workers were not consistent across exposure metrics (Hauptmann et al., 2009; Beane Freeman et al., 2009).

Hauptmann et al. (2009) conducted a case-control study of 168 embalmers (21 with leukemia) from the NCI embalmers cohort (evaluated by Hayes et al., 1990) and examined lymphohematopoietic cancer risks based on seven exposure metrics: exposed (ever/never embalmed), peak exposure, average intensity of exposure when embalming, 8-hour time-weighted average (TWA) exposure, cumulative exposure, exposure duration (years embalming), and number of embalmings. Exposure estimates were developed from a previous exposure-assessment experiment by Stewart et al. (1992). The investigators conducted trend tests for each exposure metric including and excluding unexposed individuals. There were no statistically significant associations between formaldehyde exposure and lymphohematopoietic cancer based on any exposure metric.

Beane Freeman et al. (2009) conducted the most recent study of the NCI industrial worker cohort, with follow-up through 2004. They examined lymphohematopoietic risks based on exposure metrics including exposed (yes/ no), peak exposure, number of peak exposures ≥4.0 ppm, duration of exposure, average intensity of exposure, cumulative exposure, years since first exposure, and years since first exposure ≥4 ppm. Beane Freeman et al. (2009) stated that there was no evidence that risks increased with cumulative number of peaks ≥4.0 ppm or for duration of exposure for any lymphohematopoietic cancer evaluated, but they did not present results. An association was observed with the presence of at least one career peak exposure ≥4.0 ppm (risk ratio [RR] = 1.37, 95% CI: 1.03-1.81, *p*_trend_=.02 based on exposed subjects only and *p*_ttend_= -04 based on all study subjects), but not number of peak exposures ≥4.0 ppm. Risks were also increased with increasing peak intensity with follow-up to 1981 (*p*_trend_ = 0.00987 based on exposed subjects only and *p*_ttend_ = 0.0485 based on all study subjects), but not with follow-up from 1981-1994 or 1995-2004. Risks were lower in those with no exposure vs. those with their first exposure to ≥ 4 ppm formaldehyde 0-25 years earlier (RR = 0.57,95% CI: 0.36-0.88). This was consistent with results of Hauptmann et al. (2003), who followed this cohort through 1994. In their reanalysis of this cohort through 1994, Beane Freeman et al. (2009) found that, of the six exposure metrics, associations were only observed for peak exposure ≥0.04 ppm (RR=1.48, 95% CI: 1.04-2.12, *p*_ttend_= -02 including or excluding unexposed subjects).

#### 4.2.2. Cancer of lymphoid origin

Risks from cancers of lymphoid origin were examined in four cohorts (Table 5). Both Dell and Teta (1995) and Chiazze et al. (1997) defined cancers of lymphoid origin as those in ICD-7 200-205 categories. Whereas Chiazze et al. (1997) did not report increased risks, Dell and Teta (1995) reported increased risks among plastics manufacturers (standardized mortality rate [SMR] = 1.69, 95% CI: 1.07-2.53). No significant associations were found in the NCI embalmers cohort based on any of the seven exposure metrics evaluated (Hauptmann et al., 2009). Analyses of peak exposure, average intensity, cumulative exposure, cumulative number of peaks ≥4.0 ppm, or duration of employment also did not indicate any associations in the NCI industrial cohort (Beane Freeman et al., 2009).

**Table 5a:** Association between formaldehyde and cancers of lymphoid origin (ICD 200–204)

	Hauptmann et al., 2009					Beane Freeman et al., 2009				
		
	Embalmers case-control					NCI cohort (1934-2004)				
		
Measures	Category	Obs	Estimate		95% CI	Category	Obs	Estimate		95% CI
Unexposed/Exposed	Never embalming	18	OR	1.00	—					
	Ever embalming	81	OR	1.10	0.50-2.10					
Peak Exposure	0 ppm	18	OR	1.00	—	0 ppm	26	RR	1.17	0.72–1.89
	>0–7.0 ppm	29	OR	1.20	0.60–2.70	>0–<2.0 ppm	73	RR	1.00	—
	>7.0–9.3 ppm	37	OR	1.50	0.70–3.20	2.0–<4.0 ppm	56	RR	1.27	0.89–1.82
	>9.3 ppm	15	OR	0.60	0.20–1.30	≥4.0 ppm	74	RR	1.35	0.97–1.89
	*p*_trend_ = .111 (exposed)					*p*_trend_ = .06 (exposed)				
	*p*_trend_ = .523 (exposed and unexposed)					*p*_trend_ = .10 (exposed and unexposed)				
Average Intensity	0 ppm	18	OR	1.00	—	0 ppm	26	RR	1.08	0.68–1.71
	>0–1.4 ppm	34	OR	1.40	0.60–2.90	>0–<0.5 ppm	116	RR	1.00	—
	>1.4–1.9 ppm	26	OR	1.00	0.50–2.20	0.5–<1.0 ppm	49	RR	1.36	0.97–1.9
	>1.9 ppm	21	OR	0.90	0.40–1.90	≥1.0 ppm	38	RR	1.05	0.72–1.53
	*p*_trend_ = .287 (exposed)					*p*_trend_ > .5 (exposed)				
	*p*_trend_ = .598 (exposed and unexposed)					*p*_trend_ > .5 (exposed and unexposed)				
Cumulative Exposure	0 ppm-h	18	OR	1.00	—	0 ppm-yr	26	RR	0.94	0.59–1.49
	>0.4058 ppm-h	23	OR	0.90	0.40–2.00	>0–<1.5 ppm-yr	123	RR	1.00	—
	>4058–9253 ppm-h	33	OR	1.30	0.60–2.80	>1.5–<5.5 ppm-yr	30	RR	0.65	0.44–0.98
	>9253 ppm-h	25	OR	1.00	0.40–2.00	≥5.5 ppm-yr	50	RR	1.06	0.75–1.49
	*p*_trend_ = .912 (exposed)					*p*_trend_ > .5 (exposed)				
	*p*_trend_ = .965 (exposed and unexposed)					*p*_trend_ >.5 (exposed and unexposed)				
Cumulative number of peaks ≥4.0 ppm					No association. Results not shown.					
Duration of Exposure/Employment	0 yrs	18	OR	1.00	—	No association. Results not shown.				
	>0–20 yrs	16	OR	0.70	0.30–1.60					
	>20–34 yrs	32	OR	1.20	0.60–2.60					
	>34 yrs	33	OR	1.20	0.60–2.50					
	*p*_trend_ = .360 (exposed)									
	*p*_trend_ = .449 (exposed and unexposed)									
Number of Embalmings	0	18	OR	1.00	—					
	>0–1422	17	OR	0.70	0.30–1.60					
	>1422–3068	37	OR	1.50	0.70–3.00					
	>3068	27	OR	1.00	0.50–2.20					
	*p*_trend_ = .963 (exposed)									
	*p*_trend_ = .865 (exposed and unexposed)									
8-Hour Time-Weighted Average Intensity	0	18	OR	1.00	—					
	>0–0.10	32	OR	1.20	0.60–2.60					
	>0.10–0.18	25	OR	1.00	0.50–2.10					
	>0.18	24	OR	1.00	0.50–2.10					
	*p*_trend_ = .766 (exposed)									
	*p*_trend_ = .605 (exposed and unexposed)									

**Table 5b:** Other cohorts

Reference	Obs	Estimate		95% CI
Dell and Teta, 1995*	23	SMR	1.69	1.07–2.53
Chiazze et al., 1997*	5	SMR	0.46	0.15–1.08

Note:

* ICD-7 200–205.

#### 4.2.3. Leukemia

A large number of investigations have focused on the association between formaldehyde and leukemia (Tables 6, 7, 8, and 9). The types of leukemia investigated vary among studies, and this section focuses on analyses of all leukemia and aleukemias (leukemias in which the circulating white blood cells are normal or decreased in number) combined (ICD-7 204) and lymphatic, myeloid, monocytic, other, and unspecified leukemias combined (ICD-8 204-207 and ICD-9 204-208), whereas later sections discuss assessments of specific types of leukemia. Risk estimates for leukemia among 28 analyses that did not assess exposure-response were generally null (Table 6, table 6C). Only two cohort studies, conducted by Walrath and Fraumeni (1984) and Dell and Teta (1995), reported increased proportions or risks (PMR = 1.5, *p* <.05 and SMR = 2.65, 95% CI: 1.15-5.24, respectively).

**Table 6a:** Association between formaldehyde and leukemia (ICD 204–207)

	Beane Freeman et al., 2009					Pinkerton et al., 2004					Stern et al., 1987				
			
	NCI cohort (1934–2004)					US garment workers					Leather tannery workers				
			
Measures	Category	Obs	Estimate		95% CI	Category	Obs	Estimate		95% CI	Category	Obs	Estimate		95% CI
Unexposed/exposed	Unexposed	7	SMR	0.48	0.23–1.01	Exposed	24	SMR	1.09	0.70.1–62					
	Exposed	116	SMR	1.02	0.85.1–22										
Peak exposure	0 ppm	7	RR	0.59	0.25.1–36										
	>0–<2.0 ppm	41	RR	1.00	—										
	2.0–<4.0 ppm	27	RR	0.98	0.60.1–62										
	≥4.0 ppm	48	RR	1.42	0.92–2.18										
	*p*_trend_ = .12 (exposed)														
	*p*_trend_ = .02 (exposed and unexposed)														
Average intensity	0 ppm	7	RR	0.54	0.24.1–22										
	>0–<0.5 ppm	67	RR	1.00	—										
	0.5–<1.0 ppm	25	RR	1.13	0.71–1.79										
	≥1.0 ppm	24	RR	1.10	0.68.1–78										
	*p*_trend_ > .5 (exposed)														
	*p*_trend_ = .5 (exposed and unexposed)														
Cumulative exposure	0 ppm-yr	7	RR	0.53	0.23.1–21										
	>0–<1.5 ppm-yr	63	RR	1.00	—										
	>1.5–<5.5 ppm-yr	24	RR	0.96	0.60.1–56										
	≥5.5 ppm-yr	29	RR	1.11	0.70–1.74										
	*p*_trend_ = .12 (exposed)														
	*p*_trend_ = .08 (exposed and unexposed)														
Cumulative number of peaks ≥4.0 ppm	No association. Results not shown.														
Duration of exposure/employment	No association. Results not shown.					<3 yrs	7	SMR	0.96	—	<1 yr	2	SMR	0.45	0.05–1.68
						3–9 yrs	5	SMR	0.72	—	1–9 yrs	2	SMR	1.00	0.11–3.61
						10+ yrs	12	SMR	1.53	—	10+ yrs	6	SMR	1.70	0.63–3.73
						*p*_trend_ > .05									
Time since first exposure	0 yrs	5	RR	0.28	0.06.1–32	<10 yrs	2	SMR	0.68	—					
	>0–15 yrs	6	RR	1.00	–	<10.19 yrs	3	SMR	0.65	—					
	>15–25 yrs	22	RR	2.13	0.64.7–15	20+ yrs	19	SMR	1.31	—					
	>25–35 yrs	26	RR	0.94	0.25.3–51	*p*_trend_ > .05									
	>35 yrs	64	RR	0.53	0.14.2–09										
Year of first exposure						<1963	19	SMR	1.23	—					
						1963–1970	4	SMR	0.81	—					
						≥1971	1	SMR	0.56	—					
						*p*_trend_ > .05									
Time Since First Exposure ≥4 ppm	0 yrs	75	RR	0.34	0.18.0–67										
	>0–25 yrs	14	RR	1.00	–										
	>25–42 yrs	16	RR	0.37	0.16.0–83										
	>42 yrs	18	RR	0.51	0.22.1–19										

**Table 6b:** Other cohorts

Reference	ICD code	Obs	Estimate		95% CI
Harrington and Shannon, 1975 (pathologists)	204–207	1	SMR	0.63	0.02–3.48
Harrington and Shannon, 1975 (technicians)	204–207	1	SMR	0.45	0.01–2.53
Walrath and Fraumeni, 1983	204–207	12	PMR†	1.40	na
Wong	et	al., 1983	204–207	2	SMR†
Levine	et	al., 1984	204–207	4	SMR‡
Walrath and Fraumeni, 1984	204–207	12	PMR†	1.50	*p* < .05
Logue et al., 1986 (radiologists)	204¶	na	SMR	1.55	na
Logue et al., 1986 (pathologists)	204¶	na	SMR	1.06	na
Stroup et al., 1986	204–207	10	SMR	1.50	0.70–2.70
Robinson et al., 1987	204¶	1	SMR¶	0.59	0.01–3.28
Stern et al., 1987 (Plant	A)	204¶	4	SMR†	0.70
Stern et al., 1987 (Plant	B)	204¶	6	SMR†	0.75
Stern et al., 1987 (Finishing	Department)	204¶	7	SMR†	1.25
Ott et al., 1989*	Non-lymphocytic	2	OR	2.6	na
Ott et al., 1989*	Lymphocytic	1	OR	2.60	na
Linos et al., 1990*	na	4	OR	2.10	0.40–10.00
Hall et al., 1991	204–207	4	SMR	2.63	0.41–3.89
Matanoski et al., 1991	na	31	SMR	1.35	0.92–1.92
Partanen et al., 1993	204¶	2	OR	1.40	0.25–7.91
Andjelkovich et al., 1995	204–207	2	SMR†	0.43	0.05–1.57
Dell and Teta, 1995	204¶	8	SMR†	2.65	1.15–5.24
Hansen and Olsen, 1995	204¶	39	SPIR	0.80	0.60–1.60
Chiazze et al., 1997	204¶	1	SMR	0.24	0.006–1.36
Stellman et al., 1998	na	12	RR	0.96	0.54–1.71
Blair et al., 2001*	na	64	OR‡	0.98	0.70–1.36
Coggon et al., 2003	204–208	31	SMR	0.91	0.62–1.29
Ambroise et al., 2005	204–208	1	SMR	4.42	0.11–24.64

Note:

na = not available.

* Case-control study.

† SMR or PMR values divided by 100.

‡ Risk estimate not provided in original citation, value calculated by Bachand et al. (2010).

¶ ICD-7.

There were no increased risks of leukemia in any formaldehyde exposure group among the three studies that assessed exposure-response and, with one exception, no exposure-response associations were reported. Stern et al. (1987) found no association with duration of employment as a leather tannery worker and Pinkerton et al. (2004) found risks in garment workers were not related with duration of exposure, time since first exposure, or year of first exposure (Table 6). Beane Freeman et al. (2009) examined associations with formaldehyde in the NCI industrial worker cohort by peak exposure, average intensity, cumulative exposure, cumulative number of peaks ≥4.0 ppm (data not reported), and duration of exposure (data not reported), years since first exposure, and years since first exposure ≥4 ppm, including and excluding a referent group with no exposure. They found no trends except for peak exposure when all exposure groups were included (*p*_trend_ = .02) but not when the referent group was excluded (*p*_trem_, =.12). In this cohort, risks were lower in those with no exposure vs. those with their first exposure to ≥4 ppm formaldehyde 0-25 years earlier (RR = 0.34, 95% CI: 0.18-0.67) and also in those whose first exposure to ≥4 ppm formaldehyde was 25-42 years earlier vs. 0-25 years earlier (RR = 0.37, 95% CI: 0.16-0.83). The RR estimates in the NCI industrial worker cohort are similar to those reported in the previous follow-up of this cohort to 1994 (e.g., for peak exposure ≥4.0 ppm, RR_through_ _1994_ = 1-60, 95% CI: 0.90-2.82 vs. RR_through2004_= 1-42,95% CI: 0.92-2.18) (Beane Freeman et al., 2009; Hauptman et al., 2003). In this cohort, risks were lower in those with no exposure vs. those with their first exposure to ≥4 ppm formaldehyde 0-25 years earlier (RR = 0.34, 95% CI: 0.18-0.67) and also in those whose first exposure to ≥4 ppm formaldehyde was 25-42 years earlier vs. 0-25 years earlier (RR = 0.37, 95% CI: 0.16-0.83).

#### 4.2.4. Lymphatic leukemia

Results from analyses of lymphatic leukemia (ICD 204) are similar to those reported for all leukemias combined (Tables 6 and 7). Among the four studies that assessed lymphatic leukemia, all risk estimates are null (Table 7, table 7A). Blair et al. (2001) and Pinkerton et al. (2004) reported no association between formaldehyde exposure and mortality from lymphatic leukemia. There were no exposure-response relationships for any of the six exposure metrics evaluated by Beane Freeman et al. (2009) in the NCI industrial cohort. This result was also observed in this cohort with follow-up only through 1994 (Beane Freeman et al., 2009; Hauptmann et al., 2003).

**Table 7a:** Association between formaldehyde and lymphatic leukemia (ICD 204)

	Beane Freeman et al., 2009					Blair et al., 2001				
		
	NCI cohort (1934–2004)					US industrial workers				
		
Measures	Category	Obs	Estimate		95% CI	Category	Obs	Estimate		95% CI
						Acute				
Unexposed/Exposed	Unexposed	1	SMR	0.26	0.04–1.82	Low	0	OR	—	—
	Exposed	36	SMR	1.15	0.83.1.59	High	0	OR	—	—
						Chronic				
						Low	29	OR	1.20	0.70–1.80
						High	1	OR	0.60	0.10–5.30
Peak Exposure	0 ppm	1	RR	0.27	0.03–2.13					
	>0–<2.0 ppm	14	RR	1.00	—					
	2.0–<4.0 ppm	8	RR	0.81	0.33–1.96					
	≥4.0 ppm	14	RR	1.15	0.54–2.47					
	*p*_trend_ > 0.5 (exposed)									
	*p*_trend_ = 0.3 (exposed and unexposed)									
Average Intensity	0 ppm	1	RR	0.26	0.03–2.01					
	>0–<0.5 ppm	22	RR	1.00	—					
	0.5–<1.0 ppm	7	RR	0.92	0.39–2.16					
	≥1.0 ppm	6	RR	1.61	0.76–3.39					
	*p*_trend_ > 0.5 (exposed)									
	*p*_trend_ > 0.5 (exposed and unexposed)									
Cumulative Exposure	0 ppm-yr	1	RR	0.24	0.03–1.88					
	>0–<1.5 ppm-yr	21	RR	1.00	—					
	>1.5–<5.5 ppm-yr	5	RR	0.57	0.21–1.54					
	≥5.5 ppm-yr	10	RR	1.02	0.47–2.21					
	*p*_trend_ = 0.46 (exposed)									
	*p*_trend_ = 0.41 (exposed and unexposed)									
Cumulative number of peaks ≥4.0 ppm	No association. Results not shown.									
Duration of Employment	No association. Results not shown.									

**Table 7b:** Other cohorts

Reference	ICD Code	Obs	Estimate		95% CI
Hayes et al., 1990	204	7	PMR *	0.7	0.29–1.53
Pinkerton et al., 2004	204	3	SMR	0.60	0.12–1.75

Note: na = not available

* PMR divided by 100.

#### 4.2.5. Hematopoietic cancer of non-lymphoid origin

Associations between formaldehyde exposure and hematopoietic cancers of non-lymphoid origin were investigated in several studies (Table 8). In an early analysis of the NCI embalmers cohort, Hayes et al. (1990) found the PMR from polycythaemia vera or myelofibrosis was not higher than expected, but it was from monocytic leukemia, other (i.e., not lymphatic, myeloid, or monocytic), and unspecified leukemias combined (PMR = 2.28, 95% CI: 1.29-3.52). Pinkerton et al. (2004) found no association with monocytic leukemia or leukemia of other or unspecified type among garment workers (SMR = 0.92, 95% CI: 0.34-2.00).

**Table 8a:** Association between formaldehyde and cancers of non-lymphoid origin (ICD 205, 206, 208, 209)

	Hauptmann et al., 2009*					Beane Freeman et al., 2009				
		
	Embalmers case-control					NCI cohort (1934–2004)				
		
Measures	Category	Obs	Estimate		95% CI	Category	Obs	Estimate		95% CI
Unexposed/Exposed	Never embalming	4	OR	1.00	—					
	Ever embalming	44	OR	3.00	1.00–9.50					
Peak Exposure	<500 embalmings	9	OR	1.00	—	0 ppm	5	RR	1.01	0.34–2.98
	≥7.0 ppm	10	OR	1.60	0.60.4.50	>0–<2.0 ppm	15	RR	1.00	—
	>7.0–9.3 ppm	12	OR	1.40	0.50–3.70	2.0–<4.0 ppm	11	RR	1.19	0.54–2.62
	>9.3 ppm	17	OR	2.30	0.90–5.60	≥4.0 ppm	21	RR	1.80	0.91–3.57
						*p*_trend_ = .09 (exposed)				
						*p*_trend_ = .09 (exposed and unexposed)				
Average Intensity	<500 embalmings	9	OR	1.00	—	0 ppm	5	RR	0.89	0.32.2.5
	≤1.4 ppm	13	OR	1.70	0.70–4.50	>0–<0.5 ppm	25	RR	1.00	—
	>1.4–1.9 ppm	12	OR	1.70	0.70–4.60	0.5–<1.0 ppm	11	RR	1.40	0.68–2.86
	>1.9 ppm	14	OR	1.80	0.70–4.70	≥1.0 ppm	11	RR	1.51	0.72–3.16
						*p*_trend_ > .5 (exposed)				
						*p*_trend_ > .5 (exposed and unexposed)				
Cumulative Exposure	<500 embalmings	9	OR	1.00	—	0 ppm–yr	5	RR	0.69	0.25–1.95
	≤4058 ppm-h	5	OR	1.10	0.30–3.80	>0–<1.5 ppm-yr	30	RR	1.00	—
	>4058–9253 ppm-h	12	OR	1.40	0.50–3.70	>1.5–<5.5 ppm-yr	7	RR	0.61	0.26–1.41
	>9253 ppm-h	22	OR	2.40	1.00–5.80	≥5.5 ppm-yr	10	RR	0.86	0.41–1.81
						*p*_trend_ > .5 (exposed)				
						*p*_trend_ > .5 (exposed and unexposed)				
Cumulative number of peaks ≥4.0 ppm						No association. Results not shown.				
Duration of Exposue/Employment	<500 embalmings	9	OR	1.00	—	No association. Results not shown.				
	≥20 yrs	2	OR	0.30	0.10–1.70					
	>20–34 yrs	16	OR	2.00	0.80–5.00					
	>34 yrs	21	OR	2.60	1.00–6.40					
Number of Embalmings	<500 embalmings	9	OR	1.00	—					
	≥500–1422	3	OR	0.60	0.20–2.60					
	>1422–3068	15	OR	1.80	0.70–4.60					
	>3068	21	OR	2.30	1.00–5.70					
8–Hour Time-Weighted Average Intensity	<500 embalmings	9	OR	1.00	—					
	≤0.10 ppm	9	OR	1.30	0.50–3.60					
	>0.10–0.18 ppm	16	OR	2.10	0.80–5.30					
	>0.18 ppm	14	OR	1.90	0.70–4.80					

Note:

* Results from analyses using those who never embalmed as a referent group (with one myeloid leukemia case) were highly unstable. Results presented here are from analyses using individuals with <500 embalmings as the referent group (see Table 4 in Hauptmann et al., 2009).

**Table 8b:** Other cohorts

Reference	Code	Obs	Estimate		95% CI
Pinkerton et al., 2004	206–208	6	SMR	0.92	0.34–2.00
Hayes et al., 1990	206, 207	20	PMR	2.28	1.39–3.52
Hayes et al., 1990	208	3	PMR	3.90	0.80–11.38
Hayes et al., 1990	209	4	PMR	2.62	0.42–3.91

Risks of myeloid leukemia (ICD 205), monocytic leukemia (ICD 206), ploycthaemia vera (ICD 208), and myelofibrosis (ICD 209) combined were examined in recent studies of the NCI industrial worker and embalmer cohorts (Hauptmann et al., 2009; Beane Freeman et al., 2009; Table 8). Beane Freeman et al. (2009) did not report any excess risks in the industrial worker cohort based on analyses by peak exposure, average intensity, cumulative exposure, cumulative number of peaks ≥4.0 ppm, or duration of exposure (Table 8). They also found no exposure-response associations among analyses including or excluding the unexposed population. This is consistent with previous analyses of this cohort (Hauptmann et al., 2003; Blair et al., 1986).

Hautpmann et al. (2009) found that risk estimates from analyses using subjects who never embalmed as a referent category were highly unstable because of the small number of cases in this category *(n =* 4, odds ratio [OR] =3.0, 95% CI: 1.0-9.5 for ever vs. never embalmed). Still, among six exposure metrics, there were no exposure-response associations reported when unexposed referents (i.e., 0 embalmings) were included or excluded with one exception—there was a trend reported with duration of exposure when the unexposed group was excluded (*p*_trend_ = .046) but not when it was included (*p*_trend_ = .348). Because of the issues with the aforementioned analyses, Hauptmann et al. (2009) also conducted analyses using those who performed <500 embalmings as a referent category. Results from these analyses, which they suggest are more reliable, are presented in Table 8. The majority of risk estimates were null, except for the highest exposure group for cumulative exposure (>34 years, OR = 2.60, 95% CI: 1.0-6.4) and number of embalmings (>3068 embalmings, OR = 2.3, 95% CI: 1.00-5.70). Hauptmann et al. (2009) also reported that among those who embalmed for more than 20 years, a significant increased risk of non-lymphoid cancers was observed (OR = 3.5, 95% CI: 1.1-10.9). The *p* values reported for the trend tests by Hauptman et al. (2009) are incorrect, as they are the same as those reported for the tests which used 0 embalmers (vs. <500) as the referent category; therefore, they are not reported here.

#### 4.2.6. Myeloid leukemia

Myeloid leukemia was assessed in three case-control studies and four cohort studies, some of which also analyzed acute and/or chronic subtypes (Table 9). Results varied among the four studies that compared risks in exposed vs. unexposed individuals. Stroup et al. (1986) reported an excess in myeloid leukemia in US anatomists (SMR = 8.8, 95% CI: not reported). Similarly, Linos et al. (1990) reported an excess of acute myeloid leukemia in funeral home workers, although this was based on three exposed cases (OR = 6.7, 95% CI: 1.2-36.2). Hayes et al. (1990) reported a significant excess proportion of myeloid leukemia deaths overall in the NCI embalmers

**Table 9a:** Association between formaldehyde and myeloid leukemia (ICD 205).

	Hauptmann et al., 2009^*^ (ICD 205)					Hauptmann et al., 2009* (ICD 205.0)					Beane Freeman et al., 2009					Pinkerton et al., 2004					Blair et al., 2001				
					
	Embalmers case-control					Embalmers case-control					NCI Cohort (1934–2004)					US garment workers					US industrial workers				
					
Measures	Category	Obs	Estimate		95% CI	Category	Obs	Estimate		95% CI	Category	Obs	Estimate		95% CI	Category	Obs	Estimate		95% CI	Category	Obs	Estimate		95% CI
Unexposed/Exposed	Never embalming	1	OR	1.00	—						Unexposed	4	SMR	0.65	0.25–1.74						Acute				
	Ever embalming	33	OR	11.20	1.3-95.6						Exposed	44	SMR	0.90	0.67–1.21	Exposed	15	SMR	1.44	0.80–2.37	Low	14	OR	0.9	0.50–1.60
																					High	0	OR	—	—
																Acute	9	SMR	1.34	0.61–2.54					
																Chronic	4	SMR	1.39	0.38–3.56	Chronic				
																Other/unspecified	1	SMR	2.15	0.05–11.94	Low	7	OR	1.3	0.60–3.10
																					High	1	OR	2.9	0.30–24.50
Peak Exposure	<500 embalmings	5	OR	1.00	—	<500 embalmings	3	OR	1.00	—	0 ppm	4	RR	0.82	0.25–2.67										
	>0–7.0 ppm	9	OR	2.90	0.9–9.8	>0–7.0 ppm	4	OR	1.80	0.4–9.3	>0–<2.0 ppm	14	RR	1.00 —											
	>7.0–9.3 ppm	9	OR	2.00	0.6–6.6	>7.0–9.3 ppm	5	OR	2.10	0.5–9.2	2.0–<4.0 ppm	11	RR	1.30	0.58–2.92										
	>9.3 ppm	11	OR	2.90	0.9–9.5	>9.3 ppm	7	OR	2.90	0.7–12.5	≥4.0 ppm	19	RR	1.78	0.87–3.64										
											*p*_trend_ = .13 (exposed)														
											*p*_trend_ = .07 (exposed and unexposed)														
Average Intensity	<500 embalmings	5	OR	1.00	—	<500 embalmings	3	OR	1.00	—	0 ppm	4	RR	0.70	0.23–2.16										
	>0–1.4 ppm	10	OR	2.60	0.8–8.7	>0–1.4 ppm	6	OR	2.50	0.6–10.9	>0–<0.5 ppm	24	RR	1.00	—										
	>1.4–1.9 ppm	10	OR	2.80	0.8–9.1	>1.4–1.9 ppm	5	OR	2.00	0.4–9.4	0.5–<1.0 ppm	9	RR	1.21	0.56–2.62										
	>1.9 ppm	9	OR	2.30	0.7–7.5	>1.9 ppm	6	OR	2.30	0.5–10.3 ≥1.0 ppm	11	RR	1.61	0.76–3.39											
											*p*_trend_ = .43 (exposed)														
											*p*_trend_ = .40 (exposed and unexposed)														
Cumulative exposure	<500 embalmings	5	OR	1.00	—	<500 embalmings	3	OR	1.00	—	0 ppm-yr	4	RR	0.61	0.2–1.91										
	>0–4058 ppm-h	5	OR	2.10	0.5–8.1	>0–4058 ppm-h	2	OR	1.30	0.2–9.4	>0–<1.5 ppm-yr	26	RR	1.00	—										
	>4058–9253 ppm-h	10	OR	2.20	0.7–7.1	>4058–9253 ppm-h	6	OR	1.90	0.4–8.2	>1.5–<5.5 ppm-yr	8	RR	0.82	0.36–1.83										
	>9253 ppm-h	14	OR	3.10	1.0–9.6	>9253 ppm-h	9	OR	3.20	0.8–13.1	≥5.5 ppm–yr	10	RR	1.02	0.48–2.16										
											*p*_trend_ > .5 (exposed)														
											*p*_trend_ = .44 (exposed and unexposed)														
Cumulative number of peaks ≥4.0 ppm										No association. Results not shown.															
Duration of exposure/employment	<500 embalmings	5	OR	1.00	—	<500 embalmings	3	OR	1.00	—					<3 yrs	3	SMR	0.83	—						
	>0–20 yrs	2	OR	0.50	0.1–2.9	>0–20 yrs	1	OR	0.40	0.04–4.9	No association. Results not shown.					3–9 yrs	4	SMR	1.26	—					
	>20–34 yrs	13	OR	3.20	1.0–10.1	>20–34 yrs	8	OR	2.90	0.7–12.2					10+ yrs	8	SMR	2.19	—						
	>34 yrs	14	OR	3.90	1.2–12.5	>34 yrs	8	OR	3.10	0.7–13.7					*p*_trend_ > .05										
Time since first exposure															<10 yrs	1	SMR	0.90	—						
																<10–19 yrs	1	SMR	0.40	—					
																20+ yrs	13	SMR	1.91	†					
																*p*_trend_ > .05									
Number of embalmings	<500 embalmings	5	OR	1.00	—	0	3	OR	1.00	—															
	>0–1422	3	OR	1.20	0.30–5.50	>0–1422	0	OR	0.00	0.0–1.8															
	>1422–3068	12	OR	2.90	0.90–9.10	>1422–3068	8	OR	2.90	0.7–12.0															
	>3068	14	OR	3.00	1.00–9.20	>3068	9	OR	2.90	0.7–11.6															
8-Hour time-weighted average intensity	<500 embalmings	5	OR	1.00	—	0	3	OR	1.00	—															
	>0–0.10	8	OR	2.40	0.70–8.20	>0–0.10	3	OR	1.40	0.3–7.8															
	>0.10–0.18	10	OR	2.60	0.80–8.70	>0.10–0.18	7	OR	2.60	0.6–11.4															
	>0.18	11	OR	2.60	0.80–8.30	>0.18	7	OR	2.60	0.6–11.3															

**Table 9b:** Other cohorts.

Reference	Obs	Estimate		95% CI
Stroup et al., 1986	5	SMR	8.8	—
Hayes et al., 1990	24	PMR‡	1.57	1.01–2.34
Linos et al., 1990 (acute)	3	OR	6.70	1.20–36.20

*Results from analyses using those who never embalmed as the referent group (with one myeloid leukemia case) were highly unstable. Results presented here are from analyses using individuals with <500 embalmings as the referent group. (See Table 4 in Hauptman et al., 2009).

†95% CI does not include 1.0.

‡ PMR divided by 100.

cohort (PMR = 1.57, 95% CI: 1.01–2.34), but found no associations in analyses by subtype (PMR_acute_ = 1.52, 95% CI: 0.85–2.52; PMR_chronic_ = 1.84, 95% CI: 0.79–3.62). Blair et al. (2001) conducted a case-control study of several industrial and occupational job categories in US workers and found no associations between intensity of formaldehyde exposure and acute or chronic myeloid leukemia.

Pinkerton et al. (2004) assessed myeloid leukemia in a cohort of US garment workers and found no association with formaldehyde exposure overall (SMR 1.44, 95% CI: 0.80-2.37) or when examined by subtype (SMR_acute_= 1.34, 95% CI: 0.61–2.54; SMR_chronic_ = 1.39, 95% CI: 0.38–3.56). There were also no trends with duration of exposure or time since first exposure (*p*>.05), although risks were increased in workers with 20 or more years since first exposure (SMR = 1.91, 95% CI: not reported). In contrast, there were no increased risks in workers exposed for 10 or more years with 20 or more years since first exposure overall (SMR = 2.43, 95% CI: 0.98-5.01) or in analyses limited to acute myeloid leukemia (SMR = 2.51, 95% CI: 0.81-5.85).

In an analysis of the NCI industrial worker cohort with follow-up through 2004, Beane Freeman et al. (2009) assessed whether myeloid leukemia risk was associated with formaldehyde estimated as peak exposure, average intensity, cumulative exposure, cumulative number of peaks ≥4.0 ppm (data not reported), duration of exposure (data not reported), years since first exposure, and years since first exposure ≥4 ppm. These investigators reported no associations between any exposure metric and myeloid leukemia, including peak exposure (RR = 1.78, 95% CI: 0.87-3.64, *p*_ttend_ = 0.13 for exposed groups), except for lower risks in those with no exposure vs. those with their first exposure to ≥ 4 ppm formaldehyde 0-25 years earlier (RR = 0.30, 95% CI: 0.11-0.81) and higher risks with increasing peak intensity with follow-up from 1981-1994 (*p*_trend_ = 0.0353 based on exposed subjects only and *p*_trend_ = 0.210 based on all study subjects), but not with follow-up to 1981 or 1995-2004 (Table 9). These null results were consistent with analyses of this cohort through 1994based on every exposure metric except peak exposure, for which risks were increased (RR = 2.79, 95% CI: 1.08-7.21, *p*_ttend_=.02 for exposed groups, *p*_trend_=.0087 for all groups) (Beane Freeman et al., 2009; Hauptmann et al., 2003). There were no associations based on any other metric in analyses.

Hauptmann et al. (2009) conducted a case-control study of professional embalmers, including cases from previous studies (Walrath and Fraumeni, 1983, 1984; Hayes et al., 1990), and assessed myeloid leukemia risk based on seven formaldehyde exposure metrics (Table 9). Having ever embalmed was associated with myeloid leukemia (OR = 11.2, 95% CI: 1.3-95.6, *p*_ttend_=.027), but there was only one case who never embalmed, making this risk estimate highly unreliable. Because of this, Hauptmann et al. (2009) combined unexposed individuals and those with <500 embalmings as a referent group to provide more conservative and reliable risk estimates; these are discussed here and shown in Table 9. An increased risk for myeloid leukemia was not reported for any exposed group except those with more than 34 years of employment (OR = 3.9, 95% CI: 1.2-12.5), more than 3068 embalmings (OR = 3.0, 95% CI: 1.0-9.2), or more than 9253 ppm-hours of cumulative formaldehyde exposure (OR = 3.1, 95% CI: 1.0-9.6). Hauptmann et al. (2009) conducted similar analyses for acute myeloid leukemia and found no associations in any dose group. Reported *p* values for trend tests for total and acute myeloid leukemia appear to be those based on analyses using 0 embalmings (vs. <500 embalmings) as a referent category and are not presented here.

Although there are some isolated findings of statistically significant associations between formaldehyde exposure and myeloid leukemia, these have not been found consistently either within or among studies and are far outnumbered by null findings in the more robust studies.

#### 4.2.7. Other unspecified leukemia

Most cohort and case-control studies examined other (i.e., not lymphatic, myeloid, or monocytic) or unspecified leukemias (ICD 207) grouped with other lymphohe-matopoietic cancer types. The ICD 207 category alone was only examined in the NCI industrial worker cohort (Table 10). Beane Freeman et al. (2009) reported no associations between formaldehyde exposure and other or unspecified leukemia based on peak exposure, average intensity, cumulative exposure, cumulative number of peaks ≥4.0 ppm (data not reported), or duration of exposure (data not reported). These results are consistent with previous evaluations of the NCI industrial worker cohort (Hauptmann et al., 2003; Blair et al., 1986). Hayes et al. (1990) examined monocytic (ICD 206) and other unspecified leukemia (ICD 207) combined in embalm-ers and reported an increased proportion of deaths (PMR = 2.28, 95% CI: 1.29-3.52). This disease category was not evaluated in the follow-up by Hauptman et al. (2009).

**Table 10a:** Association between formaldehyde and other unspecified leukemia (ICD-8 207).

	Beane Freeman et al., 2009				
	
	NCI Cohort (1934-2004)				
	
Measures	Category	Obs	Estimate		95% CI
Peak Exposure	0 ppm	2	RR	0.61	0.13–2.85
	>0–<2.0 ppm	13	RR	1.00	—
	2.0–<4.0 ppm	8	RR	0.86	0.35–2.12
	≥4.0 ppm	13	RR	1.15	0.53.2.53
	*p*_trend_ > .5 (exposed)				
	*p*_trend_ = .5 (exposed and unexposed)				
Average Intensity	0 ppm	2	RR	0.58	0.13–2.62
	>0–<0.5 ppm	21	RR	1.00	—
	0.5–<1.0 ppm	7	RR	0.98	0.42–2.33
	≥1.0 ppm	6	RR	0.84	0.33.2.12
	*p*_trend_ > .5 (exposed)				
	*p*_trend_ > .5 (exposed and unexposed)				
Cumulative Exposure	0 ppm-yr	2	RR	0.77	0.16–3.59
	>0–<1.5 ppm-yr	15	RR	1.00	—
	>1.5–<5.5 ppm-yr	10	RR	1.65	0.73–3.73
	≥5.5 ppm-yr	9	RR	1.44	0.61.3.36
	*p*_trend_ = .15 (exposed)				
	*p*_trend_ = .13 (exposed and unexposed)				
Cumulative number of peaks .4.0 ppm	No association. Results not shown.				
Duration of Employment	No association. Results not shown.				

**Table 10b:** Other cohorts.

Reference	Code	Obs	Estimate		95% CI
Hayes et al., 1990	206, 207	20	PMR	2.28	1.39–3.52

#### 4.2.8. Hodgkin's lymphoma, non-Hodgkin's lymphoma, and multiple myeloma

Cohort and case-control study results for Hodgkin's and non-Hodgkin's lymphoma and multiple myeloma are presented in Tables 11 to 13. Eleven assessments of formaldehyde-exposed vs. unexposed workers did not show associations between exposure and Hodgkin's lymphoma (Table 10). In the one study that evaluated iron foundry workers vs. the general population, risks were also not increased (Andjelkovich et al., 1995). When exposure-response relationships were evaluated in the NCI industrial worker cohort, associations were reported for peak exposure (*p*_trend_ = .01 for exposed groups) and average intensity (*p*_trend_ = .05 for exposed groups) but not for cumulative exposure, cumulative number of peaks ≥4.0 ppm (data not reported), or duration of exposure (data not reported) (Beane Freeman et al., 2009). This is consistent with the earlier examination of the NCI industrial worker cohort, for which exposure-response relationships for peak exposure (*p*_ttend_= -04) and average intensity (*p*_ttend_ = -03), but not other exposure metrics, were reported (Beane Freeman et al., 2009; Hauptmann et al., 2003).

**Table 11a:** Association between formaldehyde and Hodgkin's lymphoma (ICD 201).

	Beane Freeman et al., 2009					Andjelkovich et al., 1995				
		
	NCI cohort (1934–2004)					Iron foundry workers				
		
Measures	Category	Obs	Estimate		95% CI	Category	Obs	Estimate		95% CI
Unexposed/Exposed	Unexposed	2	SMR	0.70	0.17–2.80	Unexposed	0	SMR	0.00	0.00–4.12
	Exposed	25	SMR	1.42	0.96–2.10	Exposed	1	SMR	0.72	0.01–4.00
Peak Exposure	0 ppm	2	RR	0.67	0.12–3.60					
	>0–<2.0 ppm	6	RR	1.00	—					
	2.0–<4.0 ppm	8	RR	3.30	1.04–10.50					
	≥4.0 ppm	11	RR	3.96	1.31.12.02					
	*p*_trend_ = .01 (exposed)									
	*p*_trend_ = .004 (exposed and unexposed)									
Average Intensity	0 ppm	2	RR	0.53	0.11–2.66					
	>0–<0.5 ppm	10	RR	1.00	—					
	0.5–<1.0 ppm	9	RR	3.62	1.41–9.31					
	≥1.0 ppm	6	RR	2.48	0.84.7.32					
	*p*_trend_ = .05 (exposed)									
	*p*_trend_ = .03 (exposed and unexposed)									
Cumulative Exposure	0 ppm-yr	2	RR	0.42	0.09–2.05					
	>0–<1.5 ppm-yr	14	RR	1.00	—					
	>1.5–<5.5 ppm-yr	7	RR	1.71	0.66–4.38					
	>5.5 ppm-yr	4	RR	1.30	0.4.4.19					
	*p*_trend_ = .08 (exposed)									
	*p*_trend_ = .06 (exposed and unexposed)									
Cumulative number of peaks ≥4.0 ppm	No association. Results not shown.									
Duration of Employment	No association. Results not shown.									

**Table 11b:** Other cohorts.

Reference	Obs	Estimate		95% CI
Wong et al., 1983	2	SMR	2.40	0.27-8.66
Robinson et al., 1987	2	SMR	3.33	0.59-10.49
Gerin et al., 1989	8	OR	0.50	0.20-1.40
Hayes et al., 1990	3	PMR	0.72	0.15-2.10
Hall et al., 1991	1	SMR	1.31	0.03-7.33
Matanoski et al., 1991	2	SMR	0.36	0.04-1.31
Hansen and Olsen, 1995	12	SPIR	1.00	0.50-1.70
Coggon et al., 2003	6	SMR	0.70	0.26-1.53
Pinkerton et al., 2004	2	SMR	0.55	0.07-1.98
Hauptmann et al., 2009	8	OR	0.50	0.10-2.60

None of the 13 epidemiology investigations reported associations between formaldehyde exposure and non-Hodgkin's lymphoma for any exposure metric evaluated (Table 12). There were also no exposure-response associations observed (Table 12).

**Table 12a:** Association between formaldehyde and non-Hodgkin's lymphoma (ICD 200, 202).

	Beane Freeman et al., 2009					Wang et al., 2009b					Gerin et al., 1989				
			
	NCI cohort (1934–2004)					Connecticut women (1996–2000)					Montreal workers (1979–1985)				
			
Measures	Category	Obs	Estimate		95% CI	Category	Obs	Estimate		95% CI	Category	Obs	Estimate		95% CI
Unexposed/Exposed	Unexposed	12	SMR	0.86	0.49–1.52	Never	398	OR	1.00	—					
	Exposed	94	SMR	0.85	0.70–1.05	Ever	203	OR	1.30	1.00–1.70					
Peak Exposure	0 ppm	12	RR	1.06	0.53–2.14										
	>0–<2.0 ppm	39	RR	1.00	—										
	2.0–<4.0 ppm	27	RR	1.08	0.65–1.78										
	≥4.0 ppm	28	RR	0.91	0.55.1.49										
						*p*_trend_ > .5 (exposed)									
						*p*_trend_ > .5 (exposed and unexposed)									
Average Intensity	0 ppm	12	RR	1.08	0.55–2.12	Never	398	OR	1.00	—					
	>0–<0.5 ppm	59	RR	1.00	—	Low	129	OR	1.40	1.00–1.80					
	0.5–<1.0 ppm	22	RR	1.20	0.73–1.96	Medium–High	74	OR	1.20	0.80–1.70					
	≥1.0 ppm	13	RR	0.71	0.39.1.32	*p*_trend_ = .21									
						*p*_trend_ > .5 (exposed)									
						*p*_trend_ = .45 (exposed and unexposed)									
Cumulative Exposure	0 ppm-yr	12	RR	0.94	0.46–1.86						Short	13	OR	0.70	0.30–1.60
	>0–<1.5 ppm-yr	60	RR	1.00	-						Long—low	15	OR	1.10	0.50–2.20
	>1.5–<5.5 ppm-yr	13	RR	0.58	0.31–1.06						Long—medium	14	OR	1.00	0.50–2.10
	≥5.5 ppm-yr	21	RR	0.91	0.54–1.52						Long—high	5	OR	0.50	0.10–1.70
						*p*_trend_ > .5 (exposed)									
						*p*_trend_ = .42 (exposed and unexposed)									
Cumulative number of peaks ≥4.0 ppm						No association. Results not shown.									
Duration of Employment						No association. Results not shown.									

**Table 12b:** Other cohorts.

Reference	Obs	Estimate	95% CI	
Edling et al., 1987	2	SPIR *	2.00	0.50–7.20
Ott et al., 1989†	2	OR	2.00	—
Hayes et al., 1990	34	PMR	1.26	0.87–1.76
Linos et al., 1990†	6	OR	3.2	0.80–13.40
Partanen et al., 1993†	4	OR	4.24	0.68–26.60
Hansen and Olsen, 1995	32	SPIR	0.90	0.60–1.20
Tatham et al., 1997†	93	OR	1.20	0.86–1.50
Stellman et al., 1998	11	RR	0.92	0.50–1.68
Coggon et al., 2003	31	SMR	0.98	0.67–1.39
Hauptmann et al., 2009	NR	OR	0.90	0.40–2.10

* Notes Standardized proportionate incidence ratio.

† Case-control study.

Multiple myeloma was not associated with formaldehyde exposure in any of the eight groups studied (Table 13). In the NCI industrial worker cohort, multiple myeloma risk was higher in individuals with no exposure based on all measures evaluated (Table 13). The only association with formaldehyde reported was for peak exposure ≥4.0 ppm (RR = 2.04, 95% CI: 1.01-4.12); however, the trend was not significant (*p*> .05) and there was no association with the number of peak exposures ≥4.0 ppm. This finding was consistent with results from earlier follow-ups of this cohort (Beane Freeman et al., 2009; Hauptmann et al., 2003).

**Table 13a:** Association between formaldehyde and multiple myeloma (ICD 203).

	Beane Freeman et al., 2009				
	
	NCI cohort (1934-2004)				
	
Measures	Category	Obs	Estimate		95% CI
Unexposed/eEposed	Unexposed	11	SMR	1.78	0.99–3.22
	Exposed	48	SMR	0.94	0.71–1.25
Peak Exposure	0 ppm	11	RR	2.74	1.18–6.37
	>0–<2.0 ppm	14	RR	1.00	—
	2.0–<4.0 ppm	13	RR	1.65	0.76–3.61
	≥4.0 ppm	21	RR	2.04	1.01.4.12
	*p*_trend_ = .08 (exposed)				
	*p*_trend_ > .5 (exposed and unexposed)				
Average Intensity	0 ppm	11	RR	2.18	1.01–4.70
	>0–<0.5 ppm 25 RR 1.00 —				
	0.5–<1.0 ppm	11	RR	1.40	0.68–2.86
	>1.0 ppm	12	RR	1.49	0.73.3.04
	*p*_trend_ > .5 (exposed)				
	*p*_trend_ > .5 (exposed and unexposed)				
Cumulative Exposure	0 ppm-yr	11	RR	1.79	0.83–3.89
	>0–<1.5 ppm-yr	28	RR	1.00	—
	>1.5–<5.5 ppm-yr	5	RR	0.46	0.18–1.20
	≥5.5 ppm-yr	15	RR	1.28	0.67.2.44
	*p*_trend_ > .5 (exposed)				
	*p*_trend_ > .5 (exposed and unexposed)				
Cumulative number of peaks ≥4.0 ppm	No association. Results not shown.				
Duration of Employment	No association. Results not shown.				

**Table 13b:** Other cohorts.

Reference	Obs	Estimate	95% CI	
Edling et al., 1987	2	SPIR*	4.00	0.50-14.40
Ott et al., 1989†	1	OR	1.00	—
Hayes et al., 1990	20	PMR	1.37	0.84-2.12
Dell and Teta, 1995	5	SMR	2.62	0.85-6.11
Stellman et al., 1998	4	RR	0.74	0.27-2.02
Coggon et al., 2003	15	SMR	0.86	0.48-1.41
Hauptmann et al., 2009	NR	OR	1.40	0.50-5.60

* Notes: *Standardized proportionate incidence ratio.

† Case-control study.

### 4.3. HBWoE evaluation of epidemiology studies

We conducted an HBWoE evaluation of the epidemiology data with regard to an association between formaldehyde exposure and leukemia. Based on review of the data discussed in the previous section, we address the following questions:

1. What are the implications of studies of individual lymphohematopoietic cancers and several group ings of these cancer types (e.g., all cancers of lymphoid origin, all of non-lymphoid origin) regarding leukemia risks from formaldehyde exposure?
2. Were results from the epidemiology data consistent for different types of exposure metrics (e.g., peak exposure, number of peak exposures ≥4.0 ppm, cumulative exposure)? Were results dependent on the robustness of exposure measurements, particularly for the NCI industrial worker and embalmer cohorts?
3. Were co-exposures considered in the interpretation of the study results?
4. Were there consistent exposure-response associa tions within and across studies?
5. Were there potential statistical limitations among the epidemiology studies?
6. How should latency be considered when interpreting study results? Is it possible that risks decline over time owing to a relatively short induction-incubation period (as proposed by Beane Freeman et al., 2009)?

As a whole, considering these questions allows for an assessment of the extent to which the epidemiology data support either a causal association between formaldehyde exposure and leukemia or an alternative hypothesis. Importantly, one needs to consider the epidemiology data in the context of the hematotoxicity and mode-of-action data (discussed later), as each of the three lines of evidence inform interpretation of the other; specifically, a claim of causation cannot be solely based on one or the other but has to be reflected consistently across the epidemiology, mode-of-action, and hematoxicity data.

#### 4.3.1. Cancer outcome assessments likely led to disease misclassification

There are several ways in which cancer outcomes were defined and assessed in the formaldehyde epidemiology studies, several of which may have led to disease misclassification and/or misleading results.

With few exceptions, most studies assessed cancer mortality, and several of the larger studies relied on death certificates to determine cause of death (e.g., Beane Freeman et al., 2009; Hauptmann et al., 2003, 2009; Hayes et al., 1990; Pinkerton et al., 2004; Stroup et al., 1986; Walrath and Fraumeni, 1983, 1984). Death certificates do not always identify leukemia subtype, and leukemia diagnosis was considered unreliable prior to 1992 (Collins and Lineker, 2004; Bachand et al., 2010; Miller et al., 1992; Percy et al., 1981, 1990, both as cited by Collins and Lineker, 2004). Thus, relying on death certificates may have led to disease misclassification.

In addition, diagnoses of lymphohematopoietic cancers has evolved in recent decades, and historic records may be inaccurate (Bachand et al., 2010; Collins and Lineker, 2004; Miller et al., 1992; Percy et al., 1990, as cited by Collins and Lineker, 2004; Scott and Chiu, 2006). For example, past classifications of lymphomas do not make distinctions between different cell types (Scott and Chiu, 2006). This means that, within studies that investigated subjects over many decades, individuals assigned the same cancer actually may not have had the same cancer.

There is also an issue with assessing cancers in categories. Each different kind of lymphohematopoietic cancer is a distinct disease with a unique etiology, set of risk factors, and, presumably, mechanism of action. Consequently, grouping cancer types together is not informative regarding risks for a particular cancer type. Any observed increased risks could be driven by risks for one cancer type (e.g., if the majority of cancers in a group were the same type, or one cancer type had very large risks associated with it); a lack of risks could be indicative of no risks among all lymphohematopoietic cancers or that combining cancer types masks true associations with one particular cancer type. For example, in the NCI industrial worker cohort, the two cancers that contribute to the association between peak formaldehyde exposure and all lymphohematopoietic cancers are multiple myeloma and Hodgkin's disease (Beane Freeman et al., 2009). These cancers are not associated with formaldehyde exposure in other studies.

In sum, disease misclassification likely led to uncertain risk estimates. In addition, studies that purport to show associations with a group of cancers that include leukemia do not provide sufficient evidence that risk, if it exists, is for leukemia and not another white cell cancer.

#### 4.3.2. Exposure assessments likely affected by exposure measurement error or misclassification

Because of the difficulty in obtaining exposure data for individuals in cohort and case-control studies, investigators typically estimated exposure from few, if any, measurements of formaldehyde concentrations. For example, Andjelkovich et al. (1995) assigned formaldehyde exposures to each iron foundry worker by job category based on midpoints of ranges from actual sampling data. Pinkerton et al. (2004) conducted analyses based on 1 year of measured data from the 1980s and applied it to the entire follow-up period in garment workers (1955-1998). Exposure estimates in formaldehyde workers in the NCI cohort were developed by assigning job categories from work histories abstracted in 1980 and an expert assessment of job and tasks using current and past measurement data (Stewart et al., 1986; Blair et al., 1986). Although this was considered to be a well-conducted exposure assessment for the time, validation of the exposure matrix was not possible, and exposures to formaldehyde and other potential confounders after 1980 were assumed to be minimal (Beane Freeman et al., 2009; Blair et al., 1986, 1990; Stewart et al., 1986). Peak exposure categories (none, >0 to <0.5 ppm, 0.5 to <2.0 ppm, 2.0 to <4.0 ppm, or ≥4.0 ppm) were estimated and defined as short-term exposures (generally less than 15 minutes) exceeding the 8-hour time-weighted average (TWA8) category (Blair et al., 1986; Beane Freeman et al., 2009). In the NCI embalmers cohort (Hauptmann et al., 2009), questionnaire data were linked to data from an exposure experiment (Stewart et al., 1992). No measurements of peak exposure were available, and average formaldehyde intensity, peak, time-weighted average, and cumulative exposure were estimated using a predictive model. Comparison of modeled average intensity to measurements from independent embalmings suggested the model overestimated exposure by 35%, and peak exposures could not be validated (Hauptmann et al., 2009).

Despite the paucity of exposure information, in two of the largest cohorts evaluated (the NCI industrial worker and embalmer cohorts), several exposure metrics were estimated (e.g., peak exposure, average exposure, cumulative exposure, exposure duration). Owing to the importance that peak exposures play in the interpretation of the NCI studies, it is important to note that peaks were not actually measured, but only inferred from job descriptions. Detailed analyses of exposure metrics used for the NCI industrial worker cohort were conducted by Blair et al. (1990) and Stewart et al. (1986). They reported that measures of duration (employment and exposure) and average exposure and level of exposure were highly correlated (*r*=.8). Peak exposures had low to moderate correlations with employment duration (*r* = .2), exposure duration (*r* = .3), cumulative exposure (*r*=.3), average exposure (*r*=.5), and level of exposure (*r* = .7). Average exposure showed little correlation with duration of employment (*r*=-.1) and duration of exposure (*r*=.0). Based on these correlations, it is unclear why lymphohematopoietic and leukemia mortality rates were associated with peak exposure but not with the number of peak exposures ≥4.0 ppm, cumulative exposure, or exposure duration (Beane Freeman et al., 2009). Even if higher exposure intensities are of more consequence as a result of formaldehyde's mode of action, those experiencing higher air concentrations over time with any repeatability would have a higher number of peak exposures ≥4.0 ppm and higher cumulative and average exposures, so these measures ought to show an association as well. This is not the case, indicating the association with peak exposure is not likely to be causal (other issues with this statistic are discussed below).

The lack of precise exposure data likely led to exposure measurement error and/or exposure misclassification in these epidemiology studies. This could have biased results either towards or away from the null (Jurek et al., 2005). Based on the null associations with other exposure metrics, in the case of peak exposure, it appears to be the latter.

#### 4.3.3. Exposures to other chemicals in the workplace may have confounded results

None of the studies adequately addressed co-exposures to other agents. For example, embalmers were exposed to infectious agents and other chemicals in embalming fluid, such as methanol, propylene glycol, industrial methylated spirit, phenol, and glycerol (Coleman and Kogan, 1998; Bachand et al., 2010; Bosetti et al., 2008; Collins et al., 2004). Industrial workers were likely exposed to other chemicals as well (e.g., antioxidants, asbestos, benzene, carbon black, dyes and pigments, hexamethylenetetramine, melamine, phenol, plasti-cizers, urea, and wood dust) (Beane Freeman et al., 2009). Although benzene is the only known leukemogen among these agents, it is possible that any observed risks, if found to be real, may have been attributable to exposures to other agents.

#### 4.3.4. Exposure-response associations within and among studies are not consistent

If formaldehyde is in fact a causal factor for leukemia, one would expect leukemia risk to increase with formaldehyde exposure both within and among studies. As described below, few studies actually assessed exposure-response (Pinkerton et al., 2004; Stern et al., 1987; Beane Freeman et al., 2009; Hauptmann et al., 2009); among those, consistent associations were not reported. Among studies, leukemia risks appeared to be higher in professionals with lower average formaldehyde exposures (mean TWA8 concentrations <0.5 to 1 ppm in professional settings [e.g., workplaces of histopathologists, embalmers, anatomists]; IARC, 2006), yet more highly exposed industrial workers (mean TWA8 concentrations <1 to >10 ppm in industrial settings [e.g., formaldehyde manufacturing]; IARC, 2006) showed lesser effects, adding to the weight of evidence suggesting formaldehyde is not a causal factor.

In analyses of formaldehyde and risks of all leukemias combined, Pinkerton et al. (2004) found no exposure-response associations with duration of exposure, time since first exposure, or year of first exposure in garment workers. Stern et al. (1987) also found no trend with duration of exposure. There were some statistically significant trends reported in the NCI industrial cohort but, as described below, these findings were not robust or indicative of causation.

**Figure 1:**
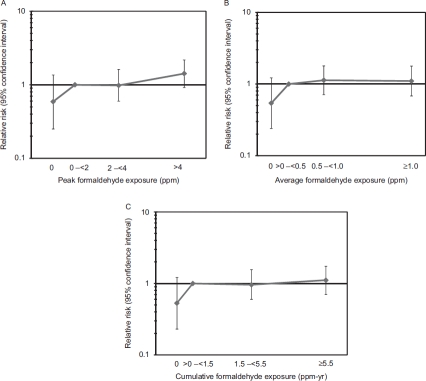
Relative risks of leukemia in the NCI industrial worker cohort compared to study subjects in the low-exposure category for (a) peak, (b) average, and (c) cumulative formaldehyde exposure. The “no exposure” category is comprised of workers from facilities that were presumably unexposed. The only statistically significant trends were for peak exposure when the “no exposure” workers were included in the analyses; all other trend tests including or excluding the “no exposure” workers were null. (See colour version of this figure online at www.informahealthcare.com/txc)

In the NCI industrial worker cohort, study subjects were divided into “low” (>0 to <2.0 ppm), “medium” (2.0 to <4.0), and “high” (≥4.0) exposure categories for the inferred lifetime peak level, and analyses were conducted by comparing risks in the medium- and high-exposure categories to those in the low-exposure category. As shown in Figure 1, these analyses showed no statistically significant associations and no exposure-response relationship with leukemia. If, however, a “zero” category was added, comprised of workers from the facilities that were presumably unexposed, the exposure-response trend for leukemia vs. “peak” became statistically significant, as was the contrast between the high vs. the zero (but not vs. the low) category.

Even though the “low” group included people down to zero as the lifetime “peak” exposure, the leukemia risk for the “zero” group was markedly lower. People classified as “zero” must have had systematically different job descriptions than those in the “low” category (for which peak exposure could be as low as zero and still admit them into the “low” group), so the comparability of these groups is in question. Moreover, the “zero” group has leukemia risks that are notably smaller than the general population. Indeed, when analyses were done on an SMR basis, risks, although not statistically significant, were much lower than those for the US population (SMR_all_ _leukemia_ = 0.48, 95% CI: 0.23–1.01; SMR_lymphatic_ _leukemia_ = 0.26, 95% CI: 0.04–1.82; SMR_myeloid_ _leukemia_ = 0.65, 95% CI: 0.25–1.74) (Beane Freeman et al., 2009). In contrast, among the all of the exposed groups, the SMR estimates are more consistent with US expected levels (SMR_all leukemia_ = 1.02, 95% CI: 0.85–1.22; SMR_lymphatic leukemia_ = 1.15, 95% CI: 0.83–1.59; SMR_myeloid leukemia_= 0.90; 95% CI: 0.67–1.21) (Beane Freeman et al., 2009).

In short, it appears that the reported significant relation of “peak” formaldehyde exposure and leukemia risk depended entirely on a lower-than-usual leukemia rate in the “zero” group rather than any effects among exposed people. “Peaks” were inferred possibilities rather than actual exposures, and they did not account for the duration of time spent in the highest peak-exposure category or the relevant latent period between the date of first highest peak exposure and death (Marsh and Youk, 2004). Individuals with high peak exposures early but not later in their career and those with low peak exposures for the majority of their career but high peaks near the end were likely in the same exposure category. Similarly, those with one peak exposure were likely classified in the same category as those with several peak exposures. In both of these scenarios, individuals with very different exposures were grouped in similar categories. In contrast, when grouping workers by the inferred number of peaks .4.0, or by cumulative or average exposure, individuals with similar exposures were more likely to be grouped together. Based on these latter metrics, formaldehyde exposure was not associated with leukemia. Thus, the finding of a significant effect for leukemia in the industrial cohort with peak exposure (Beane Freeman et al., 2009), which relies on picking apparently positive results among several similar analyses demonstrating inconsistent results, is not a substantive or compelling finding.

It is notable that in analyses limited to myeloid leukemia, a different pattern emerges. Neither Beane Freeman et al. (2009) nor Pinkerton et al. (2004) found any consistent exposure-response associations among the NCI industrial worker cohort and garment workers, respectively, regardless of the exposure metric, whereas Hauptmann et al. (2009) reported trends in embalmers based on peak exposure and duration of exposure, but only when the referent categories (<500 embalmings) were included in the analyses. No trends were observed when the referent category was excluded or when exposure was defined as average intensity, cumulative exposure, number of embalmings, or 8-hour TWA intensity. Again, this lack of consistency suggests that observed trends are not likely indicative of causation.

Regarding other lymphohematopoietic cancer types or groups, Beane Freeman et al. (2009) observed a trend only with peak exposure and all lymphohematopoietic cancers combined in the NCI industrial cohort. No other trends with all lymphohematopoietic cancers combined were observed in this cohort or the NCI embalmers cohort (Hauptmann et al., 2009). No trends were observed in either cohort for cancers of lymphoid origin and, for cancers of non-lymphoid origin, trends were only observed for duration of exposure in the NCI embalmers cohort when unexposed individuals were excluded (which left two individuals in the lowest category). There were no trends in the NCI industrial workers cohort with lymphatic leukemia, and no trends at all for non-Hodgkin's lymphoma or multiple myeloma. Beane Freeman et al. (2009) observed a trend with peak exposure and average intensity for Hodgkin's lymphoma but not with cumulative exposure, cumulative number of peaks ≥4.0 ppm, or duration of employment.

Regarding exposure-response among studies, professionals—such as embalmers, pathologists, and anatomists—have much lower formaldehyde exposures than industrial workers. Yet Blair et al. (1990) found small excess leukemia risks among professionals but not industrial workers; several meta-analyses have reported similar findings (Bosetti et al., 2008; Collins and Lineker, 2004). Some possible explanations have been put forth to explain these findings, including infectious agents, other chemicals in embalming fluid, occupational and lifestyle factors, observer bias, and a higher degree of scrutiny and medical attention owing to perceived risks (Coleman and Kogan, 1998; Bachand et al., 2010; Bosetti et al., 2008; Collins et al., 2004). Zhang et al. (2010a) suggest that effects in industrial workers may not be observed because analyses were conducted based on combined exposure categories, but this does not explain why effects are observed in professionals and not in industrial workers (who have higher exposures), and results from the few large studies that examined exposure-response associations do not support the hypothesis that formaldehyde is causally associated with leukemia.

Overall, although some statistically significant trends have been noted, these trends were not found consistently within or among studies. The lack of consistent exposure-response associations within or among studies indicates that the few associations noted between formaldehyde and leukemia are not causal.

#### 4.3.5. Statistical limitations may have led to spurious associations

When the same set of data is analyzed in multiple parallel ways using different models, groupings, or summary measures, the meaning of statistical tests becomes distorted by the multiple-comparisons problem. That is, if enough alternatives are tried, some might be “significant” by chance alone (since, at a criterion of *p=* .05, even when there is no effect, 5% of comparisons are ruled “significant”).

Data from several studies, including those of the NCI industrial worker and embalmer cohorts, were analyzed many different parallel ways (e.g., average or cumulative or peak exposure; pairwise comparisons or trends; using internal or external controls; with or without a “unexposed” group in the trend test; individual tumor types or various measures of combined tumors). Unless a correction for multiple comparisons is made, finding marginal significance in one or a few such comparisons is not surprising even when there is no true effect. For example, in the NCI industrial worker cohort, associations were reported with peak exposures, but there was no a priori reason to focus on peak exposures. Furthermore, associations were not found for other, more accurate indicators of exposure, such as the number of peak exposures ≥4.0 ppm, cumulative exposure, and average exposure. If there is no a priori reason to choose a superior exposure metric, one should not select a model based solely on statistical performance because choosing the metric with the strongest association with outcome could lead to bias (Kriebel et al., 2007). Instead, one should choose a model based on which is most consistent with the hypothesized mechanism of action. In the case of formaldehyde, peak exposure is clearly an inferior metric (discussed above), and this provides an even stronger argument for not choosing a model based on the strongest statistical association. The result for peaks can at most be a hypothesis-generating observation to be tested on future data. Otherwise, it is post hoc and arbitrary.

#### 4.3.6. The latency argument proposed by Beane Freeman et al. (2009) appears to be a post hoc explanation for the observed effects

Epidemiology studies are often limited in that they are not conducted over long enough periods of time for cancer from particular exposures to develop, in which case causal associations cannotbe detected. In the case of leukemia, Beane Freeman et al. (2009) suggest just the opposite, stating that risks for myeloid leukemia may decline over time owing to a relatively short induction-incubation period. In other words, they suggest that, after a latent period during which risks are not increased, increased risks for leukemia will be observed within a certain time period and plateau afterwards. Because of this, they suggest that risks may diminish or not be observed if a study has too long a follow-up period. This appears to be a post hoc explanation for the diminished risks associated with peak exposure observed in the NCI industrial worker cohort with follow-up through 2004 vs. 1994 (in workers who died 16-25 years after the first exposure). It does not explain how risks were only observed with peak exposure, and not with other exposure metrics (particularly cumulative number of peaks ≥4.0 ppm), or how this trend was not observed in the NCI embalmers cohort (Hauptmann et al., 2003; Walrath and Fraumeni, 1984) or garment workers cohort (Pinkerton et al., 2004), in which risks were only observed with exposures >20 years. Also, Beane Freeman et al. (2009) did not test this hypothesis even though they had the data to do so. For all of these reasons, a shorter latency is not a scientifically valid explanation for the lack of observed risks by Beane Freeman et al. (2009).

#### 4.3.7. Recent formaldehyde meta-analyses do not support an association between formaldehyde exposure and leukemia

A number of recent meta-analyses have been conducted on the body of epidemiology studies concerning formaldehyde and leukemia (Bachand et al., 2009; Zhang et al., 2009; Bosetti et al., 2008; Collins and Lineker, 2004; Schwilk et al., 2010). Only the most recent of these analyses (Bachand et al., 2009; Schwilk et al., 2010) include the most recent update to the NCI industrial worker cohort; the others rely on the Hauptmann et al. (2003) analysis, in which 1006 deaths were omitted unintentionally. Of the five meta-analyses, three reported no overall association between formaldehyde and leukemia (Bachand et al., 2009; Bosetti et al., 2008; Collins and Lineker, 2004), and methodological limitations of the other two meta-analyses (Zhang et al., 2009; Schwilk et al., 2010), which are almost the same except for the addition of two studies in the latter, make it challenging to interpret their summary risk estimates (REs).

Bachand et al. (2009) found that, among cohort studies, REs for exposed vs. unexposed ranged from 0.43 to 1.60 for leukemia—none were statistically significant (i.e., no 95% CI excluded 1.0)—and the summary risk estimate indicated no association (RE=1.05, 95% CI: 0.91-1.20). Data from only two case-control studies were analyzed, neither of which reported increased risks (Blair et al. [2001]: RE = 0.98, 95% CI: 0.70-1.36; Partanen et al. [1993]: RE= 1.40,95% CI: 0.25-7.91). No associations were observed when analyses were stratified by leukemia type (myeloid, lymphatic/lymphocytic, or other/unspecified), job type (professional/technical or industrial), or region (USA/Canada or Europe).

Bosetti et al. (2008) calculated REs for lymphohe-matopoietic cancers and leukemia among professionals and industrial workers evaluated in cohort studies published through February 2007. Risks of lymphohe-matopoietic cancers among professionals were increased (RE=1.31, 95% CI: 1.16-1.48), but they were decreased among industrial workers (RE = 0.85, 95% CI: 0.74-0.96). Similarly, leukemia risks were elevated among professionals (RE= 1.39, 95% CI: 1.15-1.68), but not industrial workers (RE = 0.90, 95% CI: 0.75-1.07). Based on analyses of 18 studies published through December 1, 2003, Collins and Lineker (2004) found similar results. They reported that leukemia risks were not increased among industrial workers (RE = 0.9, 95% CI: 0.8-1.0) or patholo-gists and anatomists (RE= 1.4, 95% CI: 1.0-1.9), butwere increased among embalmers (RE = 1.6, 95% CI: 1.2-2.0), who had among the lowest exposures.

In contrast to the three meta-analyses discussed above, Zhang et al. (2009) found a significant effect across industries (RE=1.54, 95% CI: 1.18-2.00). This can be explained by the unusual means of selecting and combining studies: they used different measures of exposure, selecting only one from each study even if several were examined, resulting in selection of peak exposure for studies where available, then average exposure and cumulative exposure for others, and, finally, exposure duration if none of the other metrics were analyzed. Zhang et al. (2009) claim this is because average and cumulative exposure maybe less accurate measures of true exposure if workers with very high exposure also have long intervening periods with little or no exposure, but they have not considered whether these metrics are relevant for assessing risk. Moreover, if several categories or levels of exposure were examined, Zhang et al. (2009) took data from only the highest among them. What constituted a “high” category also varied considerably among studies, depending on how each study established gradations of exposure. As a consequence, the comparisons across studies are very heterogeneous, and it is not clear whether a comparable question was being examined in each case. Furthermore, by not using the entire range of exposure estimates (i.e., by examining risks in the high-exposure group vs. the low-exposure group only), exposure-response could not be assessed, which likely generated misleading results, since a lack of exposure-response can indicate a lack of a causal association. Finally, Zhang et al. (2009) did not use the most recent NCI industrial worker cohort data, instead relying on data from the Hauptmann et al. (2003) study, which didn't account for over 1000 deaths in the cohort and only reported increased risks of myeloid leukemia based on internal comparisons that depended on the reference category and category cut points.

Schwilk et al. (2010) updated the Zhang et al. (2009) analysis by including the most recent NCI industrial worker and embalmer cohort studies (Hauptmann et al., 2009; Beane Freeman et al., 2009) and reported increased risks of leukemia (RR=1.53, 95% CI: 1.11-2.21) and myeloid leukemia (RR = 2.47, 95% CI: 1.42-2.47). Because Schwilk et al. (2010) use similar methods as Zhang et al. (2009), their study suffers from the same limitations. In addition, Schwilk et al. (2010) use one-sided *p* values, which increased the likelihood of false-positive results. They also reported several exposure-response relationships in six studies and concluded an exposure-response association exists, but they did not discuss these associations for all exposure metrics from each study. As we have shown, had they done this, it would be evident that there are no consistent exposure-response associations between formaldehyde exposure and leukemia. Because of all these limitations, the results of the Zhang et al. (2009) and Schwilk et al. (2010) analyses should be interpreted with caution, especially in view of the substantial heterogeneity and their lack of concordance with other meta-analyses.

Overall, results from the meta-analyses of formaldehyde and leukemia are consistent with a lack of association and the results of our weight-of-evidence evaluation.

### 4.4. Summary

As a whole, the available formaldehyde epidemiology studies do not support a causal association between formaldehyde exposure and leukemia. As demonstrated in the endpoint-by-endpoint analysis and Tables 4 to 13, there is no lymphohematopoietic cancer or group of lymphohematopoietic cancers for which associations with formaldehyde were found consistently within or across studies. Although some statistically significant associations were reported, these were outnumbered by null findings in the more robust studies using related exposure metrics, and there were no consistent exposure-response relationships observed. Limitations in exposure and cancer outcome assessments as well as statistical analyses also likely affected calculations of risk.

If formaldehyde were truly a causal factor for leukemia, consistent observations of effect should have been observed, with increased risks found with increased exposures. Because this is not the case, it is most likely that any observed effects were a result of confounders, limitations in statistical methods (e.g., multiple comparisons), disease misclassification, and/or exposure misclassification/measurement error.

This question can be further explored by considering information on toxicology and mode-of-action studies. It is a precept of the HBWoE approach that one considers the cross-discipline integration of hypothesized effects. To the degree that consideration of animal data and dosimetry casts doubt on the ability of inhaled formaldehyde to interact with and perturb hematopoiesis, this increases the relative plausibility of a conclusion that those associations seen in purely epidemiologic investigations are not in fact causal but are the result of chance, co-exposures, or confounding, compared to an analysis that relies solely on the observed patterns seen among the epidemiology studies themselves. Moreover, if the dependence of effect on peak formaldehyde exposures that has been suggested in some epidemiology studies is indeed important in understanding the patterns among human studies, this dependence ought to be reflected in information about dosimetry and hypothesized modes of action. Conversely, if hypothesized modes of action, if operating, would not be expected to produce a dependence on peak exposures, then the role of peaks in explaining positive and null results among human studies is weakened. These issues will be discussed further below.

## 5. Weight of evidence regarding hematotoxicity from formaldehyde exposure

In the following analysis, we examine a potential association between formaldehyde exposure and leukemia in animals and hematotoxicity as reflected by changes in peripheral blood hematology in both humans and animals. We conducted a literature search, using PubMed, for all human studies measuring or estimating formaldehyde exposure and the changes in peripheral blood hematology, in addition to all short- and long-term animal studies that investigated either potential formaldehyde-associated leukemogenicity or hematology changes in peripheral blood. Search terms included “hematology,” “hematotoxicity,” “leukemia,” “lymphoma,” “lymphohe-matopoietic,” “formaldehyde,” “rat,” “mouse,” “rodent,” “human,” and “occupational.” We also relied on references within the papers that we found in the PubMed search and on non-peer-reviewed analyses of animal studies, which are part of the current debate on potential formaldehyde leukemogenicity.

Hematotoxicity may be defined as an insult that can be identified in blood and blood components. This tox-icity is reflected in the production or loss of blood components, including red blood cells (RBCs, erythrocytes), white blood cells (WBCs, leukocytes), platelets, and hemoglobin (Hb) (found in RBCs), responsible for carrying oxygen. Hematopoietic progenitor cells (HPCs) in bone marrow give rise to RBCs and WBC subtypes— neutrophils, lymphocytes (B and T types), monocytes, eosinophils, basophils, and megakaryocytes, from which platelets are derived (Cotran et al., 1999). There are three main types of myeloid progenitor cells that undergo several stages of differentiation to give rise to the blood cells (e.g., granulocyte-macrophage colony-forming unit [CFU-GM] gives rise to granulocytes and macrophages) (Cotran et al., 1999). Blood-forming cells normally leave the bone marrow only when fully differentiated, but a small number of progenitor cells can leave the bone marrow and circulate in blood (Aster and Kumar, 1999).

A decline in peripheral blood of one or more WBC type counts can result in leukopenia, that of RBCs, anemia, and when all cell types in peripheral blood decline, pancytopenia. When the oxygen-carrying capacity of RBCs is compromised, new RBCs can be manufactured at a faster-than-usual rate, which may result in larger mean RBC size (mean corpuscular volume, MCV). Numerous factors can influence changes in blood components. These include, but are not limited to, certain infections, nutrient imbalance, xenobiotic insults to either blood components directly or to the bone marrow progenitor cells, alcohol intake, smoking, excessive bleeding, menstruation, and certain medications (Mayo Clinic, 2008). Leukemogenesis may be viewed as a multistage process that involves interruption of the normal cellular differentiation process in the bone marrow and accumulation of the undifferentiated cells in bone marrow, a condition that crowds and suppresses the remaining normal hematopoietic progenitor cells. This suppression of normal hematopoiesis can result in anemia, leukopenia, and pancytopenia. Eventually, the undifferentiated, and abnormally functional, cells in bone marrow spill into peripheral blood and become the predominant cells there (Irons and Stillman, 1996; Aster and Kumar, 1999). As discussed in Section 5.3.3 below, most known leukemogens can cause pancytopenia (a decline in all cell types in peripheral blood) that is secondary to bone marrow toxicity. In addition to pancytopenia, bone marrow toxicity has been associated with decreased counts or viability of circulating blood cells, including progenitor cells (Dempster and Snyder, 1991; Toft et al., 1982, both as cited in ATSDR 2007).

### 5.1. Formaldehyde hematotoxicity in animals

#### 5.1.1. Hematology

Several animal studies have assessed the hematotoxic potential of formaldehyde via both oral and inhalation routes. As shown in Table 14, these studies ranged from subacute to chronic in duration (4 weeks to 24 months) and used a wide range of exposure concentrations; the highest was 5000 ppm in drinking water (Tobe et al., 1989) and 20 ppm in air (Woutersen et al., 1987).

**Table 14:** Formaldehyde animal hematotoxicity studies.

			Exposure		Outcomes	
				
Study	Species	Sex	Concentration	Duration	RBC, Hct, Hb	WBC
Inhalation studies						
Monticello et al., 1989	Monkey, rhesus	Male	0, 6 ppm	6 h/d, 5 d/wk, 6 wk	NS	NS
Appelman et al., 1988	Rat, Wistar	Male	0, 0.1, 1, 10 ppm	6 h/d, 5 d/wk, 13 or 52 wk	NS	NS
Holmstrom et al., 1989	Rat, Sprague-Dawley	Female	0, 12.6 ppm	6 h/d, 5 d/wk, 22 mo	NS	NS
Kerns et al., 1983	Rat, Fischer 344	Female, Male	0, 2.0, 5.6, 14.3 ppm	6 h/d, 5 d/wk, 24 mo with follow-up till 30 mo	NS	NS
As above	Mouse, B6C3F1	Female, Male	As above	As above	NS	NS
Dean et al., 1984	Mouse, B6C3F1	Female	0, 15 ppm	6 h/d, 5 d/wk, 3 wk	NS	↓monocytes Other cell types NS
Kamata et al., 1997	Rat, Fischer 344	Male	0, 0.3, 2, 15 ppm	6 h/d, 5 d/wk, 28 mo	NS	NS
Woutersen et al., 1987	Rat, Wistar	Female, Male	0, 1, 10, 20 ppm	6 h/d, 5 d/wk, 13 wk	NS	NS
Ingestion Studies						
Vargova et al., 1993	Rat, Wistar	Male	0, 20, 40, and 80 mg/kg bw/d	5 d/wk, 4 wk; gastric intubation	↑ at 40, 80 mg/kg/d	↑monocytes ↑lymphocytes
Til et al., 1989	Rat, Wistar	Female	0, 1.8, 21, 109 mg/kg bw/d	Daily, 24 mo, drinking water	NS	NS
As above	Rat, Wistar	Male	0, 1.2, 15 or 82 mg/kg bw/d	Daily, 24 mo, drinking water	NS	NS
Til et al., 1988	Rat, Wistar	Female, Male	5, 25 and 125 mg/kg bw/d	Daily, 4 wk	NS	NS
Johannsen et al., 1986	Rat, Sprague-Dawley	Female, Male	0, 50, 100, 150 mg/kg/d	Daily, 3 mo	NS	NS
As above	Dog, Beagle	Female, Male	0, 50, 75, 100 mg/kg/d	Daily, 3 mo	NS	NS
Tobe et al., 1989	Rat, Wistar	Female, Male	0, 200, 1000, 5000 ppm (0, 10, 50, 300 mg/kg bw/d)	Daily, 24 mo, drinking water	↓, not concentration dependent	Assessed, NR

*Note*. WBC = white blood cell count in peripheral blood; RBC = red blood cell count in peripheral blood; Hct = hematocrit; Hb = hemoglobin concentration in blood; NS = not statistically significant;

↓ statistically significant decrease;

↑ statistically significant increase; mg/kg bw/d = milligram per kilogram body weight per day; ppm = parts per million; wk = week(s); mo = month(s).

As shown in Table 14, the results from the inhalation studies generally show that formaldehyde does not induce changes in standard hematology parameters in peripheral blood. One study (Dean et al., 1984) showed a significant (*p* < .05) decrease in monocytes, but not other leukocytes, in the blood of B6C3F1 mice after 3 weeks of exposure to 15 ppm formaldehyde, but no exposure-related changes in either bone marrow cellularity or CFU progenitor cell counts. In contrast, a *longer-term* study by Kerns et al. (1983) in the same mouse strain found no changes in hematology.

The results of the ingestion exposure studies are generally not indicative of a hematotoxic effect of formaldehyde. For example, the studies by Appelman et al. (1988), Johannsen et al. (1986), and Til et al. (1988, 1989) did not find any changes in hematology with exposure. Tobe et al. (1989) reported statistically significant lowered RBC counts and Hb concentrations, but these changes were not exposure-concentration dependent. The results by Tobe et al. (1989) were contradicted by Vargova et al. (1993), who reported increased hematocrit (Hct) and Hb concentrations and RBC counts in blood. Furthermore, statistically significant changes in WBC counts following oral exposure to formaldehyde were found by Vargova et al. (1993) as increased monocyte counts and decreased lymphocyte counts only following exposure to very high doses (as high as 80mg/kg for 4 weeks, equivalent to ∼800 ppm in drinking water).

#### 5.1.2. Leukemia

We reviewed eight animal studies investigating the tumorigenic potential of formaldehyde by inhalation (Kerns et al., 1983; Kamata et al., 1997; Albert et al., 1982; Feron et al., 1988) and ingestion (Tobe et al., 1989; Til et al., 1989; Takahashi et al., 1986; Soffritti et al., 1989, 2002). We also considered two unpublished and non-peer-reviewed analyses (DeVoney et al., 2010, poster abstract only; Woutersen, 2007) of data from the Battelle Columbus Laboratories (1981) study that was later published by Kerns et al. (1983). Animal inhalation studies generally showed significantly increased rates of nasal tumors (Kerns et al., 1983; Kamata et al., 1997; Sellakumar et al., 1985) but, as shown in Table 15, not of leukemias or lymphomas, when these endpoints were investigated. Ingestion studies showed neither increased rates of nasal nor hematopoietic malignancies in rats. We have limited our review to analyzing potential formaldehyde-associated changes in leukemia and lymphoma rates in the exposed animals. Table 15 describes the animal species, exposure characteristics, and hematopoietic malignancy outcomes of the studies discussed in this section.

**Table 15:** Formaldehyde animal carcinogenicity studies.

			Exposure		Tumor rate vs. control (%)	Background tumor rate
Study	Species	Sex	Concentration	Duration	Hematopoietic malignancies	Mean (range)
Inhalation studies						
Albert et al., 1982	Rat, Sprague-Dawley	Male	0, 14.7 ppm	6 h/d, 5 d/wk, 19.4 mo	NR (Authors performed complete necropsy and histological sections taken from organs with gross pathological alterations)	
As above	As above	Male	As above	As above	NS	
Kerns et al., 1983	Rat, Fischer 344	Female	0, 2.0, 5.6, 14.3 ppm	6 h/d, 5 d/wk, 24 mo with follow-up till 30 mo	NS	
As above	As above	Male	As above	As above	NS	
As above	Mouse, B6C3F1	Female	As above	As above	NS	
As above	As above	Male	As above	As above	NS	
DeVoney et al., 2006 poster, 2010 poster abstract (analysis of Battelle Columbus Laboratories [1981])*	Rat, Fischer 344	Female	0, 2.0, 5.6, 14.3 ppm	6 h/d, 5 d/wk, 24 mo with follow-up till 30 mo	Leukemia; 24% vs. 15% (14.3 ppm) (no statistical test identified)	Leukemia; 37.3% (24%–54%)†
As above	As above	Male	As above	As above	NS	
As above	Mouse, B6C3F1	Female	As above	As above	Lymphoma; 28% vs. 18% (14.3 ppm) (no statistical test identified)	Lymphoma; 19.9% (6%–44%)†
As above	As above	Male	As above	As above	NS	
Woutersen, 2007* (analysis of Battelle Columbus Laboratories [1981])	Rat, Fischer 344	Female	0, 2.0, 5.6, 14.3 ppm	6 h/d, 5 d/wk, 24 mo with follow-up till 30 mo	NS	
As above	As above	Male	As above	As above	NS	
As above	Mouse, B6C3F1	Female	As above	6 h/d, 5 d/wk, 24 mo with follow-up till 27 mo	Lymphoma; considered data immediately after 24-month exposure: 17%, 16%, 9%, 29% (0, 2.0, 5.6, 14.3 ppm, respectively);considered data 3 months after 24-month exposure: 50%, 20%, 15%, 45% (0, 2.0, 5.6, 14.3 ppm, respectively)	Lymphoma; 19.9% (6%–44%)†
As above	As above	Male	As above	As above	NS	
Sellakumar et al., 1985 (extended analysis of the study by Albert et al. [1982]).	Rat, Sprague-Dawley	Male	0, 14.8 ppm (with or without ∼10 ppm HCl)	6 h/d, 5 d/wk, >28 mo	NS	
Kamata et al., 1997	Rat, Fischer 344	Male	0, 0.3, 2, and 15 ppm	6 h/d, 5 d/wk, 28 mo	NR (No hematological changes were found. Also, the authors examined femur, mesenteric lymph nodes, many other organs, and “any other gross lesions”	
Feron et al., 1988	Rat, Wistar	Male	0, 10, 20 ppm	6 h/d, 5 d/wk, (4, 8 or 13 wk); follow-up >28 mo	NR (Animals were autopsied and examined for gross pathological changes. The authors found no gross pathological changes)	
Ingestion studies						
Til et al., 1989	Rat, Wistar	Female	0, 1.8, 21, 109 mg/kg bw/d (0, 20, 260, 1900 ppm)	Daily, 24 mo, drinking water	NS	
As above	Rat, Wistar	Male	0, 1.2, 15, 82 mg/kg bw/d (0, 20, 260, 1900 ppm)	As above	NS	
Soffritti et al., 1989	Rat, Sprague-Dawley	Female	Experiment 1: 0, 10, 50, 100, 500, 1000, 1500 (rats were 7 weeks old at start) Experiment 2: 0, 2500 ppm (rats were 25 week old breeder and their offspring)	Daily, 24 mo, drinking water with lifetime follow-up >36 mo	All hematopoietic malignancies Experiment 1: 14% vs. 3% Experiment 2: Breeders: 11.1% vs. 5% Offspring: 0% vs. 6.1%	Up to 19% in males and 14% in males and females combined‡
As above	Rat, Sprague-Dawley	Male	As above	As above	All hematopoietic malignancies Experiment 1: 22% vs. 4%) Experiment 2: Breeders: 11.1% vs. 0% Offspring: 11.1% vs. 5.1%	Up to 19% in males and 14% in males and females combined‡
Tobe et al., 1989	Rat, Wistar	Female	0, 200, 1000, 5000 ppm	Daily, 24 mo, drinking water	NR (No hematological changes were found. Also, authors examined lymph nodes and several other organs and “tumorous tissues”)	
As above	As above	Male	As above	As above	As above	
Takahashi et al., 1986	Rat, Wistar	Male	0, 5000 ppm	Daily, 32 wk after 8 wk exposure to *N*-methyl-*N*'-nitro-*N*-nitrosoguanidine (MNNG), drinking water	NR (Animals were necropsied and “no malignant tumors found outside the gastroduodenal tract”)	

*Note*. NR = not reported; NS = not statistically significant; NA = not applicable/available; mg/kg bw/d = milligram per kilogram body weight per day; ppm = parts per million; wk = week(s); mo = month(s).

* To adjust for early deaths, Woutersen (2007) used the Peto mortality prevalence trend test, and DeVoney (2006 poster, 2010 poster abstract) “adjusted for early deaths and time to tumor observation.” It should be noted that these two references are a conference presentation and a conference poster, respectively, and are not peer-reviewed publications.

† Data from Haseman et al. (1998); background tumor rates from NTP studies data based on spontaneous tumor rates in approximately 1000 animals.

‡ Data from the review by Feron et al. (1990) of background leukemia incidence in rats from the same colony used in the study by Soffritti et al. (1989).

Battelle Columbus Laboratories (1981) exposed Fischer 344 rats and B6C3F1 mice via inhalation to formaldehyde for 2 years. As shown in Table 15, tumor incidence data from Battelle Columbus Laboratories (1981) were analyzed by Kerns et al. (1983), DeVoney et al. (2010 poster abstract), and Woutersen (2007) but with different outcomes. Whereas DeVoney et al. (2010 poster abstract) reported elevated lymphoma incidence in female B6C3F1 mice and elevated leukemia incidence in female Fischer 344 rats, Woutersen's analysis found increased lymphoma incidence only in female mice, immediately after exposure, although this trend showed no statistically significant association for formaldehyde when the 3-month period following exposure was considered (Woutersen, 2007). Kerns et al. (1983) reported no formaldehyde-associated elevated rates of leukemia or lymphoma in this study in either rats or mice. It is noteworthy that the leukemias found in Fischer 344 rats by Battelle Columbus Laboratories (1981) likely included mononuclear cell leukemias (MCLs), which are usually observed in ∼50% and ∼28% of unexposed male and female Fischer 344 rats, respectively (Haseman et al., 1998). This high background incidence of MCLs in rats brings into question the outcomes and resulting conclusions of these non-peer-reviewed results by DeVoney et al. (2006 poster, 2010 poster abstract). Furthermore, as discussed by Ishmael and Dugard (2006), any MCL counterpart in humans is rare, MCLs are more likely elevated by chemical exposure in Fischer rats but not in Osborne Mendel or Sprague Dawley rats, and MCL incidence can be reduced by type of chemical delivery vehicle, such as corn oil, all which suggest that any positive findings involving MCL incidence in animals may not be relevant in humans.

Four studies, all of which used rats, assessed the tum-origenicity of formaldehyde from drinking water. The exposure concentrations were as high as 5000 ppm in water. Exposure durations were either 2 years (Soffritti et al., 1989, 2002; Til et al., 1989; Tobe et al., 1989) or 32 weeks preceded by 8 weeks of treatment with a tumor initiator, AT-methyl-AT-nitrosoguanidine (Takahashi et al., 1986). Of these studies, only Soffritti et al. (1989, 2002) reported statistically significantly increased hematopoietic malignancies (i.e., lymphomas and leukemias).

Soffritti et al. (1989) performed two experiments. In the first experiment, the authors exposed male and female Sprague Dawley rats to 0, 10, 50, 100, 500, 1000, or 1500 ppm of formaldehyde in drinking water for 2 years. The authors reported an increase in leukemia incidence at concentrations above 50 ppm (specifically, lymphoblastic leukemias and lymphosarcomas, immu-noblastic lymphosarcomas, and “other leukemias,” although the anatomic location of these neoplasms was not indicated). The increase, particularly in immu-noblastic lymphosarcomas, was not exposure related, however. Moreover, the lack of statistical analysis of the data in this report does not allow a full assessment of cause and effect. In the second experiment, the authors exposed male and female Sprague-Dawley breeder rats and their male and female offspring to regular drinking water and drinking water containing 2500 ppm formaldehyde. The authors reported increased leukemia rates (specifically immunoblastic lymphosarcomas and “other leukemias”) for each of the male and female breeder groups and the male offspring group, but there was no incidence in the female offspring group. The lack of statistical analysis for this experiment also precludes proper data assessment. In their subsequent report of this same study, Soffritti et al. (2002) presented the results from only the first experiment mentioned above, but these results differed from the earlier report by Soffritti et al. (1989). The authors neither explained why they included only one experiment in this report nor addressed the differences in reported outcomes between reports.

### 5.2. Formaldehyde hematotoxicity in humans

There are limited, mostly occupational, studies in humans of the hematotoxic effects of exposure to formaldehyde. Tang et al. (2009) recently abstracted data from eight studies conducted in China that assessed WBC and platelet counts and Hb concentration in subjects occupa-tionally exposed to formaldehyde (Yang et al., 2007; Kuo et al., 1997; Cheng et al., 2004; Xu et al., 2007; Qian et al., 1988; Feng et al., 1996; Tang et al., 2003; Tong et al., 2007, all as cited by Tang et al., 2009). The findings by Kuo et al. (1997), the only study of hematological effects cited by Tang et al. (2009) available in English, are associated with several uncertainties that weaken the conclusions drawn by Tang et al. (2009) about these effects. Many questions arise about the outcomes and exposure-related uncertainties in the findings of the other, untranslated, studies cited by Tang et al. (2009) (Section 5.3.2 provides further discussion of this point). We found four other studies that assessed hematological parameters associated with formaldehyde exposure (Ye et al., 2005; Lyapina et al., 2004; Srivastava et al., 1992; Zhang et al., 2010b). Table 16 describes the exposure characteristics and hematology outcomes, as available, of the studies discussed in this section.

**Table 16:** Human hematotoxicity studies.

				Peripheral blood changes			
Study	Exposure (ppm)	Number of persons (exposed, control)	% change in WBC count between groups or % of subjects with decreased hematology parameters	Total WBC	Platelets	Hb	Other findings
Yang et al., 2007*	0.018–0.036	239, 200	WBCs: 14% (E) vs. 4% (C) Platelets: 11% (E) vs. 1% (C) RBCs: 32% (E) vs. 21.5% (C)	↓↓	↓↓	↓↓	
Kuo et al., 1997	ND–0.054 (personal samples) 0.006–0.237(area samples)	50, 71	NA	↓	NS	NS	Statistically significant (-0.33, *p* < .05) inverse relationship between FA and WBC counts, but not 11 other hematology parameters
Cheng et al., 2004*	0.2.0.76	72, 150	WBCs: 14% in E vs. 5% in C	↓	NA	NA	
Lyapina et al., 2004	0.52–1.049 (mean, 0.71)	29, 21	NA	NS	NS	NR	Statistically significant inverse relationship between duration of exposure to formaldehyde and RBC counts and Hct
Xu et al., 2007*	0.36–5.56	10, 10	−11.4	NS	NS	NS	
Ye et al., 2005	0.8 (8-h TWA), 1.38 (max) in workers; vs. 0.009 (mean), 0.012 (max) in controls	36, 6	NA	NA	NA	NA	Workers vs. students in dorms. Statistically significantly increased B lymphocytes. Statistically significantly decreased CD3 and CD8 but not CD4 T lymphocytes in peripheral blood
As above	0.09 (5-h TWA), 0.24 (max); vs. 0.009 (mean), 0.012 (max) in controls	18, 6	NA	NA	NA	NA	Waiters vs. students in dorms. No change in B or T lymphocytes counts in peripheral blood
Zhang et al., 2010b	0.63–2.51 (mean, 1.28)	43, 51	−13.5	↓↓	↓	NS	Decreased RBC; increased MCV (statistically significant)
Qian et al., 1988*	2.44 (estimated)	55, 41	−13.3	↓↓↓	NA	NA	Increase in immunoglobulins (Ig) IgM and IgA, and eosinophils (no statistical significance reported)
Feng et al., 1996*	0.57–15.61	104, 68	NA	NS	NA	NS	
Srivastava et al., 1992	NR	6, 0	Increased blood lymphocyte counts in 3 of 6 subjects	NA	NA	Decrease	Decreased Hb in 4 of 6 subjects
Tang et al., 2003*	NR	110, 120	−17.1	NS	NA	NA	Decreased WBC count with increasing work years (no statistical significance reported)
Tong et al., 2007*	NR	65, 70	−18	↓↓↓	↓↓↓	NS	WBC and platelet counts decreased with increasing work years (no statistical significance reported)

*Note*. E = exposed group; C = control group; NR = not reported; NS = not statistically significant; NA = not applicable/available; WBC = white blood cell count in peripheral blood; RBC = red blood cell count in peripheral blood; Hct = hematocrit; Hb = hemoglobin content of RBCs;

↓ statistically significant decrease (*p* < .05);

↓↓ statistically significant decrease (*p* < .01);

↓↓↓ statistically significant decrease (*p* < .001).

* As cited in Tang et al. (2009). These studies are in Chinese and are not available on PubMed.

In the China-based hematotoxicity studies reported in Tang et al. (2009), the leukocyte counts in the exposed subjects were generally lower than in the control subjects, but differences were statistically significant in only four (Yang et al., 2007; Cheng et al., 2004; Qian et al., 1988; Tong et al., 2007, all as cited by Tang et al., 2009) of the eight studies. Feng et al. (1996, as cited by Tang et al., 2009), Xu et al. (2007, as cited by Tang et al., 2009), and Tang et al. (2003, as cited by Tang et al., 2009) did not find statistically significant differences in leukocyte counts related to formaldehyde exposure. Several studies found an inverse correlation between duration of exposure to formaldehyde and leukocyte counts (Tong et al., 2007; Yang et al., 2007; Tang et al., 2003; Kuo et al., 1997, all as cited by Tang et al., 2009), but the relationship was only reported as statistically significant in the study by Kuo et al. (1997, as cited by Tang et al., 2009).

Ye et al. (2005) assessed lymphocyte subset counts in peripheral blood in student controls (living in dorms), factory workers (8.6 years mean duration of exposure), and ballroom waiters (12 week exposure duration), all non-smokers. The formaldehyde concentration was 0.8 ppm in the factory, 0.09 ppm in the ballroom, and 0.009 ppm in the dorms. As shown in Table 16, differences in percentage of lymphocyte subset counts among groups were limited to statistically significantly increased B lymphocytes, and decreased CD3 (total T cells) and CD8 (T-cytotoxic), but not CD4 (T-helper-inducer), T lymphocytes, in the workers as compared with the students. The change in CD3 cells (∼5% decrease) appeared to be driven by CD8 cells (∼25% decrease). It is not apparent why CD3 and CD8 cells decrease, but not CD4. Since total T cells were decreased, however, it is possible that there was no need for the helper T cells (CD4) and therefore no change was observed relative to controls. Nevertheless, this is an interesting finding by Ye et al. (2005) and warrants further investigation of lymphocyte subset dynamics. Further, since this study shows statistically significant changes in both B- and T-cell populations, it is likely that any effect attributed to formaldehyde exposure is immune and acquired and did not originate from an insult to the bone marrow. It would be interesting to know the counts in peripheral blood of RBCs and WBCs, other than lymphocytes, to shed light on potential bone marrow involvement, but these data were not available for this study.

Lyapina et al. (2004) found no significant differences in standard hematology tests of workers applying formaldehyde-carbamide glue when compared with those from subjects with no known appreciable formaldehyde exposure (formaldehyde levels not reported for either group). The authors reported a statistically significant inverse relationship, however, between the duration of occupational exposure to formaldehyde and RBC count, but there was no relationship with WBC count. Similarly, Srivastava et al. (1992) found decreased Hb concentrations in the blood of three of six workers who were involved in producing and preparing melamine-formal-dehyde resin. However, it is not readily apparent whether the differences in RBC count and hemoglobin are related to formaldehyde exposure since this study included a limited number of subjects. In addition, this study found increased total lymphocytes (>3200 per mm^3^ of blood) in three of six workers. The authors of this study indicated that the subjects were exposed to relatively high levels of formaldehyde at different times during the day.

Recently, Zhang et al. (2010b) investigated the associations between formaldehyde exposure and various hematology parameters in subj ects working with formal-dehyde-melamine resin in two factoriesin China (median, 1.28 ppm formaldehyde) as compared with volunteer subjects from three other factories with lower formaldehyde levels (median, 0.026 ppm). Formaldehyde can dissociate from the melamine resin, become airborne, and be inhaled by the factory workers. The workers were possibly also exposed to formaldehyde dermaily, and subject to potential formaldehyde-induced skin reactions, if they touched the resin with their bare skin. The authors assessed personal workplace exposures to formaldehyde in air on 3 days for a full shift (>6 hour/shift) and to other volatile organic compounds (VOCs), including benzene, on two or three occasions within a 3-week period. The authors matched exposed and control subjects by age and sex; however, there were considerably different rates of current alcohol drinkers (yes/no answer) and recent respiratory infections (yes/no answer) in the exposed as compared with the control subjects (26% vs. 41 % and 40% vs. 29%, respectively). Alcohol intake and recent respiratory infections can influence WBC counts. As shown in Table 16, Zhang et al. (2010b) report statistically significant lower counts of total WBCs, lymphocytes, granulo-cytes, platelets, and RBCs in addition to a higher MCV but not monocytes or Hb concentration in exposed subjects relative to controls. In addition, the authors found no statistically significant difference in the growth of circulating CFU-GM hematopoietic progenitor cells ex vivo between exposed and control subjects.

In the following section, we weigh the evidence from human and animal studies discussed in Sections 5.1 and 5.2 to assess the likelihood of a hematotoxic role for formaldehyde.

### 5.3. HBWoE evaluation of formaldehyde hematotoxicity studies

Based on the available data summarized above, some have hypothesized that formaldehyde may cause hematotoxicity and leukemia in humans. We ask the following questions with regard to this hypothesis:

1. Do animal studies suggest formaldehyde exposure is causally associated with hematotoxicity and leukemia?
2. What do the human studies tell us about potential formaldehyde hematotoxicity in humans? Are the results of human studies consistent with those of animal studies?
3. What is known about the hematotoxicity of known leukemogens (e.g., benzene) from animal and human studies and how does that compare to formaldehyde?
4. Are there alternative explanations for decreased WBC and RBC counts?

As a whole, considering these questions allows for an assessment of the extent to which the hematotoxicity and animal leukemia data support either a causal association between formaldehyde exposure and leukemia or an alternative hypothesis. Importantly, one needs to consider the hematotoxicity and animal leukemia data in the context of the epidemiology and mode-of-action data, as each of the three lines of evidence inform interpretation of each other.

#### 5.3.1. Key animal studies do not provide strong evidence of an association between formaldehyde exposure and hematotoxicity and leukemia

##### 5.3.1.1. Hematology

Known leukemogens, such asbenzene, can cause bone marrow toxicity, which affects the ability of bone marrow cells to produce blood-forming cells (ATSDR, 2007). As discussed in the introduction to this section, this toxicity can be manifested in bone marrow suppression and pancytopenia, a generalized decrease of blood cellular components. This insult to bone marrow may progress to a malignancy that shows a predominance in production of one or more cell types in bone marrow that spill into peripheral blood.

We examined the available formaldehyde animal studies for signs of hematotoxicity as reflected in peripheral blood. Of 12 studies we reviewed, 9 reported no change in hematology parameters (see Table 14). These studies, which ranged from exposures lasting a few weeks to longer than 2 years, spanned a range of concentrations and durations that would be sufficient to show any changes in hematology.

Three studies reported a change in one or more hematology parameter: Dean et al. (1984) by inhalation, and Tobe et al. (1989) and Vargova et al. (1993) by oral exposure. However, these outcomes were mixed. For example, Dean et al. (1984) reported a decrease in monocytes but no other hematology parameter, whereas Vargova et al. (1993) reported an *increase* in monocytes but a decrease in lymphocyte counts. Vargova et al. (1993) also found increased Hct, Hb concentration, and RBC counts in blood, in contrast to results from Tobe et al. (1989), which indicated *decreased* Hb concentrations and RBC counts. It is noteworthy that the changes in the aforementioned studies resulted from very high exposures, particularly in oral exposure studies, ranging from 40 to 300 mg/kg body weight/day (approximately 800-5000 ppm in drinking water), as noted in Table 14.

Overall, the hematological ingestion and inhalation studies of formaldehyde we reviewed are inconsistent and eclipsed by overwhelming evidence from the same and other species of animals that show no change in hematology parameters. When found, the statistically significant changes are likely not related to formaldehyde exposure, particularly because they arise among many other statistically insignificant associations. Further, if bone marrow toxicity had occurred, it is likely that declines in more than one blood cell type would have been observed, such as is established for benzene (discussed in Section 5.3.3), and that was not reported by the authors of any of the studies we reviewed.

##### 5.3.1.2. Leukemia

We also analyzed the outcomes of animal studies that examined the carcinogenicity of formaldehyde by inhalation or ingestion. The majority of these studies, listed in Table 15 and discussed in Section 5.1, found no excess hematopoietic malignancies associated with formaldehyde exposure (Albert et al., 1982; Kerns et al., 1983; Sellakumar et al., 1985; Kamata et al., 1997; Feron et al., 1988; Til et al., 1989; Tobe et al., 1989; Takahashi et al., 1986). However, the studies by Soffritti et al. (1989, 2002), the unpublished data from the DeVoney et al. (2010 poster abstract), and Woutersen's (2007 presentation) analyses of data from Battelle Columbus Laboratories (1981) found increased incidence of hematopoietic malignances from formaldehyde ingestion and inhalation, respectively.

Soffritti et al. (1989) performed two carcinogenicity experiments by ingestion and reported statistically significantly increased hematopoietic malignancies in two reports (Soffritti et al., 1989, 2002) described in Section 5.1 and Table 15, the results of which are inconsistent and have been criticized by both ATSDR (1999) and the European Food Safety Authority (EFSA, 2006) as unreliable. The lack of confidence in the results by Soffritti et al. (1989, 2002) stems, in part, from concerns about the rodent colony where the experiments occurred. Feron et al. (1990) suggested that the elevated leukemia incidence might have been “unrelated to formaldehyde ingestion,” because of the wide range of incidence rates of hematopoietic malignancies in control animals from the same colony—as high as 19%. Moreover, a possible infection of the rat colony by *Mycoplasma pulmonis* (an organism that preferentially colonizes the respiratory tract in rats and secretes substances that can promote mitogenesis in lymphocytes) also has been presented as a potential confounder for hematolymphopoietic malignancies reported by Soffritti et al. (1989, 2002).

Other studies examining formaldehyde carcinogenicity via the oral route did not indicate an increased incidence of hematopoietic malignancies relative to control exposures. The longer-term carcinogenicity studies by both Til et al. (1989) and Tobe et al. (1989) showed no increase in these malignancies after 2 years of exposure and follow-up, consistent with Takahashi et al. (1986) who only followed animals for 40 weeks.

As reported in conference posters, DeVoney et al. (2006 poster, 2010 poster abstract) reevaluated data from Battelle Columbus Laboratories (1981) and reported increased lymphoma incidence rates for female B6C3F1 mice exposed to ∼15 ppm by inhalation (28% vs. 18% in exposed vs. control mice, respectively). According to Haseman et al. (1998), the background rate for lymphoma in these female mice, based on 1092 control mice used in National Toxicology Program (NTP) studies, is 19.9%, with a range of 6% to 44% in all studies examined. Therefore, the rates reported by DeVoney et al. (2006 poster, 2010 poster abstract) fall within background tumor rates for the rodent species use in Battelle Columbus Laboratories (1981) and do not provide sufficient evidence for formaldehyde leukemogenicity. These non-peer-reviewed results by DeVoney et al. (2006 poster, 2010 poster abstract) are contrasted by another evaluation of Battelle Columbus Laboratories (1981) data by Woutersen (2007) (as presented at the Formaldehyde International Science Conference), who found a statistically significant increased trend in lymphoma for only female mice among the rodent species and sexes examined. Moreover, there was no statistically significant association with formaldehyde when post-exposure follow-up data (3-month period) was considered. When considering the three distinct analyses of the Battelle Columbus Laboratories (1981) carcinogenicity data, in addition to the high background tumor rates in the rodent species examined, it becomes less likely that the reported inconsistent increased lymphoma incidence is related to formaldehyde exposure.

The rates presented by DeVoney et al. (2006 poster, 2010 poster abstract) for hematopoietic malignancies in female Fischer 344 rats demonstrate no dose-response for leukemia; the authors found 25%, 23%, and 24% leukemia incidence in rats exposed to 2, 6, and 15 ppm formaldehyde vs. 15% in control rats. Similarly, the leukemia rates in both exposed and control rats were either below or within the range of leukemia incidence in the controls used in NTP studies, which Haseman et al. (1998) lists as 37% (range, 24% to 54%) for leukemia in female Fischer 344 rats. Noteworthy is that the leukemias found in Fischer 344 rats likely included MCLs, which are usually observed in ∼50% and ∼28% of unexposed male and female Fischer 344 rats, respectively (Haseman et al., 1998). This high background incidence of MCLs in rats brings into question the validity of the unpublished results by DeVoney et al. (2006 poster; 2010 poster abstract). Further, as discussed in Section 5.1.2, above, MCLs are not observed in humans, and findings involving MCL incidence in animals may not be relevant in humans.

The results by Soffritti et al. (1989, 2002) and the unpublished reanalysis by DeVoney et al. (2006 poster; 2010 poster abstract) of the Battelle Columbus Laboratories (1981) data do not provide appreciable support for formaldehyde-induced leukemia in rodents. These results are unlikely to indicate formaldehyde leukemogenicity, particularly when weighed against the relatively high background rates of hematopoietic malignancies in the mouse and rat species used in these studies and the overwhelmingly negative results from nine other carcinogenicity studies in rats and mice. Finally, it is not surprising that most studies show no change in leukemia incidence with formaldehyde exposure since most studies, short- and long-term, showed no change in hematology parameters, which are important precursors in the chain of events for chemically induced leukemia.

#### 5.3.2. Key human studies do not provide strong evidence of an association between formaldehyde exposure and hematotoxicity

The studies that associate hematology parameters in humans with formaldehyde exposure are generally cross-sectional in nature. Cross-sectional environmental toxicology studies frequently involve concurrent observation of a biological endpoint and exposure to an environmental agent at a single point in time or over a short time duration. Except for a few studies, no information was available on either the methods of exposure assessment of formaldehyde or the assessment of potential confounding effects from known hematotoxicants such as benzene. For some studies, formaldehyde exposure information was absent altogether (see Table 16).

We investigated whether a possible exposure-response pattern existed between the reported airborne formaldehyde concentrations and the reported hematology responses in the studies that we reviewed. The results of studies based in China and cited by Tang et al. (2009) do not show an exposure-response relationship between formaldehyde concentrations and hematology parameters. For example, the study by Feng et al. (1996, as cited by Tang et al., 2009) reportedly showed no association between very high formaldehyde exposures (range, 0.57-15.61 ppm) and either changes in WBC counts or Hb concentrations in peripheral blood. Xu et al. (2007, as cited by Tang et al., 2009) also found no significant differences in Hb concentrations or WBC and platelet counts in association with relatively elevated formaldehyde exposures ranging from 0.36 ppm to 5.56 ppm. In contrast, Qian et al. (1998, as cited by Tang et al., 2009) estimated formaldehyde exposure to be 2.44 ppm, and found a statistically significant association between this concentration and a lower WBC count. In addition, some studies with *lower* formaldehyde exposures were statistically significantly associated with *decreased* WBC counts and other hematology parameters. For example, Cheng et al. (2004, as cited by Tang et al., 2009) found decreased WBC counts in individuals exposed to formaldehyde concentrations ranging from 0.2 ppm to 0.76 ppm, whereas Yang et al. (2007, as cited by Tang et al., 2009) found similar associations at lower concentrations not exceeding 0.036 ppm. When examining the hematology outcomes of the studies we reviewed (shown in Table 16), we found no consistent exposure-dependent pattern in either qualitative or quantitative changes.

Moreover, using the data presented by Tang et al. (2009), we determined the percent change or difference in total WBC counts between exposure groups when these counts were available; as shown in Table 16, WBC counts were between 11% and 19% lower in exposed vs. control subjects. However, when examining the studies altogether, we did not find dose dependency in the WBC, platelet, or Hb associations with formaldehyde exposure measurements.

Some of the Chinese occupational studies that reported significantly lower WBC concentrations in exposed subjects also reported formaldehyde concentrations in air that were lower than those expected outside of work. For example, the air concentration ranges reported by Zhang et al. (2010b) (median, 0.026 ppm), by Yang et al. (2007, as cited by Tang et al., 2009) (0.018-0.036 ppm), and by Kuo et al. (1997) (ND-0.054ppm) overlap with concentrations reported in indoor public places in several Chinese cities (0.12 ppm [range, 0.02-0.31 ppm] as reported by Tang et al. [2009] from the Chinese Ministry of Health). These data suggest that results based on workplace exposure to formaldehyde may be confounded by non-occupational exposures, which can be as high or even higher than the occupational exposures. Appreciable non-occupational sources of formaldehyde exposure exist, particularly in China, where most of the human formaldehyde hematology studies have been conducted. Formaldehyde concentrations in indoor air of homes have been measured up to 0.5 ppm in China (mean, 0.19 ppm) and certain dietary items have been found to contain several hundreds or even thousands of milligrams of formaldehyde per kilogram (as reviewed by Tang et al., 2009). The authors' lack of accounting for non-occupational sources of formaldehyde exposure adds to the uncertainties in the study outcomes and diminishes their credibility for a hematotoxic role for formaldehyde.

There are many uncertainties about the Chinese hematology studies as reported by Tang et al. (2009). For example, Tang et al. (2009) suggest that Kuo et al. (1997) shows a statistically significant inverse relationship between formaldehyde concentrations and WBC counts. Although Kuo et al. (1997), the only study among those cited by Tang et al. (2009) available in English, indeed shows such an association (-.33, *p*< .05), it does not show a significant relationship between exposure measurements and 11 other hematology parameters, including RBC and individual WBC-type counts. Further, the authors collected peripheral blood on two occasions 1 year apart and found associations only with the second but not the first blood sample. Subjects in the Kuo et al. (1997) study were employed for an average of 3 years at the study locations; if the association between formaldehyde exposure and this lone hematology parameter is real, then it should have been consistent in both blood samples collected. When considering the uncertainties associated with this readily obtainable study (Kuo et al., 1997), many questions arise about the full outcomes and exposure-related uncertainties in the findings of the other studies cited by Tang et al. (2009) (which are unavailable in English).

The most recent study to assess hematotoxicity in humans exposed to formaldehyde is Zhang et al. (2010b). The study provides some associations between formaldehyde exposure and changes in hematology, but also demonstrates serious weaknesses in the study design. The hematology findings by Zhang et al. (2010b) do not consistently support a hematotoxic role for formaldehyde. For example, the authors found statistically significant lower WBC, RBC, and platelet counts in exposed vs. control factory workers, yet they found no statistically significant difference between exposed and control subjects in relation to colony formation of myeloid progenitor cells (CFU-GM), which give rise to granulocytes and macrophages, cultured from blood. If it were possible for formaldehyde to cause direct or indirect toxicity to bone marrow, a decreased ability of CFU-GM to grow in culture would likely be observed, but this did not occur.

It is not clear from the Zhang et al. (2010b) data whether all subjects with decreased WBC counts also had decreased RBC counts and vice versa. If bone marrow toxicity was indeed in progress in the subjects exposed to higher levels of formaldehyde, then both WBC and RBC counts would be lower in the same individuals. In addition, because the WBC and RBC counts were pooled, it is impossible to determine if outliers in either group might have influenced the results, since subject-specific hematology parameters are not reported. Therefore, one cannot make definitive conclusions concerning these data.

We investigated the consistency between the animal and human study outcomes. As we discuss, animal studies generally show no evidence of formaldehyde-induced hematotoxicity. Human studies, on the other hand, show inconsistent associations between formaldehyde exposure measurements and hematologic parameters. Even when hematology changes, such as depressed WBC counts in the blood, are associated with formaldehyde, these associations are not exposure related, and do not agree with the findings from animal studies. This lack of concordance between human and animal studies does not provide evidence to support an argument for formaldehyde-induced hematotoxicity, unlike benzene and other leukemogens that show concordance between animal and human data (as discussed in Section 5.3.3). However, the limited number of human studies available, and the inconsistencies among them, warrants the need for well-controlled human studies with respect to exposure assessment and subject-matching between the exposed and control groups.

The available human hematotoxicity studies are cross-sectional in nature. In cross-sectional studies, both exposure and outcome are evaluated at the same time. A major weakness of cross-sectional environmental toxicology studies is that a chemical measurement at one point of time may not be indicative of earlier exposures that may have caused the biological outcome. Also, inappropriate subject-group matching (e.g., for smoking, drinking, age, sex) may result in findings of differences in biological outcomes associated with the agent in question when there are in fact none. In particular, Zhang *et al.* (2010b) only matched subjects by age and sex; however, there was a considerably higher rate of recent respiratory infections (yes/no answer) in the exposed vs. control subjects (40% vs. 29%, respectively). Zhang et al. (2010b) report that subjects were screened by physicians and trained questionnaire administrators. However, no listing of medications or medical conditions is available for the subjects in this study. We discuss possible confound-ers in the subsequent sections, particularly from dermal exposure to formaldehyde and respiratory infections that can possibly modulate the associations in the studies we reviewed for human formaldehyde hematotoxicity.

#### 5.3.3. If formaldehyde causes leukemia in humans, it is likely due to a mechanism that is different from that observed with known leukemogens

Hematotoxicity has been demonstrated in both animals and humans exposed to leukemogens (i.e., benzene, chemotherapeutic alkylating agents, and x-ray and gamma radiation). This hematotoxicity can be illustrated with benzene. Benzene has been frequently found to cause pancytopenia in animals (e.g., Aksoy et al., 1972; Farris et al., 1997, both as cited in ATSDR, 2007) and humans (e.g., Kipen et al., 1989; Schnatter et al., 2010; ATSDR, 2007). Moreover, numerous studies have shown that exposure to benzene can cause leukemia and other hematopoietic malignancies in animals by inhalation and oral routes in several rodent species of both sexes (Snyder et al., 1984; Cronkite et al., 1984, 1985, 1989; all as cited in ATSDR, 2007). Many epidemiology studies have also shown robust associations between exposure to benzene and increased risk of leukemia (e.g., Rinsky et al., 1987, 2002; Yin et al., 1996; Infante et al., 1977; ATSDR, 2007). The mechanism for benzene hematotox-icity and leukemogenicity is dependent on its metabolism to reactive intermediates and is well established as having the ability to affect bone marrow cells directly (ATSDR, 2007).

Other leukemogens have cytotoxic and genotoxic properties similar to benzene. These agents can affect all cells, particularly rapidly dividing cells such as bone marrow. For example, cyclophosphamide, a chemother-apeutic alkylating agent, has produced hematopoietic malignancies in exposed animals (Schmahl and Habs, 1979) as well as leukopenia in humans (Bower et al., 2004; Tjan-Heijnen et al., 2001) and animals (Wang et al., 2002; Nohynek et al., 1997). X-ray and gamma-radiation also have been repeatedly shown to cause leukemia and bone marrow toxicity in animals and humans (IARC, 2000).

As discussed in the preceding section, the available human studies lack the appropriate exposure and subject information and the consistent outcomes to make a convincing case for formaldehyde leukemogenicity. Upon reviewing the available studies of formaldehyde hematology effects, we found no consistent evidence of hematotoxicity in humans. Moreover, the animal studies using mice, rats, dogs, and monkeys, often of both sexes, overwhelmingly reported no evidence of changes in hematology parameters, as shown in Table 14. Therefore, if formaldehyde causes leukemia in humans, it must be by a mechanism that is different from that observed with known leukemogens and is likely specific to humans and not common to rodents. Table 17 illustrates the divergence of formaldehyde from known leukemogens in terms of hematotoxicity indicators.

**Table 17:** Comparison of formaldehyde hematotoxicity to known leukemogens.

	Benzene	Cyclophosphamide	Radiation	Formaldehyde
Pancytopenia in animals	Yes	Yes	Yes	No
Hematopoietic malignancies in animals	Yes	Yes	Yes	No
Pancytopenia in humans	Yes	Yes	Yes	More research needed

#### 5.3.4. There are alternative explanations for the pancytopenia reported by Zhang et al. (2010b) and the leukopenia reported by other studies

As reviewed above, animal studies generally do not show a hematotoxic effect of formaldehyde. However, several human studies report that formaldehyde is associated with lowered WBC and RBC counts in peripheral blood (see Table 16). Our review of available human studies of formaldehyde hematotoxicity finds that these studies do not sufficiently explain some of the formaldehyde-associated depression in RBC and WBC counts in “exposed” vs. “control” subjects. When we consider the uncertainties in these associations and the absence of a clear dose-response in the available studies, we find that there are many potential confounders to a possible formaldehyde-associated decline in WBC and RBC counts in blood. Some of these confounders include inappropriate matching by exposure due to consideration of only airborne measurements of formaldehyde (and not dermal or oral), no reported assessment of non-occupational exposures to formaldehyde, differences among groups in alcohol intake and respiratory infections, and the possible effect of formaldehyde on hematology parameters in peripheral blood (via dermal irritation and sensitization, as discussed in Section 5.3.4.1) in addition to other issues such as nutrient imbalance and certain medications, all of which may have a significant effect on hematology parameters. Further, unlike established leukemogens such as benzene (as discussed in Section 5.3.3), formaldehyde has not been associated with bone marrow toxicity or aplastic anemia in occupationally exposed subjects.

##### 5.3.4.1. Subjects exposed to formaldehyde share common immunology markers with subjects having dermatitis or other inflammatory conditions

As reviewed by Deane and Hickey (2009), epidermal inflammation in atopic dermatitis, psoriasis, and allergic contact dermatitis involves the movement of leukocytes from peripheral blood to skin. Singbartl and Ley (2004) also describe the process of leukocyte recruitment to inflamed tissues in the case of acute renal failure as occurring in a cascade-like fashion that encompasses capture, rolling, activation, firm adhesion, and tissue translocation of leukocytes. These mechanisms may contribute to a decrease in blood cells from peripheral blood in subjects with certain inflammatory conditions in the skin or other organs.

The ability of liquid formaldehyde to cause dermatitis, skin irritation, and immune modulation in occupational and non-occupational settings is well documented. For example, Nethercott and Holness (1988, as cited in ATSDR 1999) showed an 11% prevalence of contact skin dermatitis (3% positive formaldehyde skin-patch tests) in embalmers working at funeral homes vs. 0% in controls. Similar results have been reported for nurses exposed to formaldehyde disinfectant (Rudzki et al., 1989, as cited in ATSDR 1999). Further, in a review of formaldehyde in cosmetic products, de Groot and Maibach (2010) find that formaldehyde applied to skin has been shown to induce dermatitis from short-term use. Finally, in a review of skin sensitivity to formaldehyde in various populations, de Groot et al. (2009) found a 4.1% prevalence in one study of Chinese subjects and up to 9.2% in studies from the United States. If occupational dermal exposure to formaldehyde results in skin irritation and dermatitis, which influence changes in concentrations in peripheral blood of leukocytes and other hematology parameters, these changes may explain the heterogeneity in response with different exposure concentrations in the human studies summarized in Table 16. It is noteworthy that bone marrow is dynamic in that cell loss is compensated by cell production, and this characteristic should be the subject of further study in the case of dermal reactions.

There are similarities between the observed effects in peripheral blood of some formaldehyde-exposed subjects (via inhalation and possibly dermally, as discussed above) and in subjects with dermatitis conditions. For example, Yoshino et al. (2000) found evidence that the degree of clinical dermatitis was associated (positively or negatively) with peripheral mononuclear WBC counts and that the proliferation of peripheral mononuclear cells maybe suppressed in severe atopic dermatitis cases; the authors suggest that this is related to the high rate of T-cell apoptosis in severe atopic dermatitis. Similarly, Forte et al. (2009) found that a reduction in chemotactic response and phagocytic activity by neutrophilic and/or mononuclear phagocytes in the majority of patients with atopic dermatitis ranged from moderate to severe. Further, Lebre et al. (2008) found that myeloid dendritic cells and plasmacytoid dendritic cells from patients with atopic dermatitis showed defective interleukin (IL)-12, tumor necrosis factor (TNF)-a, and interferon (IFN)-a production; the authors suggest that these immune indicators may contribute to the maintenance of an allergic state in these patients. Dermatitis conditions have been associated with increased eosinophil counts (290 vs. 153.3 cells/mm^3^, p<.05) in the blood of patients with atopic dermatitis vs. healthy subjects, respectively (Jenerowicz et al., 2007). From the peripheral blood eosinophil count and eosinophil percent of blood cells, we calculated a lower mean WBC count in subjects having dermatitis vs. healthy subjects (4581 vs. 4746 WBCs/mm^3^, respectively; 30 subjects per group), but it was not possible to assess whether these counts were statistically significantly different from each other, since the raw data were not provided for the individual subjects in this study. Since hematology changes can be associated with allergic reactions in general, and dermatitis in particular, and because formaldehyde can cause dermatitis and dermal sensitivity, an assessment of skin reactions to formaldehyde is necessary when investigating formaldehyde-induced hematology effects.

If skin reactions are indeed present in the study subjects, they may confound the hematology findings reported by many of the aforementioned studies. Dermal exposure to formaldehyde and its effects on clinical and subclinical skin sensitivity reactions are not reported in the human studies we reviewed. However, it is possible that the subjects in the studies by Zhang et al. (2010b), Lyapina et al. (2004), Srivastava et al. (1992), and in the studies cited by Tang et al. (2009) were exposed dermally to formaldehyde. In addition, the status of sensitization or inflammation in exposed vs. control individuals in these studies is largely unknown. Further, Farage (2008) reported that skin reactions may not be easily diagnosed by visual inspection and may require more sophisticated technology that is not widely available. Therefore, even if subjects with higher air exposures to formaldehyde had some form of skin reaction to formaldehyde, the possibility exists that this condition would not be detected by a clinician.

##### 5.3.4.2. A recent respiratory infection can result in hematological changes—Subjects with exposure to form aldehyde in the study by Zhang et al. (2010b) were more likely than control subjects to have had recent respiratory tract infections

Several studies suggest that respiratory infections can be associated with leukopenia, or decreased WBC counts in peripheral blood, in humans. Cummins et al. (1998) found a 5% decrease (*p*=.02) in total leukocyte counts in blood and a 9% decline (*p* = .001) in lymphocyte counts in 70 elderly subjects 4 weeks after they received an influenza vaccine. These results are supported by those from three cases of pediatric influenza infections that were associated with declines in peripheral blood WBC counts (Rice and Resar, 1998). Further, a study by Shen et al. (2008) showed that not only do infections modify hematology parameters, but also that the type of infection could be important. For example, children with influenza B infection had a significantly lower total WBC count than those with influenza A infection. Influenza infection has also been shown to cause or exacerbate bone marrow suppression in mice (Lavrov and Semenkov, 1991; Hyland et al., 2005). Shen et al. (2008) found that leukopenia is not an uncommon occurrence in influenza infections and that this decline in WBC counts is possibly related to B-lymphocyte apop tosis in bone marrow.

The higher rate of recent respiratory infections in the exposed vs. control groups of the study by Zhang et al. (2010b) could have resulted in confounding of hematology parameters. Alternatively, it may be argued that the higher rate of recent respiratory infections in the exposed workers is due to lower WBC counts or thatrespiratory tract infections could either increase or decrease WBC counts in peripheral blood (Mayo Clinic, 2008) and may, therefore, be unrelated to the findings in this study. However, all reported WBC counts for formaldehyde-exposed subjects and their controls are above 4900 cells/mm^3^ of blood (e.g., Tang et al., 2009; Zhang et al., 2010b), which are higher than the Mayo Clinic's benchmark of 3500 cells/mm^3^ for leukopenia (Mayo Clinic, 2008). Therefore, it is less likely that the lower WBC count is the cause of the recent infections in the exposed subjects. Better-matched exposure and control groups in future studies may eliminate this potential confounder.

##### 5.3.4.3. Other unmeasured potential confounders

As discussed in the preceding subsections, several conditions may be associated with decreased WBC and RBC counts in peripheral blood. In a study of adult Japanese male office workers, Nakanishi et al. (2003) found that WBC counts increased with increasing body mass index and smoking, but decreased with alcohol intake, nutritional balance, and hours worked per day. Here we focus on two possible confounders of hematology parameters that may be associated with oral or inhalation exposure to formaldehyde: (1) the effects of formaldehyde on the hypothalamic/pituitary/adrenal (HPA) axis involvement in WBC count modulation; (2) the effect, on the kidneys, of possible exposure to melamine from formaldehyde-melamine resins (which were the source of formaldehyde exposures in the study by Zhang et al., 2010b); kidneys are important in producing erythropoietin, the hormone responsible for inducing RBC production.

There is evidence to suggest that formaldehyde exposure can modulate WBC counts in peripheral blood via an endocrine pathway. Brondeau et al. (1990) found that exposure to airborne irritants (including formaldehyde) caused leukopenia at irritant levels (≥43 ppm for formaldehyde, in an exposure-dependent manner) over a 4-hour exposure, and that this effect was prevented by removal of the adrenal gland, suggesting a possible role for this gland in apparent hematological effects. Sari et al. (2004) also found that relatively low concentrations (0.08, 0.4, and 2.0 ppm) of formaldehyde increased hypothalamus/pituitary/adrenal (HPA) axis activity by increasing the numbers of both hypothala-mus corticotropin releasing hormone-immunoreactive neurons and pituitary adrenocorticotropin hormone (ACTH)-immunoreactive cells in mice. These neuronal changes were paralleled by increased mRNA expression of pituitary ACTH, which functions in regulating adrenal gland function. It is also well established that the adrenal glands produce glucocorticoids, mainly cor-tisol, a steroid that can suppress the immune response (Cotran et al., 1999). Although more research is needed to investigate the potential effect of formaldehyde on WBC counts via an endocrine pathway, particularly at occupationally relevant concentrations, there is suggestive evidence that it occurs. This HPA pathway should be considered when evaluating hematology data associated with formaldehyde exposure.

The recent discovery that melamine can cause or contribute to renal toxicity may have implications for the consumption of melamine when it is either present as an adulterant in food or inhaled during its manufacture or processing. In patients who have kidney disease it is likely that production of erythropoietin, the hormone responsible for inducing RBC production, is depressed. As a result, the bone marrow makes fewer RBCs and therefore patients with kidney disease often have to take erythropoietin supplements (NIH, 2008). This notion of melamine-induced changes in RBC counts and hemoglobin concentration finds support from Srivastava et al. (1992) who reported declines in hemoglobin concentration and *elevations* in lymphocyte counts, but not other hematology indicators, in workers with considerable exposure to formaldehyde-melamine resin. Moreover, Dobson et al. (2008) found that melamine or melamine cyanuric acid ingestion in rats caused renal toxicity. Further, acute renal failure has also been reported in human infants in Beijing, China, who were exposed to melamine via a popular Chinese brand of milk formula, “Sanlu” (Sun et al., 2010). The role for melamine in renal toxicity and how it might be related to changes in RBC counts and Hb concentration in blood is important, particularly when analyzing the results of the study by Zhang et al. (2010b). Consideration of melamine exposure in the Zhang et al. (2010b) study participants is important, since the study participants were potentially exposed by inhalation and ingestion to formaldehyde-melamine resins at work, and they were possibly exposed to melamine from food items made or contaminated with melamine-adulterated milk powder outside work.

Finally, there are several other potential confounders of hematology parameters in occupational studies. For example, alcohol consumption, which is a potential con-founder in the study by Zhang et al. (2010b), can modulate immune function (Szabo and Mandrekar, 2009). Also, nutritional deficiencies in folic acid and cyanocobalamin (vitamin B12) have been associated with megaloblastic anemia, which manifests with faulty RBCs that are larger than normal (increased MCV) (Morris et al., 2007). Further, certain herbal supplements, such as Echinacea, have been associated with depressed WBC counts after chronic ingestion (Kemp and Franco, 2002). It is thus possible that WBC and RBC counts in peripheral blood are modulated by formaldehyde exposure in mechanisms involving extramedullary systems (i.e., outside the bone marrow); this would contribute to confounding in epidemiology studies that result in observed differences between exposed and controls.

Future studies investigating a possible association between exposure to formaldehyde and hematotoxicity should consider a number of confounders, including, but not limited to, the ones discussed here.

### 5.4. Summary

As a whole, the available studies of formaldehyde hematotoxicity in both animals and humans provide little evidence to support the account that formaldehyde exposure is causally associated with leukemia. The animal studies generally reported neither hematotoxicity nor leukemia associated with formaldehyde inhalation or ingestion. The two studies, one of which is not peer-reviewed, that reported some evidence of formaldehyde-induced leukemia are not convincing of such an association due to (1) inconsistent and potentially flawed data that has been dismissed by both EFSA and ATSDR (as discussed in Section 5.3.1.2) (Soffritti et al., 1989, 2002); (2) the high background tumor rate in the animal models used (DeVoney et al., 2006 poster, 2010 poster abstract); (3) the lack of corroboration from numerous other studies that examined the same endpoints in animals.

A few human studies, as cited by secondary sources, may be consistent with hematotoxicity, but they are inconsistent with other study findings and plagued by possible confounding. As discussed in Section 5.3.2, the studies suggestive of hematotoxicity are reported by Tang et al. (2009) and Zhang et al. (2010b). However, because the only study of hematological effects cited by Tang et al. (2009) available in English (Kuo et al., 1997) is associated with several uncertainties, the conclusions drawn by Tang et al. (2009) are weakened. Until the other studies cited by Tang et al. (2009) are translated, many questions exist about the outcomes and exposure-related uncertainties in the findings. Many medical and lifestyle factors can contribute to changes in hematology, particularly declines in WBC and RBC counts. The study by Zhang et al. (2010b) provides some evidence to support an association between formaldehyde and hematotoxicity; however, as discussed in Sections 5.3.2 and 5.3.4, this study's outcomes are mixed and may suffer from potential confounding of results by recent respiratory tract infections and leukopenia resulting from possible dermatitis. When considering the many possible known and unknown confounders in the studies we reviewed, such as dermatitis, respiratory infection, alcohol consumption, non-occupational sources of formaldehyde, etc., it is impossible to rule out confounding. In addition, many of the human studies are cross-sectional and therefore cannot adequately show cause and effect. Moreover, the available data from human studies do not provide sufficient proof for formaldehyde-induced hematotoxicity particularly when animal studies provide strong evidence *against* it. If formaldehyde is hematotoxic in humans, this toxicity would likely be via a mechanism not feasible in rodents, rhesus monkeys, or beagle dogs, since formaldehyde exposure does not cause hematotoxicity in these animals, therefore bringing into the question of biological plausibility of formaldehyde-induced hematotoxicity in humans.

Finally, the question of potential formaldehyde-induced hematoxicity can be explored by considering information on epidemiology and mode-of-action studies. As part of the HBWoE approach, one considers the cross-discipline integration of hypothesized effects. As discussed herein, the epidemiology and mode-of-action data cast doubt on the ability of inhaled formaldehyde to interact with and perturb hematopoiesis, which complicates further the plausibility of a conclusion of causal association based on the observations in the hematotoxicity and animal leukemia studies.

## 6. Weight of evidence regarding a plausible mode of action for formaldehyde leukemogenesis

In the following analysis, we examine the data relevant to the modes of action that have been proposed for formaldehyde leukemogenesis. We focused on studies that examined formaldehyde metabolism and distribution, and genotoxicity in animals, humans, and in vitro. We conducted literature searches, using PubMed and several search terms in combination with “formaldehyde”: “genom*,” “chromosom*,” “micronuclei,” “cytogenetic,” “DNA damage,” “genotox*,” “mutagen*,” “metabol*,” “toxicokinetic,” and “pharmacokinetic.” We also relied on recent key review articles and agency reports (IARC, 2006; US EPA, 2010; Heck and Casanova, 2004; Pyatt et al., 2008; Golden et al., 2006), as well as references within those reports and papers found in the PubMed search.

As discussed, the epidemiology data do not support a causal association between formaldehyde exposure and leukemia. In addition, the available studies of formaldehyde hematotoxicity in both animals and humans provide little evidence for formaldehyde-associated leukemia. The animal studies generally reported neither hematotoxicity nor leukemia associated with formaldehyde exposure, and although a few human study findings are consistent with hematotoxicity, they are inconsistent with other study findings and plagued by possible confounding.

Despite these findings, three modes of action for formaldehyde leukemogenesis have been hypothesized by Zhang (2009, 2010a) and are also discussed in US EPA's recent draft toxicological profile for formaldehyde (US EPA, 2010). The proposed modes of action are as follows:

1. Formaldehyde targetingbone marrowhematopoietic stem cells—formaldehyde complexes as a hydrate [CH_2_(OH)_2_] that could potentially reach the bone marrow, where it could directly induce DNA damage and chromosomal aberrations in hematopoietic stem or progenitor cells, leading to leukemia.
2. Formaldehyde targeting nasal stem cells (nasal-associated lymphoid tissue, or NALT)—nasal stem cells are damaged by formaldehyde, released from the nasal passage, circulate in the blood, and are eventually incorporated into bone marrow leading to leukemia.
3. Formaldehyde targeting circulating hematopoietic stem cells—stem cells circulate from marrow to nasal tissue where they are transformed by formaldehyde (pre-mutagenic lesions), and then migrate back to bone marrow, eventually leading to leukemia.

Here we first describe what is known about formaldehyde metabolism, biological distribution, and genotoxicity. We then provide a weight-of-evidence analysis of the formaldehyde data with regard to the three proposed modes of action.

### 6.1. Formaldehyde toxicokinetics

The toxicokinetics of formaldehyde has been extensively studied and is summarized in recent reviews and agency toxicological profiles (ATSDR, 1999; ATSDR, 2010; Heck and Casanova, 2004; IARC, 2006; US EPA, 2010). Formaldehyde is a normal by-product of several metabolic pathways in mammals, and is naturally present in tissues, cells, and biological fluids. Under physiological conditions, it exists in equilibrium, predominantly in its hydrated form methanediol [CH_2_(OH)_2_], with less than 0.1% as free formaldehyde. Formaldehyde is water soluble and highly reactive; therefore, it is readily absorbed and metabolized in biological systems. It is primarily metabolized by glutathione-dependent formaldehyde dehydrogenase (FALDH) and aldehyde dehydrogenases (ALDHs). Formaldehyde enters the “one-carbon” pool and is readily incorporated into macromolecules in the body. In rats exposed to [^14^C]formaldehyde via inhalation (0.63 or 13 ppm), the exhaled fraction was independent of exposure concentration, with 40% of the ^14^C incorporated into macromolecules and 40% exhaled as ^14^CO_2_, and the remainder was excreted in the feces and urine, and incorporation into macromolecules in the blood was via the one-carbon pool and not through DNA or protein adducts (Heck et al., 1983, as cited in Heck and Casanova, 2004). Salthammer et al. (2010) discusses a median concentration of 4.3 ppb formaldehyde in human breath that is likely due to endogenous sources.

The concentration of endogenous formaldehyde in human blood is approximately 0.1 mM and, as discussed in Heck and Casanova (2004), this concentration is not increased in humans who inhale 2 ppm formaldehyde for 40 minutes or in monkeys inhaling 6 ppm for 4 weeks. The inability of exogenous formaldehyde to increase blood concentrations of formaldehyde was confirmed in an analysis by Franks (2005) using a sophisticated mathematical model. These data strongly suggest that, at concentrations to which humans might be exposed, formaldehyde does not move beyond the nasal mucosa to cause effects at distant sites. Recent dosimetry, cyto-toxicity, and genomics studies conducted by Andersen et al. (2010) suggest that exposure to formaldehyde concentrations of 1 to 2 ppm would not affect formaldehyde homeostasis or increase genotoxic and cytotoxic effects in the nose or in any other tissue. Andersen et al. (2010) developed a pharmacokinetic model to estimate various forms of formaldehyde and glutathione (GSH) tissue concentrations, accounting for enogenous levels of formaldehyde, and applied the model to compare tissue concentrations with histopathology and gene expression changes in the nasal epithelium of rats. The study found that at high exposure concentrations (6 to 15 ppm), gene expression changes reflected pathways involved in cell cycle control, DNA repair, and apoptosis, with tissue responses including cell proliferation, erosion, necrosis, and increased severity of squamous metaplasia—cellular responses potentially associated with carcinogenesis. At lower exposure concentrations (less than 1 to 2 ppm), the gene expression changes likely represented extracellular responses (such as responses to irritancy and to export GSH to extracellular spaces), with tissue responses at 2 ppm reflecting mild squamous metaplasia.

### 6.2. Formaldehyde genotoxicity

Formaldehyde induces a variety of genotoxic and muta-genic effects, including DNA protein cross-links (DPX), DNA adducts, point mutations, DNA strand breaks, chromosomal aberrations (CA), deletions, sister-chromatid exchange (SCE), and micronucleus (MN) formation (ATSDR, 1999; ATSDR, 2010; Heck and Casanova, 2004; IARC, 2006; US EPA, 2010).

#### 6.2.1. DNA adducts and protein cross-links

At high exposure concentrations, formaldehyde causes DNA-protein cross-links (DPX) in the nasalmucosa of rats, upper respiratory tract of monkeys, and in vitro in human cells (Heck and Casanova, 2004; ATSDR, 1999; ATSDR, 2010; IARC, 2006; US EPA, 2010). Pharmacokinetic models have been used to study the disposition of inhaled [^14^C]formaldehyde in the respiratory tract. At very low concentrations of formaldehyde, nearly 100% is eliminated through metabolism or through non-saturable pathways other than DPX (such as protein adducts), with very little (7 × 10∼^6^%) bound as DPX. At higher concentrations (6 ppm, 6 hours) in rats and Rhesus monkeys, 91% and 96% of the [^14^C]formaldehyde in the DNA was due to metabolic incorporation, and approximately 9% and 4% of the [^14^C]formaldehyde in the DNA was bound as DPX in the nasal respiratory mucosa, respectively (Heck and Casanova, 2004). Studies suggest that formaldehyde-induced DPX are rapidly removed (24 hours) from human blood cultures treated in vitro (Schmid and Speit, 2007), and from the nasal respiratory mucosa of rats exposed via inhalation to formaldehyde (6, 10 ppm) (Heck and Casanova, 2004).

There is no strong evidence to suggest that formaldehyde causes DPX in bone marrow or WBCs (discussed in more detail in the next section). A recent study by Wang et al. (2009a) found higher levels of the formaldehyde-DNA adduct *N*^6^-hydroxymethyldeoxyadenosine (A/^6^-HOMe-dA) in leukocytes of smokers vs. non-smokers. The authors suggest that *N*^6^-HOMe-dA adducts in leukocyte DNA may be potentially important as a cause of cancer from smoking. A recent study by Lu et al. (2010), in which rats were exposed via inhalation to 10 ppm deuterium-labeled formaldehyde (i.e., [^13^CD_2_]formaldehyde) to trace the disposition of exogenous vs. endogenous formaldehyde in DNA, found exogenous formaldehyde-DNA adducts in the nasal respiratory mucosa but not at distant sites (including WBCs and bone marrow). In addition, Lu et al. (2010) found that exogenous formaldehyde caused only *N*^2^-HOMe-dG adducts in the nasal mucosa and no *N*^6^-HOMe-dA adducts; however, both adducts were found in distant sites but only from endogenous formaldehyde. Another study by Lu et al. (2011) examined molecular dosimetry (0.7, 2, 5.8, 9.1, and 15.2 ppm [^13^CD_2_]formaldehyde for 6 hours) of endogenous and exogenous *N*^2^-HOMe-dG adducts in the nasal mucosa of rats. The authors found that endogenous adducts dominated at low exposure concentrations (more than 99% and 97% endogenous at 0.7 and 2 ppm, respectively). Further, the authors examined the levels of endogenous and exogenous *N*^2^-HOMe-dG adducts in bone marrow from exposure to 15.2 ppm formaldehyde and found that exogenous adducts were not detectable. A similar study conducted by the same group (Moeller et al., 2011) examined the levels of endogenous and exogenous *N*^2^-HOMe-dG adducts in the nasal mucosa and bone marrow of cynomolgus macaques exposed to 1.9 and 6.1 ppm [^13^CD_2_]formaldehyde for 6 hours a day for 2 consecutive days. The authors observed readily detectable levels of exogenous and endogenous adducts in the nasal mucosa at both exposures; however, only endogenous adducts were detectable in the bone marrow. These data strongly suggest that the results observed by Wang et al. (2009a) may be specific to effects from cigarette smoke (i.e., the generation of formaldehyde from metabolism of *N*-nitrosodimethylamine [NDMA] and 4-(methylnitorosamino)-1 -(3-pyridyl)-1 -butanone [NNK]) and not from exogenous formaldehyde. In addition, Lu et al. (2010, 2011) and Moeller et al. (2011) provide strong evidence to support the biological implau-sibility of distant site carcinogenicity, such as leukemia, from inhaled formaldehyde, while providing evidence that formaldehyde inhalation can lead to DNA adducts in respiratory nasal epithelium. In addition, Neuss et al. (2010) show that human nasal epithelial cells pre-ex-posed in vitro to high concentrations of formaldehyde do not cause DNA damage (DPX) in co-cultivated isolated human lymphocytes, lending further support that formaldehyde that has entered the nasal epithelial cells does not move beyond these cells to damage DNA in other cells in close proximity (discussed in more detail below with respect to the NALT hypothesis).

##### 6.2.2. Clastogenic and cytogenetic effects

In vivo mammalian formaldehyde genotoxicity assays have examined clastogenic and cytogenetic effects (CA, SCE, and MN formation) in rodents and humans, and the results have been summarized (ATSDR, 1999; ATSDR, 2010; Heck and Casanova, 2004; IARC, 2006; US EPA, 2010). As presented in these reviews, the cytogenetic results in humans and animals are conflicting, showing both positive and negative effects. In humans, the majority of these studies have been carried out in nasal or oral mucosa (to examine site of direct contact) and in peripheral blood lymphocytes (PBLs) (to examine distant-site toxicity). As reviewed by Speit and Schmid (2006) and agency reviews (IARC, 2006; US EPA, 2010), the published studies suggest that inhalation of formaldehyde leads to increased MN frequencies in nasal and/or buccal mucosa cells. There are a number of issues with these studies, however, including incomplete information on study design, exposure, and potential confounding factors (Speit and Schmid, 2006). Speit and Schmid (2006) suggest that because of this, it is not yet possible to make meaningful conclusions regarding local genotoxic effects of formaldehyde.

From our review of the current literature, and from studies summarized in recent agency reviews (IARC, 2006; US EPA, 2010; Jakab et al., 2010; Jiang et al., 2010; Pala et al., 2008), to date, approximately 20 studies have examined the cytogenetic effects of formaldehyde in human PBLs, as a means for examining distant-site toxicity. These data are insufficient and conflicting, with both positive and negative results. As discussed in several recent reviews (Heck and Casanova, 2004; Pyatt et al., 2008; Golden et al., 2006), and in more detail in the next section, interpretation of the positive findings in humans, particularly in the context of leukemia, is problematic given (1) potential confounding in the studies, including diet and life style differences, or the lack of good exposure information; (2) the lack of evidence to suggest that DNA damage in human PBLs is a model for DNA damage in stem cells, since these effects have not been shown to occur in stem cells that can transition to leukemia; and (3) similar results have not been found in controlled animal studies. For example, Kligerman et al. (1984) found no statistically significant increase in SCE or chromosome breakage in PBLs of rats exposed to formaldehyde (0.5, 6, or 15 ppm). A similar study carried out recently by Speit et al. (2009) found that formaldehyde (0.5, 1, 2, 6, 10, and 15 ppm) did not induce any significant genotoxic effects (DPX, SCE, or MN) in PBLs of rats.

### 6.3. HBWoE evaluation of the proposed modes of action for formaldehyde as a leukemogen

The plausibility of the three proposed modes of action has been extensively reviewed by others (Pyatt et al., 2008; Golden et al., 2006). We have considered these reviews, in addition to the primary formaldehyde inhalation toxicity literature, and have come to the following questions with regard to the proposed modes of action:

1. What is the evidence that formaldehyde exposure induces carcinogenic (or genotoxic) transformation directly in bone marrow?
2. What is the evidence that formaldehyde can induce carcinogenic (or genotoxic) transformation in nasal-associated lymphoid tissue (NALT), or peripheral hematopoietic stem cells (HSCs)?
3. Is the DNA damage observed in the formaldehyde genotoxicity studies consistent with DNA damage associated with leukemia?
4. If formaldehyde could induce systemic DNA dam age, what concentrations in the nose would it take to reach levels higher than endogenous formalde hyde DNA adduct levels in the NALT or circulating HSCs to cause a sufficient level of DNA damage that would induce cell proliferation in the bone marrow? Would these concentrations be relevant to typical human formaldehyde exposures? How do these con centrations compare to levels that would also cause irritation?
5. If formaldehyde could induce DNA adducts above endogenous levels in NALT or circulating HSCs, what is the likelihood that these cells would home back to healthy bone marrow to cause leukemia?

As a whole, considering these questions allows for an assessment of the extent to which the genotoxicity and mode-of-action data support either a causal association between formaldehyde exposure and leukemia or an alternative hypothesis. Importantly, one needs to consider the mode-of-action data in the context of the epidemiology and hematotoxicity data, as each of the three lines of evidence inform interpretation of the other.

#### 6.3.1. There is no consistent evidence that inhaled formaldehyde induces genotoxicity in bone marrow, NALT, or peripheral HSCs that might lead to leukemia

Although the evidence clearly indicates that formaldehyde induces DPX in nasal mucosa of rats and the upper respiratory tract of monkeys (Heck and Casanova, 2004), a large body of evidence suggests that formaldehyde does not move beyond the respiratory mucosa to induce systemic geno toxic effects and cellular transformation (Heck and Casanova, 2004; Pyatt et al., 2008; Golden et al., 2006, Andersen, et al., 2010). These data are discussed in more detail below in the context of the distant sites (bone marrow, NALT, or peripheral HSCs) relevant to the proposed formaldehyde leukemogenic modes of action.

##### 6.3.1.1. Bone marrow

Zhang et al. (2009) hypothesize that formaldehyde may potentially reach the b one marrow in its hydrated methanediol form where some level of free formaldehyde may exist in equilibrium with methandiol so that it could react with bone marrow stem cells to cause leukemia. This is very unlikely, however, given that, as discussed above, the levels of formaldehyde in the blood do not increase even with reasonably high exposure levels in humans (2 ppm). As discussed below, there are studies to support the implausibility of this mechanism.

As discussed in Heck and Casanova (2004), studies using radiolabeled formaldehyde have shown that there is a lack of detectable DPX in the bone marrow of rats exposed to 15 ppm formaldehyde (Casanova-Schmitz et al., 1984), in bone marrow of GSH-depleted rats exposed to 10 ppm formaldehyde (Casanova and Heck, 1987), and in Rhesus monkeys exposed to formaldehyde at concentrations as high as 6 ppm (Heck and Casanova, 2004). Further, as discussed above, recent studies (Lu et al., 2010, 2011; Moeller et al., 2011), using [^13^CD_2_]form-aldehyde, clearly indicate that exogenous formaldehyde does not induce DNA damage beyond the nasal tissue (i.e., bone marrow).

In addition, cytogenetic assays in bone marrow of Sprague-Dawley rats (Dallas et al., 1992) exposed to 15 ppm formaldehyde, and mice exposed to formaldehyde via intraperitoneal injection (Natarajan et al., 1983 as cited in US EPA, 2010; Gocke et al., 1981), observed no significant increase in CA or MN in bone marrow cells relative to controls. In contrast, one study by Kitaeva et al. (1990, abstract only) of Wistar rats exposed to very low concentrations of formaldehyde (0.4 to 1.2 ppm) observed an increased incidence of CA in bone marrow cells relative to controls. This one study is not supported by results from the other three studies discussed. In addition, as discussed in Heck and Casanova (2004) and in Golden et al. (2006), this study is hampered by a lack of critical experimental details (i.e., dose levels and other experimental procedures are not clear, and statistical methods were not described properly) that precludes its use in drawing any meaningful conclusions.

Overall, the weight of evidence does not support the proposed mode of action that inhaled formaldehyde moves beyond the nasal respiratory mucosa to cause genotoxicity in the bone marrow.

##### 6.3.1.2. Stem cells in the NALT

Zhang et al. (2009) hypothesize another potential mode of action involving direct induction of mutations in the pluripotent stem cells of the nasal passage (or the NALT), and that these stem cells could then be released into the circulation where they could eventually make their way to the bone marrow. There are several lines of evidence, discussed below, that suggest the implausibility of this proposed mechanism.

First, if precursor cells in nasal tissue were acted upon in this way, there should also be generation of chloro-mas in the nasal tissue, since isolated accumulations of myeloid tumor cells would be expected to originate from the same proposed precursor cells in nasal tissue. There is no sign of chloromas, however, among formaldehyde-exposed workers in the current literature. Further, as discussed in Pyatt et al. (2008), all lymphoid tumors arising from the NALT have been classifiable as non-Hodgkin's lymphoma (NHL), which is not elevated in the formaldehyde occupational epidemiology studies. The lack of chloromas and NHL arising in the NALT (nasal lympho-mas) in the epidemiology data provide strong evidence against this mode of action.

Recent experimental evidence directly examining this proposed mechanism suggests its implausibility. Kuper et al. (2009) examined the proliferative effect of formaldehyde on the NALT and local lymphnodes in F344 rats and B6C3F1 mice exposed to 0, 0.5, 1, 2, 6, 10, and 15 ppm formaldehyde for 28 days. The authors found an increased proliferation rate in the nasal epithelial cells and a slight to moderate simple hyperplasia of the NALT in rats exposed to 15 ppm but not at lower concentrations, and no increases were observed at any concentration in mice, suggesting that at concentrations of less than 15 ppm formaldehyde, sufficient levels of formaldehyde do not move beyond the nasal mucosa to the NALT to induce cell proliferation. Given these observations, it is worth considering whether it is biologically plausible to incur enough damage in the NALT tissue, from typical human formaldehyde exposures, that would be sufficient to have other manifestations. Although levels lower than 15 ppm formaldehyde do not induce proliferation in the NALT, one might argue that DNA damage may still occur at low levels of exposure; if this damage is in a pluripotent stem cell that is released into the circulation and the DNA is sufficiently damaged such that carcinogenic initiation could occur, this cell might home back to bone marrow to cause leukemia. But, one must ask whether this is quantitatively plausible, particularly since mucosa-associated lymphoid tissue (such as the NALT) represent small concentrations of tissue. Stochastic models of carcino-genesis have been developed that suggest human cancers are the result of a multistage process requiring at least two genetic alterations for carcinogenic transformation (Moolgavkar et al., 1999). With the understanding that malignant tumors arise from a single malignant progenitor cell, we must ask whether there is a strong enough stochastic argument to support the hypothesis that formaldehyde exposure (at typical human exposure concentrations of 2 ppm or less) would hit enough stem cells in the NALT such that there is a reasonable likelihood that the critical genes, in at least one of the stem cells that is released into the circulation, would be sufficiently damaged to cause carcinogenic initiation, and further that there is a reasonable likelihood that the initiated stem cell will home back to healthy bone marrow to cause leukemogenesis. Given the stochastic nature of carcinogenesis, the relatively small amount of NALT tissue, and the gene expression results of Andersen et al. (2010) that suggest 2 ppm formaldehyde exposure is not likely to increase genotoxic and cytotoxic effects in the nose or in any other tissue, the probability that there is enough damage in the NALT to lead to further carcinogenic manifestations beyond the nose is likely very small at typical human exposure concentrations. Further, the level of damage required to reach quantitative plausibility would likely result in other manifestations in the nose, such as chloromas, which are rarely observed.

In another study, Neuss et al. (2010) show that human nasal epithelial cells pre-exposed in vitro to high concentrations of formaldehyde do not cause DNA damage (DPX) in co-cultivated isolated human lymphocytes, lending further support that formaldehyde that has entered the nasal epithelial cells does not move beyond these cells to damage other cells in close proximity, such as progenitor stem cells in the nasal mucosa.

Zhang et al. (2009) cite a study by Murell et al. (2005) in support of the NALT mode of action, since this study provides some support for the ability of rat olfactory epithelial cells to repopulate hematopoietic tissue in bone marrow of irradiated rats. The olfactory mucosa stem cells used in the Murell et al. (2005) study, however, were tested for their ability to repopulate ablated irradiated rat bone marrow. As discussed in more detail below, a number of studies (McKinney-Freeman and Goodell, 2004; Abkowitz et al., 2003; Edgren et al., 2007) suggest that the majority of circulating stem cells do not efficiently home back to bone marrow under homeostatic conditions.

Overall, the weight of evidence does not support the proposed mode of action that formaldehyde exposure, at reasonably expected concentrations in humans, targets stem cells in the NALT, such that these cells would then be released into the circulation to home back to the bone marrow to cause leukemia.

##### 6.3.1.3. Circulating peripheral HSCs

Zhang et al. (2009) propose another mode of action for formaldehyde-induced leukemia, suggesting that formaldehyde could move beyond the nasal tissue into the circulation where it may transform circulating HSCs that could travel back to the bone marrow.

As discussed already, many studies have examined the cytogenetic effects of formaldehyde in human PBLs as a means for examining distant-site toxicity, but these data are conflicting, with both positive and negative results. In addition, controlled animal studies did not find any significant genotoxic effects (SCE, MN, or CA) in PBLs of rats exposed to high levels of formaldehyde (15 ppm) (Kligerman et al., 1984; Speit et al., 2009). Furthermore, although it is not an unreasonable assumption, observations from studies of circulating blood lymphocytes should not necessarily be taken to mean that the same effects will occur in circulating stem cells that then could transition to leukemia. Only one study to date has examined whether cytogenetic effects in cultured hematopoietic progenitor cells from peripheral blood were increased in workers exposed to formaldehyde (Zhang et al., 2010b), and as discussed in more detail below, there are several problems with interpretation of this study. Therefore, interpretation of the positive cytogenetic findings (beyond the nasal mucosa) in humans, particularly in the context of leukemia, is problematic.

First, as discussed earlier, there is a large body of evidence suggesting that inhaled formaldehyde does not move beyond the nasal respiratory mucosa to cause genotoxicity at distant sites, including lymphocytes (Heck and Casanova, 2004; Pyatt et al., 2008; Golden et al., 2006; Schmid and Speit, 2007; Speit et al., 2009; Lu et al., 2010, 2011; Moeller et al., 2011; Neuss et al., 2010). Although Shaham et al. (1996, 1997, 2003) reported increased levels of protein-associated DNA (presumed to be DPX) in the lymphocytes of hospital workers (laboratory assistants and technicians, physicians, orderlies, and pathologists), as discussed by Heck and Casanova (2004) and Pyatt et al. (2008), there are many problems with these studies. For example, the authors claimed that DPX could be detected down to 0.001 mM; however, their data do not provide any evidence of a concentration-response relationship for DPX below 0.3 mM. Further, Shaham et al. (1996, 1997) indicate that DPX are persistent and can accumulate in lymphocytes. Their data, however, do not support their assertion and are contradictory to studies showing the rapid removal of DPX from formaldehyde-exposed human blood in culture (Schmid and Speit, 2007), and from the nasal respiratory mucosa of rats exposed to formaldehyde via inhalation (Heck and Casanova, 2004). Further, with regard to chromosomal aberrations observed in PBLs, as shown by Schmid and Speit (2007), SCEs are formed from DNA synthesis through DPX during S-phase in human blood cultures. These results suggest that, given the rapid removal of DPX, it is unlikely that a sufficient amount of formaldehyde-induced DPX would persist through DNA replication in occupationally exposed workers. This further suggests that reported SCE frequencies in PBLs of workers exposed to formaldehyde are unrelated to any formaldehyde exposure. The authors extend this argument for other cytogenetic events as well (MN and CA).

Second, interpretation of many of the human PBL studies of formaldehyde-exposed workers is limited due to the lack of reliable exposure information and potential confounding by exposures to other chemicals in the workplace or other factors that may impact background levels of CA and MN. Several studies (Battershill et al., 2008; Iarmarcovai et al., 2008, 2007) suggest that many factors, including age, gender, smoking status, alcohol consumption, disease conditions and infections, physical exercise, and vitamin B12 and folate status impact background levels of CA and MN in PBLs (albeit some factors stronger than others). Battershill et al. (2008) suggest that the evaluation of PBLs as genotoxicitybiomarkers is complex, requiring good exposure data, appropriate stratification of exposed groups, and appropriate statistical power. Given the general limitations in the human PBL studies, it is not surprising that the results with respect to formaldehyde are inconsistent.

Third, observations from studies of circulating blood lymphocytes should not necessarily be taken to mean that the same effects will occur in circulating stem cells that then could transition to leukemia. In fact, CA and MN in PBLs are associated with many types of cancers, and they appear to be a general marker for increased cancer risk, not specific to leukemia (Bonassi et al., 2008; Murgia et al., 2008). In these studies, it is noteworthy that increased CA in PBLs are not associated with occupational exposures to genotoxic agents. Further, as discussed in Pyatt et al. (2008), there are many commonly used drugs with clastogenic properties in vitro and in vivo (methotrexate), and in human lymphocytes in vitro (including antibiotics metronidazole, trimethoprin, and hydrochlorothiazide). Therefore, there is limited value in using clastogenic effects in human lymphocytes as being predictive of leukemic potential.

Only one study (Zhang et al., 2010b) reports increased cytogenetic effects (aneuploidy) in cultured myeloid progenitor cells from 10 workers exposed to formaldehyde (mean of 2 ppm). Zhang et al. (2010b) report an increased loss of chromosome 7 (monosomy 7) and gain of chromosome 8 (trisomy 8) in exposed relative to the unexposed control group. There are several problems, however, with this study.

- First, the study group was very small (10 exposed vs. 12 control) and the results were pooled. Individual results for monosomy 7 and trisomy 8 should have been provided so that the exact nature of aneuploidy could have been assessed on an individual basis, and so it would be clear whether there was a consistent increase for all subjects, or if some were much higher than others, or if some had just one change or both, etc.
- Second, were other chromosome changes looked for and not found? Or did the authors only look for these particular changes? It is not clear, as there is no discussion beyond monosomy 7 and trisomy 8. This is particularly relevant because, although aneuploidy of chromosomes 7 and 8 have been shown to be associated with leukemia (Johnson and Cotter, 1997; Rowley, 2000; Paulsson and Johansson, 2007), they are not the only chromosome changes that are associated with the disease. In fact, as discussed in Johnson and Cotter (1997) and Paulsson and Johansson (2007), monosomy 7 and trisomy 8 are not likely to be initiating events in leukemogenesis, and trisomy 8 alone is not sufficient for leukemogenesis. Trisomy 8 has been shown to occur as a secondary change to primary inversions of other chromosomes (i.e., chromosomes 9 and 11) (Paulsson and Johansson, 2007).
- Third, Zhang et al. (2010b) note a high monosomy 7 incidence in the controls and indicate that this could be due to artifactual chromosome loss during meta-phase spread preparation; therefore, there is inherent bias in the sampling technique that could bias the results.
- And finally, myeloid associated monosomy 7 and trisomy 8 have been shown to be correlated with other exposures. Smoking has been shown to cause trisomy 8 (Paulsson and Johansson, 2007; Moorman, 2002), and other occupational exposures (e.g., pesticides, organic solvents, and petroleum compounds) have been shown to cause monosomy 7 (Johnson and Cotter, 1997). A recent formaldehyde occupational exposure study (Iarmarcovai et al., 2007), where increased MN were observed in exposed vs. controls, found that alcohol consumption had a potential confounding effect on chromosome loss. Approximately 40% of the control and exposed subjects in the Zhang et al. (2010b) study were smokers, and about 20% in each group consumed alcohol. Although the percent smokers and alcohol consumers was roughly the same in the exposed and control groups, there was no attempt to determine the degree of smoking or alcohol consumption among the subjects. Therefore, potential confounding from these exposures could have biased the results, particularly given the small sample size. Individual data could provide more insight into potential confounding associations.
- Overall, given the small study group, lack of a thorough examination of chromosomal effects, potential confounding of observed effects (i.e., other potential exposures, smoking, alcohol consumption), and the possibility of artifactual chromosome loss during sample preparation, it is possible to attribute the chromosomal changes reported by Zhang et al. (2010b) to chance.

Given the strong evidence that inhalation exposure to formaldehyde (at reasonably expected concentrations for humans) does not increase the level of formaldehyde in the blood and does not cause DNA damage and cellular transformation beyond the nasal respiratory mucosa, in combination with the inconsistent effects observed in PBLs of humans occupationally exposed to formaldehyde (likely due to confounding and lack of good formaldehyde exposure information), and the fact that there is little support for the use of PBLs as a marker for effects in HSCs and leukemia, the PBL data from formaldehyde occupation studies, taken as a whole, provide little (if any) support for the proposed modes of action for formaldehyde as a leukemogen. Finally, the recent study by Zhang et al. (2010b) is hampered by potential confounding, a small study group, sampling artifacts, and lacks reporting of critical information, such that the reported chromosome changes in this study are impossible to interpret.

Therefore, the weight of available evidence does not support the proposed mode of action that formaldehyde might target circulating HSCs that might then home back to the bone marrow to cause leukemia.

#### 6.3.2. Formaldehyde exposure would have to be very high to induce DNA damage above endogenous levels in the bone marrow, NALT, or circulating HSCs, and would likely be associated with a high degree of irritation

As discussed already, there is a large body of supportive evidence that inhalation exposure to formaldehyde at reasonably expected concentrations for humans (less than 2 ppm) does not result in increased blood levels of formaldehyde (Heck and Casanova, 2004; Franks, 2005; Andersen et al., 2010), likely due to normal metabolic processes that prevent formaldehyde from readily entering the circulation. Further, there is evidence to suggest that DNA damage does not occur in the blood or bone marrow of animals even at concentrations as high as 6-15 ppm. Schmid and Speit (2007) propose that, due to the rapid removal of DPX, very high concentrations of formaldehyde would be required (higher than what would be expected for humans occupationally exposed to formaldehyde) to produce enough DPX that would persist until DNA replication could lead to a permanent genotoxic effect (i.e., SCE, CA, or MN).

It is important to consider these concentrations in the context of what concentrations of formaldehyde are known to cause sensory irritation. Arts et al. (2006) conducted a review of the formaldehyde respiratory irritation and carcinogenicity data and found that overall, formaldehyde sensory irritation is first observed at 1 ppm in animals and humans, with eye and nasal irritation occurring at concentrations ≥1 and ≥2 ppm, and throat irritation occurring at ≥3 ppm, and more severe irritation occurring at concentrations ≥6 ppm. Therefore, sensory irritation occurs at concentrations well below those that would likely be necessary to cause sufficient DNA damage in blood, NALT, or bone marrow, and therefore the formaldehyde exposure concentrations necessary to cause such DNA damage would likely not be tolerated by humans.

#### 6.3.3. Circulating HSCs may not readily home back to healthy bone marrow to cause leukemia

A critical assumption in the proposed modes of action that formaldehyde either targets stem cells in the NALT or circulating in the blood is that these damaged cells will travel back to and become incorporated into the bone marrow where they could then cause leukemia. Although much of the evidence suggests that these proposed modes of action are not biologically plausible, there is still a general assumption that if the exposure conditions were such that even one cell was transformed, either directly in circulating HSCs or in the NALT and then released into the circulation, that this cell would then readily home back to the bone marrow. The current evidence is not clear, however, with regard to this assumption for people with healthy bone marrow (McKinney-Freeman and Goodell, 2004; Abkowitz et al., 2003; Abrams et al., 1980; Wright et al., 2001; Schulz et al., 2009), which would be the majority of the population for which the regulatory outcome of these studies and proposed mechanisms would seek to protect. And, in fact, a number of studies suggest that the majority of circulating HSCs may not efficiently home to bone marrow.

For example, using genetically marked parabiosed CD45 congenic mice (surgically joined and sharing a common circulation), McKinney-Freeman and Goodell (2004) found that although there was a small percent of partner-derived stem cells present in the bone marrow, the majority of animals were not stably engrafted with partner HSCs when tested for functional HSC activity, suggesting that although a small percent of circulating HSC can reenter the bone marrow during homeostasis (i.e., in the absence of cytokine mobilization), this reen-trance is transient and unstable, and functional HSCs do not persist in the bone marrow after returning from the circulation. The results of this study are supported by Abkowitz et al. (2003), who also used genetically marked parabiosed mice in a similar experiment and found similar results. These results suggest that HSC homeostasis is primarily maintained by endogenous stem cells in the bone marrow, and not from the return of stem cells from the circulation. The authors propose that “[bjecause the HSC replication rate is high [in the bone marrow], the new HSCs outnumber the few HSCs entering the marrow from the peripheral blood. Once HSCs exit bone marrow, their lifespan in the circulation is extremely short, contributing to the competitive advantage of endogenously generated cells.”

There may be additional support for the idea that circulating HSCs do not readily home back to bone marrow in that that there is no evidence that blood transfusions from precancerous (leukemia) blood donors are associated with increased risk of leukemia in recipients (Edgren et al., 2007). It is not unreasonable to assume that blood donors who were later diagnosed with leukemia had circulating progenitor cells that had genetic damage or were transformed. Although it would need to be confirmed that preleukemogenic individuals have precancerous circulating HSCs, if preleukemogenic cells did exist in a blood donation, and these cells readily home back to bone marrow, Edgren et al. should have seen an increased risk of leukemia in the blood recipients, but they did not. The authors cite other studies that were inconclusive with regard to this question.

There are conflicting studies that appear to suggest that HSCs do efficiently home to bone marrow under homeo-static conditions (Wright et al., 2001). A recent review by Schulz et al. (2009), however, indicates that the mechanisms involved in the control of hematopoietic stem or progenitor cell function remain largely unknown. The authors indicate that, in addition to recirculation to the bone marrow, HSCs migrate to peripheral tissue during inflammation to respond to tissue damage. Therefore, it appears that there is much to learn with regard to mechanisms involved in homing of HSCs to bone marrow under homeostatic conditions. Consequently, the assumption that damaged HSCs or NALT stem cells would readily return to bone marrow where they could then cause leukemia should be questioned, and further studies are necessary to assess the extent to which this might occur under homeostatic conditions.

Therefore, aside from the questions put forth with regard to the implausibility that exogenous formaldehyde could sufficiently damage NALT stem cells or circulating HSCs, there are clearly also questions regarding the extent to which these stem cells would then migrate back to the bone marrow. Consequently, these studies add further to the questions regarding the plausibility of the proposed modes of action. Moreover, it is critical that we try to better understand HSC trafficking in and out of bone marrow under normal physiological conditions before accepting any mode of action that relies so heavily on this mechanism.

### 6.4. Summary

As a whole, the available formaldehyde toxicokinetic, mode-of-action, and genotoxicity studies provide little evidence for support of the account that formaldehyde exposure is causally associated with leukemia. The ad hoc assumptions that have been put forth in support of the three proposed modes of action are not consistent with the full body of evidence. To support the proposed modes of action, one must assume,

1. with regard to targeting circulating hematopoietic stem cells, that formaldehyde can move beyond the nasal respiratory mucosa to increase levels in the blood to a sufficient degree that would result in carcinogenic initiation of progenitor cells, and the weight of evidence does not suggest this, at least for levels to which humans are likely to be exposed and that could be tolerated (due to irritation at higher levels of exposure);
2. with regard to targeting bone marrow, that formal dehyde can travel in its hydrated methanediol form to the bone marrow where it will be in equilibrium with free formaldehyde that can cause DNA damage and cellular transformation, even though this is biologically implausible and the weight of evidence strongly suggests that exogenous formaldehyde does not cause DNA damage in any tissue other than the nasal respiratory mucosa;
3. with regard to targeting stem cells in the NALT, that formaldehyde somehow moves beyond the nasal respiratory mucosa and causes sufficient damage to nasal stem cells, such that further carcinogenic manifestations could occur (leukemia), without causing any nasal lymphomas or chloromas in the nasal tissue, even though it is biologically and quantitatively implausible that the level of damage likely required in the NALT to cause further carcinogenic manifestations would not also lead to chloromas and nasal lymphomas;
4. with regard to targeting circulating HSCs, that formal dehyde somehowmoves beyond the nasal respiratory mucosa and causes DNA damage or transformation of circulating stem cells, even though the majority of evidence provided as support for this mechanism is from a large number of inconsistent PBL cytogenetic studies of formaldehyde-exposed workers and likely confounded by exposures to other chemicals in the workplace or by effects from smoking or alcohol consumption (in addition to the assumption that chromosomal effects in PBLs are good biomarkers for effects in HSCs and leukemia, and there is little support for this in the literature); or
5. the chromosome aneuploidy in cultured myeloid progenitor cells of 10 formaldehyde exposed workers reported in the Zhang et al. (2010b) study somehow suggests that these workers may be at a higher risk for leukemia, even though this study is hampered by potential confounding, a small study group, sampling artifacts (e.g., possible artifactual chromosomal loss during metaphase spread preparation), and lacks reporting of critical information, such that the reported chromosome changes in this study are impossible to interpret; and
6. even if one accepts, or it is somehow shown, that formaldehyde is capable of transforming stem cells in the NALT or circulating HSCs, that these cells will then readily home back to the bone marrow, even though currently there is evidence to suggest that these cells infrequently home back to healthy bone marrow.

Moreover, beyond the lack of support provided by the current mechanistic weight of evidence, as discussed, the epidemiology data, human and animal hematotoxic-ity data, and animal leukemia studies do not provide any support for the proposed modes of action for formaldehyde leukemogenesis.

It is worth pointing out an inconsistency with respect to data that have been put forth in the context of the three proposed modes of action for formaldehyde leukemogenesis. That is, reported observations of formaldehyde-induced hematotoxicity have been generally discussed as indicating a causal association with leukemia, and the proposed association has been discussed in the context of three possible modes of action. Bone marrow toxicity, however, can only occur if the chemical interacts directly with the bone marrow, which would only happen in the proposed mode of action that targets bone marrow. If the alternative modes of action are plausible (targeting circulating hematopoietic stem cells or NALT stem cells), formaldehyde would not be expected to cause hematotoxicity because it would not be directly acting on bone marrow. Instead, it likely would not be until tumor formation in bone marrowthat one would expect a change in blood cell counts (likely increase in WBCs). That is, hematotoxicity would not be expected to occur in the exposed workers in the Zhang et al. (2010b) study if the mode of action was through formaldehyde damage to circulating progenitor cells or NALT stem cells. Interestingly, there are no other leukemogens that do not also show hematotoxicity, and therefore these leukemogens likely act by directly damaging the bone marrow. So, acceptance of one of these two modes of action (targeting circulating HSCs or NALT stem cells) suggests formaldehyde acts via a completely different mechanism from other leukemogens (i.e., in the absence of hematotoxicity), further suggesting biological implausibility.

Finally, it is informative to consider the phenomenon of apparent dependence of increased leukemia risk in certain epidemiology studies on peak exposure rather than on average or cumulative exposure. As we described in Section 4, in the NCI industrial worker cohort, Beane Freeman et al. (2009) found that the presence in a worker's career of peak exposures >4 ppm was associated with increased leukemia risk. In that section, we questioned whether this dependence on peaks was merely a matter of choosing among several dose metrics considered based on its outcome. But if the dependence on peaks is a real effect—if it is a discovery of the epidemiology investigations—there should be some correspondent peak-dependent aspects evident when proposed modes of action are investigated. It is not clear from consideration of the modes of action that have been proposed how such a peak dependence could work. If formaldehyde has to leave the respiratory tract and be redistributed to distant tissues such as marrow, the sharpness of a peak of exposure would be greatly attenuated as the absorbed formaldehyde mixed into the general circulation. Similarly, if susceptible cells are to migrate from marrow to the respiratory tract and back to the marrow, or from NALT in the respiratory tract to the marrow—processes that are hypothesized to be occurring at a low but ongoing level—it is not clear how peak inhalation exposures could have special effect. One would expect associations with other measures of exposure besides peak if this were the case. If genotoxic modes of action are proposed (so as to form the basis for concern regarding potential cancer risks to people experiencing low environmental exposures), the accumulation of risk of transforming mutations similarly must be an ongoing process that does not readily explain the apparent dependence on peak exposure as noted by Beane Freeman et al. (2009). In our view, such considerations illustrate the importance of integrating weight of evidence across disciplines, not just in combining conclusions from different disciplines, and in using a hypothesis-based framework to assess the consistency of analyses and their interpretations with mutual illumination across disciplines.

## 7. Discussion

The most current draft of the US EPA assessment of formaldehyde's human health risks (US EPA, 2010) states, “[h] uman epidemiological evidence is sufficient to conclude a causal association between formaldehyde exposure and ... all leukemias, myeloid leukemia and lymphohe-matopoietic cancers as a group,” but it also notes that “[ljimited evidence from animal bioassays is available to support the conclusion from human epidemiologic data that formaldehyde causes some types of lymphohe-matopoietic cancers.” As is clear from the US EPA statement, these conclusions are backed by evaluations based initially on a judgment about the human data alone, conducted according to the approaches that epidemiologists use to evaluate whether the patterns observed among human studies of apparent associations between inhaled formaldehyde and lymphohematopoietic cancers are, in the judges' view, sufficiently indicative of a causative process. It is only afterward that the compatibility of this conclusion with information from animal studies or mode-of-action data is considered, and, if the human-data-only conclusion is one of causation, the presence or (as in the case of formaldehyde) lack of additional support is noted.

The most recent update of the IARC monograph (IARC, 2009) states that, with regard to formaldehyde, “the epidemiological evidence on leukaemia has become stronger, and new mechanistic studies support a conclusion of *sufficient evidence* in humans. This highlights the value of mechanistic studies, which in only 5 years have replaced previous assertions of biological implausibility with new evidence that formaldehyde can cause blood-cell abnormalities that are characteristic of leukaemia development.” IARC further states that” [t]he Working Group was almost evenly split on the evaluation of formaldehyde causing leukaemias in humans, with the majority viewing the evidence as *sufficient* for carcinogenicity and the minority viewing the evidence as *limited.* Particularly relevant to the discussions regarding sufficient evidence was a recent study accepted for publication which, for the first time, reported aneuploidy in blood of exposed workers characteristic of myeloid leukaemia and myelodysplas-tic syndromes with supporting information suggesting a decrease in the major circulating blood cell types and in circulating haematological precursor cells.” Although the IARC monograph highlights mechanistic studies, it appears that “viewing the evidence as sufficient” stems predominantly from one human occupational study (likely Zhang et al., 2010b, although not cited by IARC, 2009).

Our concern with such a process is that it fails to appreciate the role that animal, toxicokinetic, and mode-of-action data can and should have, not just in the overall conclusion, but in the interpretation of the meaning of the epidemiological data themselves. If one concludes that the epidemiological data show causation, then there is an implicit conclusion that some mechanism for this causal process is not merely conceivable or not yet disproven, but it must actually exist. If it is firmly concluded that something is causal, it must also be firmly concluded that a means for that causation exists, even if it is not named. If animal studies or other mode-of-action studies are not in concordance with the human-data-only conclusion (epidemiology and the key mechanistic occupational study referenced by IARC), acceptance of the apparent causation in humans necessarily includes a further conclusion that the discordance is explicable—that the causes invoked for the human data either do not operate in animals or, for some scientifically plausible reason, are not manifested in observable consequences.

Our HBWoE approach calls attention to this and recognizes that the weight-of-evidence evaluation should evaluate these subsidiary conclusions about the plausibility of human mechanisms and their concordance or lack of concordance with mechanisms in animals. It is important to evaluate these subsidiary conclusions explicitly rather than leave them implicit. It is particularly important when, as is the case for formaldehyde, our understanding of these other aspects is not merely non-supporting of the human-data-only conclusion but actually conflicts with it. If inhaled formaldehyde is indeed a human leukemogen, then something about what is commonly understood, related to possible mechanisms and their potential operation in humans and rodents, is in error. Conversely, if it is indeed right to doubt the scientific plausibility of suggested mechanisms, their operation in human exposures, and their lack of operation in animal studies, then it is wrong to interpret the patterns among human studies as indicative of causality. Because the epidemologists' evaluation of causality from the human data entails judging how well a common causal explanation is supported by the array of observations compared to alternative explanations that attribute the apparent patterns to other, non-causal influences (such as chance and confounding), the scientific plausibility of the causal interpretation of the human-data patterns in view of other, non-epidemiologic data is an important part of a sound evaluation.

We have attempted to carry out a more complete evaluation across scientific disciplines for the case of inhaled formaldehyde and hematopoietic cancers in humans. In our reading of the weight of evidence, the conclusion that formaldehyde can cause such effects is not well supported.

In summary, the HBWoE evaluation for formaldehyde considers two alternative accounts. One account consists of acceptance of the epidemiology evidence as sufficiently compelling that, even in the face of weak hema-tological and carcinogenic evidence in animals and weak and inconsistent hematological evidence in humans, one of the proposed modes of action for formaldehyde leukemogenesis must be right, since its manifestations as increased leukemia risks are seen in the human studies. Moreover, the arguments against the biological plausibility of these modes of action must in some way be incorrect. Acceptance of this account is associated with many unanswered questions and post hoc explanations for how the current data should be interpreted as supporting it. This account requires that one accepts the reported exposure and disease information in the epidemiology studies as true, even though the lack of precise exposure data likely led to exposure measurement error and/or exposure misclassification that could have biased results, and disease misclassification in these studies likely led to unreliable risk estimates. It requires that all the many human studies that failed to show increased leukemia risk did so for plausible reasons, such that the lack of effects does not contradict formaldehyde's asserted general property of leukemogenicity. This account requires that one accepts an existence of an exposure-response relationship, despite the lack of consistently observed exposure-response associations within or among the epidemiology studies. It requires that one accepts the post hoc explanation of short latency for the increased risks associated with peak exposure observed in the NCI industrial worker cohort with follow-up through 1994, but not when follow-up was continued through 2004, even though this does not explain how this trend was not observed in the NCI embalmers cohort (Hauptmann et al., 2003; Walrath and Fraumeni, 1983, 1984) or garment workers cohort (Pinkerton et al., 2004), in whom risks were only observed with exposures over 20 years. This account requires that, although the epidemiology data were statistically analyzed in many different parallel ways with many finding no significant association, one chooses to focus only on the few marginally significant findings while ignoring the others as part of the evidence as a whole. For example, in the NCI industrial worker cohort, associations were reported with peak exposures, but there was no a priori reason to focus on peak exposures. These results should at most be treated as hypothesis-generating observation to be tested empirically. Otherwise, it is post hoc and arbitrary.

Moreover, this account (that formaldehyde is causally associated with leukemia) requires inclusion of an explanation as to why controlled animal experiments fail to show hematological or leukemogenic effects at high formaldehyde exposure concentrations (6 to 15 ppm). That is, what is being argued to be happening in humans (to allow the leukemogenic effect) must for some reason not be happening in the experimental animals, or else they would have been seen to have parallel hematotoxic and leukemogenic effects, as well as evidence of other consequences of operation of the proposed modes of action. It is not beyond reason that a leukemogenic effect of formaldehyde might be confined to humans, but there has been no explanation offered for why this might be so. Further, the proposed modes of action that would enable an effect in humans do not have any evident basis to be absent in rodents—indeed, some of the elements (migrating stem cells, effects on NALT), both consistent with or contrary to this account, are based on rat data. As it stands, the reasons for rodents not being subject to the proposed causative processes in humans constitutes an unstated corollary— one without empirical support or plausible basis—to the theories of human leukemogenesis of formaldehyde.

One needs to account for the inconsistencies among studies regarding the hematological evidence in humans; if there is an effect of formaldehyde inhalation, then what reasons are proposed for why it is not seen in many of the studies (and not seen at all in animals)? Only some of the hypothesized modes of action entail hematotoxicity, and so a proposal of its role in human leukemia depends on the particular variety of proposed mode of action being considered, with observations in favor of one mode tending to contradict other modes and hence in need of explanation for why such conflicts are not refuting. Finally, because of the weak and inconsistent epidemiological and toxicological evidence for a causal association, this account requires that one rely heavily on the truth of toxi-cokinetic and mechanistic hypotheses that permit a plausible biological mode of action. To accept this account as true, one must accept that somehow formaldehyde can move beyond the nasal respiratory mucosa to ultimately cause DNA damage and cellular transformation in bone marrow, circulating hematopoietic stem cells, or the NALT, even though there is a large body of evidence to suggest that inhaled formaldehyde (at reasonably high exposure concentrations for humans, 2 ppm) does not increase levels in the blood and does not cause DNA damage in cells and tissues beyond the nasal respiratory mucosa to a sufficient degree that would manifest as leukemia. If one is to conclude that formaldehyde is a “known” human leukemogen, one must assert not only that these hypothesized modes of action are conceivably true but that it is indeed known that one of them is true, for otherwise an essential and utterly necessary element of the causal conclusion is missing.

For this account (formaldehyde is causally associated with leukemia), there is a very large degree of ad hoc argument. That is, the elements of this account are chosen so as to fit the hypothesis already put forth, not based purely on an evaluation of the weight of the evidence as a whole and how it may (or may not) support the proposed hypothesis. Consequently, alternative accounts need to be considered.

An alternative, and contrasting, account is that it is not possible for formaldehyde to move beyond the nasal respiratory mucosa to cause systemic DNA damage and cellular transformation (in the bone marrow, circulating hematopoietic stem cells, or the NALT), and therefore there is no biologically plausible mechanism for formaldehyde leukemogenesis. This is supported by a large body of toxicokinetic and mechanistic data in animals and in vitro, and by inconsistent cytogenetic peripheral blood lymphocytes data in humans that are likely confounded by other exposures and a lack of reliable formaldehyde exposure information, in addition to the fact that there is little evidence to support the use of peripheral blood lymphocytes data as a biomarker for effects in hematopoietic stem cells or for leukemia. Further, the lack of toxicokinetic and mechanistic biological plausibility is supported by the largely negative toxicological evidence and a significant number of null epidemiology findings, which are considered under this account to be the true results, whereas those relatively isolated and unrepeated positive results are considered as false positives attributable to confounding by other exposures or to chance. If this account is true, an association between inhalation of formaldehyde and leukemia would be understood as not plausible for humans, and the few positive associations that have been observed would be attributed to alternative explanations (i.e., to other chemical exposures in the workplace, or lifestyle-related exposures such as smoking or alcohol consumption, or simply to chance).

In comparing these two accounts, neither is proven or disproven, but when assessing the weight of the available evidence in support of either account, it is clear that the first account requires far more ad hoc assumptions and post hoc explanations. In the first account, the inferences regarding potential human risk are not coming from the data themselves, but from assumptions invoked after the fact to fit the hypotheses put forth and without the evidence that would tie the weak epidemiological, toxicological, and mode-of-action data causally to formaldehyde inhalation exposure. Therefore, the weight of evidence for this account (i.e., exposure to formaldehyde in air is causally associated with leukemia in humans) is weak in comparison to the more substantial weight of evidence supporting the lack of a causal association.
